# Impact of Fluorine Pattern on Lipophilicity and Acid–Base Properties of 2‑(Thiofluoroalkyl)pyridines: Insights from Experiments and Statistical Modeling

**DOI:** 10.1021/acs.jmedchem.4c03045

**Published:** 2025-02-17

**Authors:** Miguel Bernús, Gonzalo D. Núñez, Will C. Hartley, Marc Guasch, Jordi Mestre, Maria Besora, Jorge J. Carbó, Omar Boutureira

**Affiliations:** † Departament de Química Analítica i Química Orgànica, 16777 Universitat Rovira i Virgili, 43007 Tarragona, Spain; ‡ Departament de Química Física i Inorgànica, 430222 Universitat Rovira i Virgili, 43007 Tarragona, Spain

## Abstract

Lipophilicity and acid–base properties are two key aspects of the optimization of a compound in drug discovery. Using ^19^F NMR, we experimentally determined the log *D* ^7.4^ of a wide array of 2-thiofluoroalkyl (SR_F_) and 2-sulfonyl fluoroalkyl (SO_2_R_F_) substituted pyridines and the p*K*_a_ values of their protonated counterparts. Statistical modeling based on constitutional and DFT descriptors provided further insights into the structure–property relationship, explaining the experimental observations and predicting log *D* ^7.4^ values. Our results highlight the influence of fluorination topology in SR_F_ fragments and demonstrate the dual effect of fluorine on molecular polarity, increasing the hydrophobic surface and the polarity of the sulfur moiety. By analyzing methyl- and ethyl-derived fragments, we found a gradient in log *D* ^7.4^ values influenced by the oxidation state of the sulfur atom and fluorination pattern. Our findings emphasize the context-dependent impact of fluorination and offer insights to better understand the impact of thiofluoroalkyl chains on these physicochemical properties.

## Graphical abstract

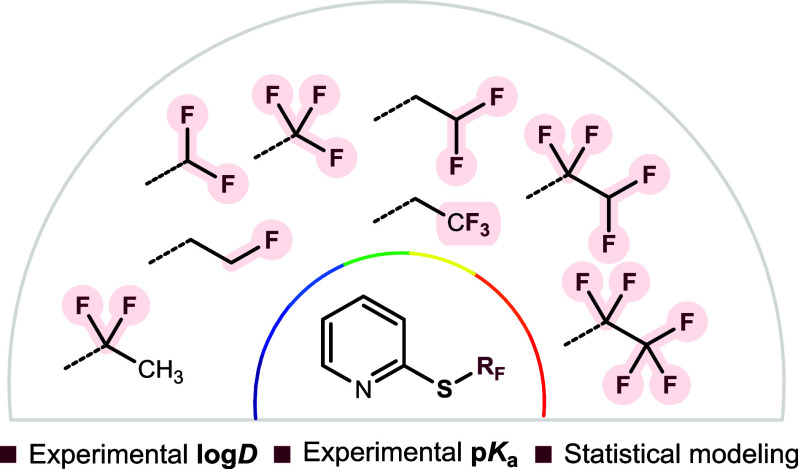

## Introduction

Sulfur and fluorine are common elements in the design of new fragments in agrochemistry^1,2^ and drug discovery.^3–6^ Of the top 200 small molecule drugs, approximately 25% contain at least one sulfur atom,^7^ and 20% of all commercial drugs contain fluorine.^6^ The introduction of fluorine atoms can markedly alter the biological potency and pharmacokinetic properties of a compound.^8–10^ On the other hand, sulfur offers versatility in its breadth of functionalities, owing to its ability to adopt a range of oxidation states.^11^ Thiofluoroalkyl chains have recently attracted interest as an emerging motif in drug discovery that may complement substituents such as trifluoromethyl (CF_3_) and other polyfluorinated alkyl chains (Figure 1A). The combination of the polarizable sulfur atom with strongly electron-withdrawing, yet lipophilic, fluoroalkyl chains has the potential to offer modulation of lipophilicity and polarity (Figure 1B).^12^ However, currently there is a lack of data on the combined effects of the fluorination degree and substituent pattern on such thiopolyfluoroalkyl motifs, despite the recent development of methods for their incorporation.^13–15^ This situation is rapidly changing^16–26^ and will no doubt continue to change as greater knowledge is acquired. While the effect of the fluorination pattern on the lipophilicity of alkyl chains has been studied in great detail,^27,28^ our aim was to evaluate the influence of fluorination on two key physicochemical properties, lipophilicity (log*D* ^7.4^ *)* and basicity (p*K*_a_ of conjugated acid), of methyl and ethyl thiofluoroalkyl (SR_F_) fragments and their oxidized sulfonyl fluoroalkyl analogues (SO_2_R_F_) in *ortho*-substituted pyridines.

**Figure 1:**
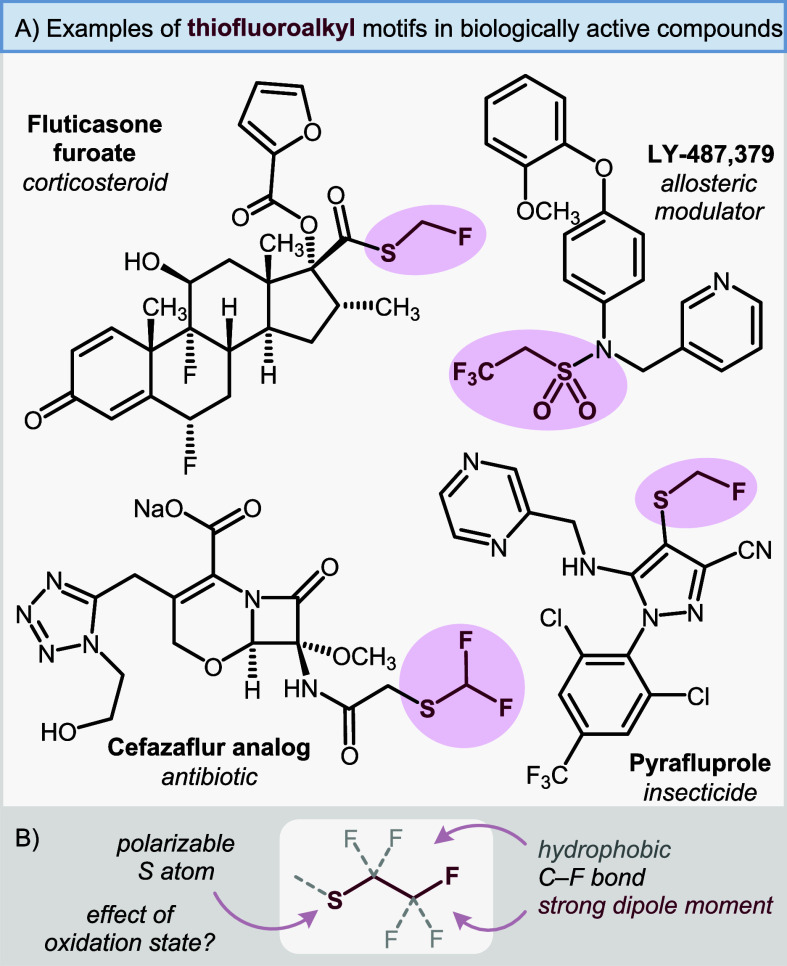
(A) A selection of biologically active compounds bearing the thiofluoroalkyl motif.^1,6,29,30^ (B) Modulation of fluoroalkyl chains.

## Results and Discussion

### Lipophilicity

Motivated by its prevalence in many biologically active compounds, we selected pyridine as a heterocyclic anchor,^31^ with 2-(thioalkyl)­pyridine as our model system.^32^ *Ortho*-substituted mercaptopyridines were of particular interest since this substitution pattern enables greater electronic influence of the proximal thiopolyfluoroalkyl chain on the basicity of the heterocyclic nitrogen. A library of pyridines featuring thiofluoroalkyl units of one or two carbons and different degrees of fluorination was synthesized (see the Synthesis section for details).

Log*D* ^7.4^ (pH 7.4) measurements of the 2-(thiofluoroalkyl)­pyridines were conducted according to a method developed by Linclau and coworkers,^33^ a variation on the traditional “shake-flask” partitioning between octanol and water. Together with a fluorinated internal standard of known lipophilicity, simple ^19^F NMR experiments allowed for the calculation of the log*D* ^7.4^ value of the compound of interest through integration of NMR signals in each phase. During our experiments, no difference between log*D* ^7.4^ and log*P* values was observed, probably due to the low basicity of the pyridines. Nonetheless, for the sake of consistency, log*D* ^7.4^ was deemed the appropriate parameter for evaluating lipophilicity.

Incorporation of fluorine onto the thioalkyl chains was compared with the nonfluorinated 2-(methylthio)­pyridine **1**, which has a log*D* ^7.4^ value of 1.69. Difluorinated motif SCF_2_H **2** resulted in a modest increase of lipophilicity to 1.95, while the fully fluorinated SCF_3_ sample **3** exhibited the greatest lipophilicity in the series, with a log*D* ^7.4^ value of 2.13 (Figure 2A). On the other hand, log*D* ^7.4^ measurement of the ethyl series revealed a more complex situation, with no simple additive correlation between the degree of fluorination and lipophilicity.

**Figure 2:**
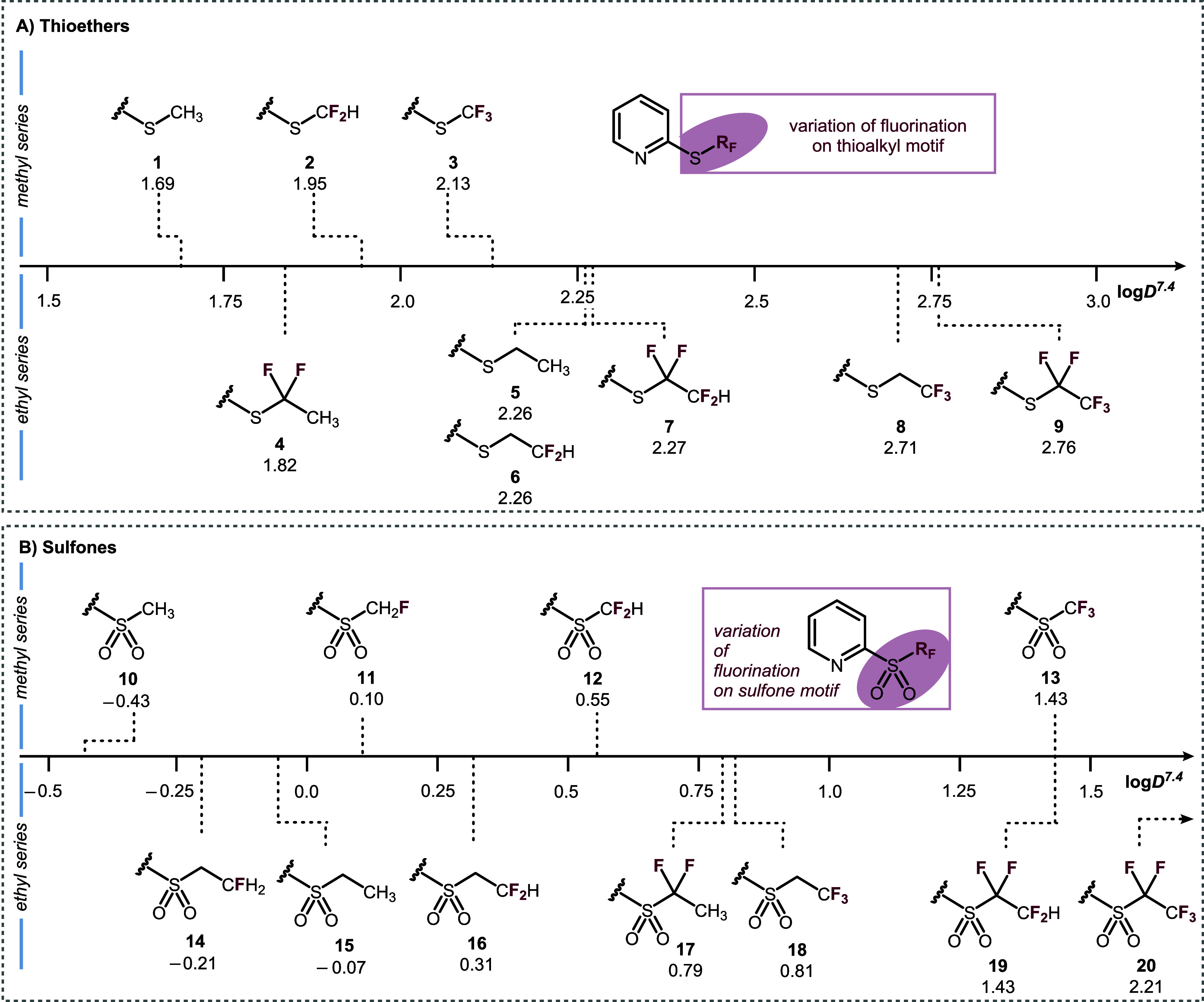
(A) Measured log*D* ^7.4^ values for a range of 2-(thiofluoroalkyl)­pyridines. (B) Measured log*D* ^7.4^ values for a range of 2-(sulfonylfluoroalkyl)­pyridines.

First, the internally difluorinated SCF_2_CH_3_ pyridine **4** has a lower log*D* ^7.4^ value (1.82) than its nonfluorinated parent compound (**5**, SCH_2_CH_3_, log*D* ^7.4^ = 2.26). The decreased lipophilicity upon internal difluorination, also observed by O’Hagan in structurally related thiofluoroalkyl scaffolds, has been a topic of discussion due to its possible multiple conformations in equilibrium.^12^

Interestingly, terminal difluorination (**6**, SCH_2_CF_2_H, log*D* ^7.4^ = 2.26, and **7**, SCF_2_CF_2_H, log*D* ^7.4^ = 2.27) did not change the lipophilicity of the ethyl fragment (**5**, SCH_2_CH_3_, log*D* ^7.4^ = 2.26), with both fluorinated fragments exhibiting the same log*D* ^7.4^ profile as the nonfluorinated parent. However, terminal trifluorination of the ethyl group gave compounds with the greatest log*D* ^7.4^ values (**8**, SCH_2_CF_3_, log*D* ^7.4^ = 2.71 and **9**, SCF_2_CF_3_, log*D* ^7.4^ = 2.76), with the perfluorinated ethyl chain **9** being the most lipophilic. This observation is consistent with Linclau’s studies on polyfluorination of alkyl chains, in which it was shown that while perfluorination and terminal CF_3_ groups generally increase lipophilicity, internal polyfluorination could lead to a decreased log*D* ^7.4^.^28^

A series of sulfone analogues were synthesized to compare the effect of the sulfur oxidation state on the lipophilicity of the fluoroalkyl motifs (see the Synthesis section for details). In each case, the sulfone exhibited a lower log*D* ^7.4^ value compared to its parent thioether (Figure 2B). This is consistent with the incorporation of two polarized S=O bonds that can withdraw electron density from sulfur while simultaneously providing hydrogen-bond donor sites. In fact, Zafrani et al. quantified the H-bond basicity of the sulfone functional group and its impact on lipophilicity.^34^ Across the methyl series, a concomitant increase in log*D* ^7.4^ is observed with increased fluorination. For the ethyl series, greater fluorination also results in increased log*D* ^7.4^ although there are some exceptions. First, substitution of a single terminal C–H bond for a C–F bond (**14**, SO_2_CH_2_CH_2_F, log*D* ^7.4^ = −0.21) results in a lower log*D* ^7.4^ value than its parent compound (**15**, SO_2_CH_2_CH_3_, log*D* ^7.4^ = −0.07). In addition, the internally difluorinated sulfone (**17**, SO_2_CF_2_CH_3_, log*D* ^7.4^ = 0.79) exhibits greater lipophilicity than the terminally difluorinated motif (**16**, SO_2_CH_2_CF_2_H, log*D* ^7.4^ = 0.31), the opposite effect to their thioether analogues **4** and **6** (log*D* ^7.4^ = 1.84 and 2.26, respectively). Comparison between the methyl and ethyl series reveals that insertion of a methylene unit into the SO_2_–CF_2_H or SO_2_–CF_3_ bond results in decreased log*D* ^7.4^ from 0.55 (**12**, SO_2_–CF_2_H) to 0.31 (**16**, SO_2_–CH_2_CF_2_H), and 1.43 (**12**, SO_2_–CF_3_) to 0.81 (**18**, SO_2_–CH_2_CF_3_), respectively.

A key observation is that fluorination of some internal C–H bonds leads to a counterintuitive decrease in log*D* ^7.4^ (e.g., **4** vs **5**). Using basic chemical knowledge, one would expect that H/F replacement at the α-position with respect to sulfide not only increases molecular volume, but also the inductive effects of the fluorine would cause a decrease of the H-bond basicity of the pyridine and the sulfide functional groups. Both effects would contribute to enhancing lipophilicity.^35^ To better understand the key factors that contribute to the observed lipophilicities, we developed a model for the prediction of log*D* ^7.4^ values of fluoroalkyl-substituted 2-thioether and 2-sulfonylpyridines (Figure 3). As a first approximation, a series of regression models were developed using statistical techniques with molecular descriptors obtained from structure and from density functional theory (DFT) calculations.^36^ The first univariate approaches considering either topological polar surface area (TPSA),^37^ quantum polar surface area (QPSA),^38^ or DFT partition energy differences only yielded unsatisfactory models that poorly described the experimental data (Table S8).

**Figure 3:**
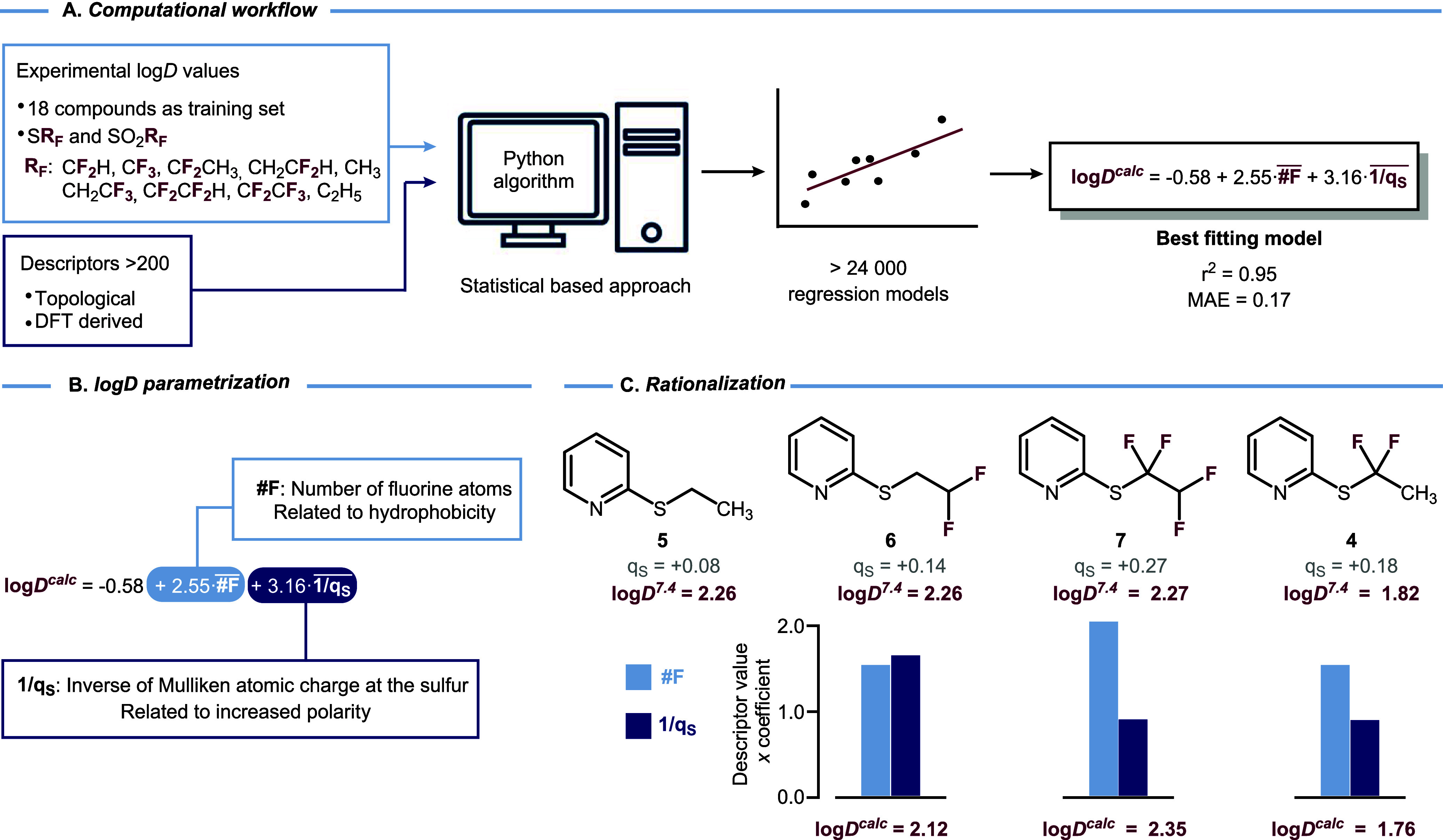
(A) Representation of the computational workflow to obtain fitting models to describe log*D* ^7.4^ behavior of the studied pyridines. (B) Description of the key terms in the best fitting eqs. (C) Comparison of experimental and calculated log*D* ^7.4^ values, degree of fluorination, and Mulliken atomic charges at the sulfur of a selection of 2-SR_F_ pyridines.

To find an adequate lipophilicity model, we performed a correlation analysis between the log*D* ^7.4^ values and >200 descriptors derived for each of the substituted pyridines following the workflow defined in Figure 3A. The data set comprises the thioalkyl molecules in Figure 2A and the corresponding sulfonylalkyl analogues (Figure 2B). For each structure, we performed a conformational sampling, and the lowest-energy conformer was selected to build the regression models (see the Computational Details section and Supporting Information for details). In this case, we did not observe significant differences when using the energy-weighted values of the conformers (Table S9). This conformational analysis also implicitly considers the formation of intramolecular hydrogen bonds (IMHBs) that have been proposed to affect lipophilicity.^34,39–43^ Here, we have characterized IMHBs via noncovalent interaction (NCI) analysis and Bader’s atoms in molecule (AIM) theory for several structures, consisting of the interaction of CF*_x_* H*_y_* groups acting as H-bond donors with the rest of the molecule. Our findings reveal the following order of strengths for the hydrogen bonds interacting with the pyridinyl nitrogen: -S-CF_2_CF_2_H (**7)** > -S-CH_2_CF_3_ (**8)** ≫ -S-CF_2_H (**2)** > -S-CFH_2_ (**21)** > -S-CH_2_CF_2_H (**6)** > -S-CH_2_CFH_2_ (**22**). Regarding the interaction of CF*_x_* H*_y_* with the sulfone moiety, only compounds **18** (−SO_2_–CH_2_CF_3_) and **19** (−SO_2_–CF_2_CF_2_H) present weak hydrogen-bond interactions. No interactions with the thioether sulfur were considered due to the low p*K*_HB_ values for the sulfur atom itself. Although the IMHBs may reduce the basicity of the pyridine and thus increase the log*D* ^7.4^ value, the stand-alone effect cannot explain the observed trends in lipophilicity (see Section 12 in Supporting Information for details).

Descriptors comprise constitutional and topological properties of the molecules, DFT-derived parameters such as atomic charges, energies, and molecular orbitals, and the above-mentioned QMPSA and TPSA (Tables S4 and S5). These descriptors contain information about key chemical features widely known to influence lipophilicity, such as molecular volume (total number of atoms, number of fluorine atoms, or DFT-derived molecular volume), molecular polarity (dipole moment of the molecule, QMPSA, or TPSA), and H-bond basicity of the functional groups (atomic charges at pyridinyl nitrogen, or at sulfide and sulfonyl sulfur). Note also that the described descriptors are not necessarily related to a single feature of the compound but can reflect the interplay between different factors considered implicitly. For example, the number of fluorine atoms (#F) is related to the molecular volume, while their inductive effects modulate the polarity of the molecule and H-bond basicity of the functional groups. All descriptors were preprocessed by scaling them between 0 and 1 to ensure that models are not biased by the magnitudes and spread of descriptor values. Then, data were fed into a Python algorithm to systematically generate (multi)­linear regressions between 1 and 2 descriptors and the experimental log*D* ^7.4^ (>24,000 regression models), using three evaluation metrics to select the best model: the regression coefficient (*R* ^2^), the mean absolute error (MAE), and the root-mean-square error (RMSE). The best single-descriptor model was the TPSA regression with only two-descriptor models able to outperform it (see Supporting Information for details), while the DFT-computed log*D* underperformed it (Table S8). This indicates that more than one molecular feature must be evaluated to describe lipophilicity accurately. The selected high-regression metrics (*R* ^2^ = 0.95, MAE = 0.17, RMSE = 0.04) correspond to the following descriptors: (i) the number of fluorine atoms, defined as #F and (ii) the inverse of the calculated Mulliken charge at the sulfur atom, defined by the term 1/*q*_s_. According to the optimal model (Figure 3B), a greater number of fluorine atoms would lead to an increase in the log*D* ^7.4^ value. This is rationalized by an increase in the hydrophobic surface of the molecule (molecular volume) upon substitution of C–H bonds by C–F bonds, and a decrease of the H-bond basicity of pyridine and sulfonyl functional groups due to the inductive effect of fluorine atoms.^44^ The reduced basicity at the functional groups is reflected in the depletion of negative charge at the pyridinyl nitrogen and the sulfonyl oxygens upon fluorination (from *q*_N_ = −0.17 *e* in **5** to *q*_N_ = −0.11 *e* in **4** and *q*_O_ (average *q*_O_) = −0.64 *e* in **15** to *q*_O_ = −0.57 *e* in **17**). On the other hand, the model contains an inverse contribution of Mulliken charge on sulfur (1/*q*_s_), which implies that a buildup of positive charge would lead to a decrease in log*D* ^7.4^ value. Polarization of the sulfur atom by the strongly electron-withdrawing fluorine atoms will increase the partial positive charge on sulfur, leading to an overall dipole moment that reduces its lipophilicity. Thus, the degree of fluorination has opposing effects on lipophilicity: it increases the hydrophobic surface and decreases the H-bond basicity of pyridine and sulfonyl groups while also increasing the overall polarity of the molecule. This latter effect is particularly pronounced when the C–F bonds are adjacent to the highly polarizable sulfur atom. Other two-descriptor models also show good regression metrics (*R* ^2^ > 0.94). Among them, we highlight the following: (1) the absolute free energy in water of the compound Δ*G*_wat_ and the log­(*q*_S_) descriptors and (2) the #F and the inverse of the square of the HOMO energy, 1/*E*_HOMO_^2^. However, note that Δ*G*_wat_ and descriptors 1/*E*_HOMO_^2^ correlate with #F and 1/*q*_S_, respectively, indicating that these other models point toward the same chemical features.

Figure 3C shows a comparison of log*D* ^7.4^ values of pyridines **4**–**7** together with the contribution of the two variables of our computational model (#F and 1/*q*_s_). Difluorinated thioether chains exhibit a significant dependence on whether the fluorines are located terminally or internally (adjacent to S). For internal difluorinated compound **4**, the greater partial positive charge on sulfur (*q*_s_ = +0.18) coincides with a much lower experimental log*D* ^7.4^ value (1.82), while the terminal difluorothioethyl group **6**, which exhibits less polarized sulfur (*q*_s_ = +0.14), has a higher log*D* ^7.4^ of 2.26. Although the combination of internal difluorination with terminal difluorination (tetrafluorinated compound **7**) leads to significant charge on sulfur (*q*_s_ = +0.27), this does not lead to a change in the log*D* ^7.4^ value with respect to the nonfluorinated analogue **5** due to a balance between the polarization of the sulfur by the fluorine atoms and the greater hydrophobic surface imparted by them. Additionally, the lipophilicity of compound **7** might also be influenced by the strength of the IMHB between the terminal −CF_2_H and the pyridyl nitrogen (see Section 12 in Supporting Information for details). Even though this interaction would increase the affinity of the compound for the octanol phase, the polarization of the molecule counterbalances this effect.

For the sulfonyl analogues, we observed the opposite trend, and the difluorinated compound **17** shows higher lipophilicity than the nonfluorinated compound **15** (Figure 2B), following the conventional order. In this case, we identified two key consequences of the H/F exchange on lipophilicity enhancement: (1) the increase of the hydrophobic surface (molecular volume) and (2) the reduction of H-bond basicity of the sulfonic oxygens (average *q*_O_ = −0.64 and −0.57 *e* for **15** and **17**, respectively). In fact, for the sulfone series, we found a fair linear correlation (*r* ^2^ = 0.86) between the less negative Mulliken charge on oxygen and the larger experimental log*D* ^7.4^. Contrary to thioethers, for the sulfone series, the reduction of the H-bond basicity caused by inductive effects of fluorination surmounts the increase of polarization. Our model is able to capture these effects because in the polarized S=O bonds, the charge at the oxygen and the sulfur is inversely correlated. For specific series in our data set, we found similar lipophilicity patterns to those reported for fluorinated alkoxy groups.^45,46^ For methyl thioethers (**1**–**3**) and methyl sulfones (**10**–**13**), there is an exponential log*D* ^7.4^ profile upon fluorination, while for the analogous ethyl sulfones (**14**–**16** and **18**), we observe a parabolic log*D* ^7.4^ profile, highlighting the differences between internal and terminal fluorination (Figure S2). While the investigation of log*D* ^7.4^ with phenyl-substituted analogues of **1**, **3**–**5**, and **8** has been performed,^12^ the inclusion of the pyridyl moiety has a significant impact on lipophilicity (see Section 13 in Supporting Information for comparison).

The computational model can also be employed to predict the lipophilicity of experimentally undetermined compounds such as pyridines **21** and **22** (Figure 4). The log*D* ^7.4^ values of these compounds could not be determined because they decomposed in aqueous media during the measurement period.

**Figure 4:**
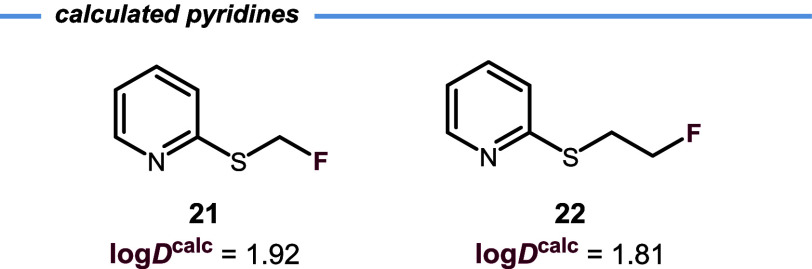
Calculated log*D* values for water-unstable pyridines **21** and **22**.

### Acid–Base Properties

We next turned our attention to evaluating the basicity of 2-(thiofluoroalkyl)­pyridines **1**–**9** and **21**–**22**. While changes in the lipophilic properties of a molecule can modulate its bioavailability, disruptions in its acid–base properties can lead to altered distribution in the body.^47^ Since the thiofluoroalkyl model system has an adjacent pyridine unit, we decided to leverage its basicity to investigate the effect of fluorination on p*K*_a_ changes.

The measurements were conducted on the corresponding conjugate acids of the thiofluoroalkylpyridines using an ^19^F NMR method developed by Leito and coworkers.^48^ For nonfluorinated analogues, p*K*_a_ was determined by an analogous method using ^1^H NMR.^49^ It was found that the replacement of any C–H bond within the thioethyl fragment with a C–F bond resulted in an increase in the acidity of the conjugate acid, with fluorination adjacent to the sulfur atom having a stronger effect than terminal substitution (Figure 5). For example, the replacement of the CH_3_ group with CF_3_ in both the methyl **1** (p*K*_a_ 3.69) and ethyl **5** (p*K*_a_ 3.68) series resulted in a decrease of p*K*_a_ to 0.97 (**3**) and 1.49 (**8**), respectively. Likewise, internal difluorination of the thioethyl group led to a more acidic pyridinium (**9**, p*K*_a_ 2.05) compared to terminal difluorination (**6**, p*K*_a_ 2.43). The introduction of a single fluorine atom in the methyl series lowered the p*K*_a_ to 2.43 (**21**), while this effect is less pronounced for the terminally monofluorinated ethyl motif (**22**, p*K*_a_ = 3.08). Computationally, it is straightforward to determine p*K*_a_ values for molecular organic systems, obtaining quantitative predictions via fitting to experimental data (Table S12).

**Figure 5:**
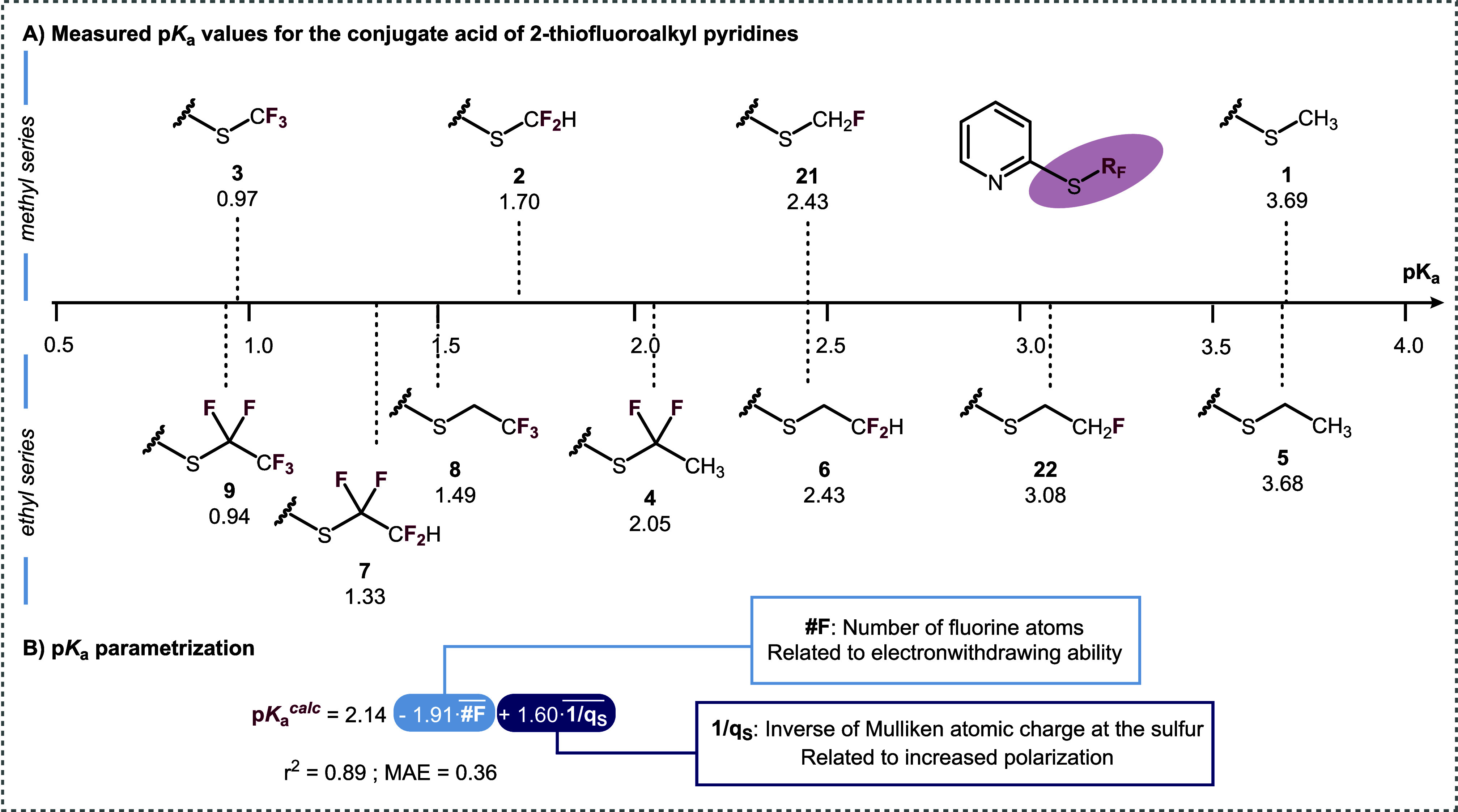
Measured p*K*_a_ values for a range of 2-(thiofluoroalkyl)­pyridines.

We considered that a regression model could be constructed with the chemically meaningful descriptors employed above for lipophilicity modeling, #F and #1/*q*_s_. Using the measured p*K*_a_’s of the protonated thioalkylpyridines in Figure 6, we found a fair correlation between the two descriptors and the experimental p*K*_a_ (*R* ^2^ = 0.89).

**Figure 6:**
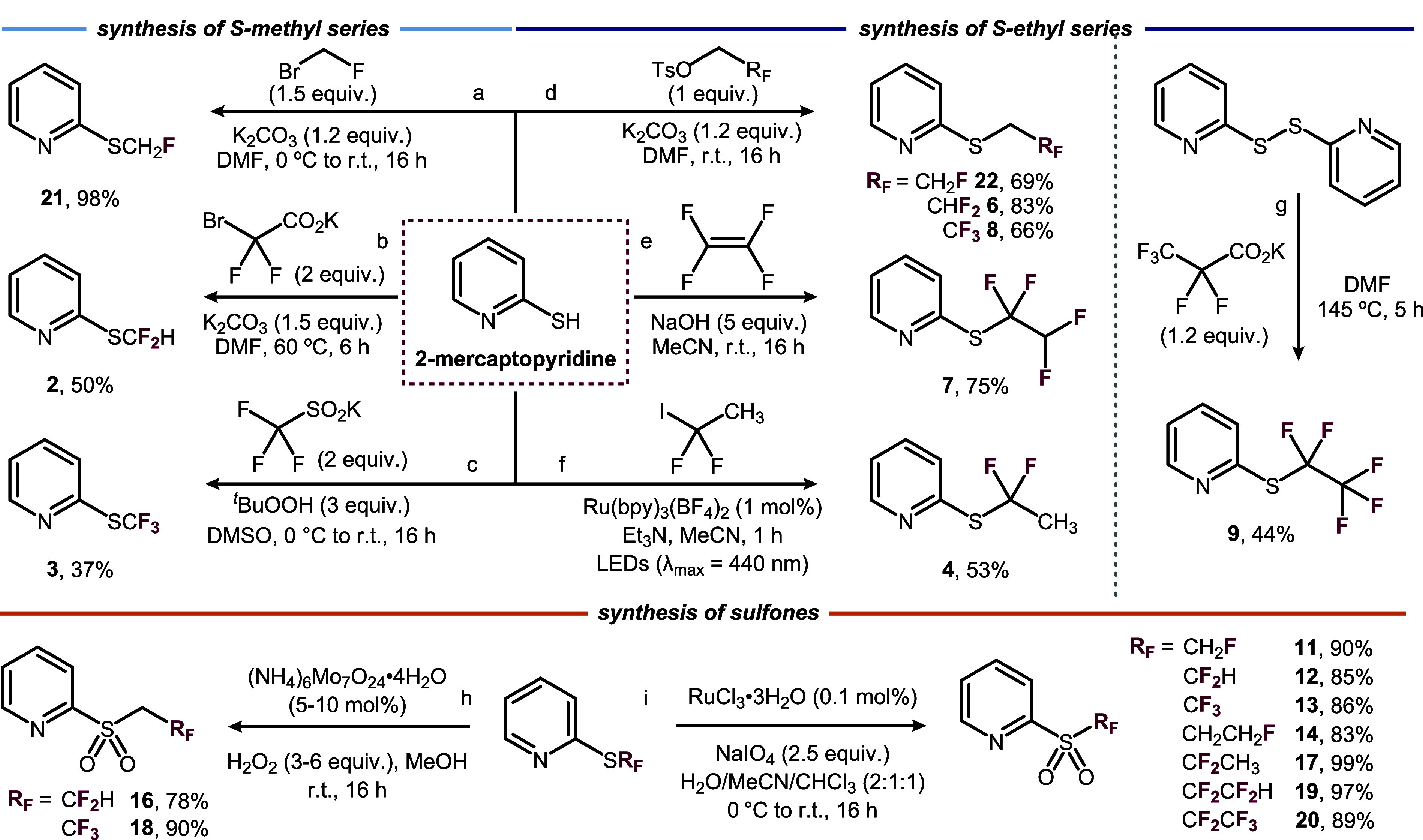
Synthesis of the model 2-thiosubstituted pyridines bearing different fluorinated methyl and ethyl fragments and their oxidized counterparts. All yields are isolated yields. See the Experimental Section for a detailed description of the reaction conditions.

While better correlations were obtained using other suitable descriptors (such as atomic charge at nitrogen), we aimed to compare lipophilicity and acidity models using the same descriptors. The resulting model is similar to that found for lipophilicity (Figure 3), but the coefficient for the #F variable becomes negative. Therefore, both a greater number of fluorine atoms and a greater partial positive charge at the sulfur atom would lead to a decrease in p*K*_a_ of the conjugate acid (pyridinium). While the degree of fluorination and polarization of the sulfur have opposing effects toward overall molecular lipophilicity, they both contribute toward greater acidity.

While simultaneously evaluating both log*D* ^7.4^ and p*K*_a_ values in the pyridine series, certain complementary properties become evident. For example, the basicity of 2-thioethylpyridine **5** can be suppressed through either terminal difluorination or tetrafluoroethylation of the fragment, without affecting its lipophilicity (compound **5** vs **6** and **7**). Conversely, difluorination at an internal position of the ethyl chain can result in a simultaneous decrease in both the basicity and lipophilicity of the molecule (compound **5** vs **4**). Collectively, this information can be harnessed to more precisely fine-tune the molecular properties of compounds featuring thiofluoroalkyl motifs.

### Synthesis

The synthesis of 2-(methylthio)­pyridine fluorinated derivatives was carried out from 2-mercaptopyridine in one step. 2-((Fluoromethyl)­thio)­pyridine **21** was obtained in excellent yield (98%) using bromofluoromethane as an electrophile (Figure 6a), while trapping of mercaptopyridine with *in situ*-generated difluorocarbene gave 2-((difluoromethyl)­thio)­pyridine **2** (50%, Figure 6b). The trifluoromethyl analogue **3** was prepared by reaction with potassium trifluoromethylsulfinate under oxidative conditions (37%, Figure 6c). Terminally mono- (**22**), di- (**6**), and trifluorinated (**8**) ethylthiopyridines were prepared via alkylation of 2-mercaptopyridine with the corresponding fluoroalkyl tosylate in good yields (Figure 6d). To prepare 1,1,2,2-((tetrafluoroethyl)­thio)­pyridine **7**, gaseous tetrafluoroethylene was used under basic conditions to alkylate mercaptopyridine in a good yield (75%, Figure 6e). Initially, the synthesis of internally difluorinated ethylthiopyridine **4** was achieved through a 4-step sequence involving alkylation with bromodifluoroethyl acetate, DIBAL-H reduction, formation of an ethyl dithiocarbonate, and finally a tin-mediated Barton–McCombie deoxygenation in an overall yield of 5% (Schemes S1 and S2 for details and further synthetic attempts). This process was later superseded by a one-step alkylation of mercaptopyridine with iododifluoroethane, accomplished under photoredox conditions with Ru­(bpy)_3_(BF_4_)_2_ as a catalyst and visible-light irradiation after only 1 h (53%, Figure 6f).^50^ Perfluorinated analogue **9** was synthesized by reaction of 2-pyridyl disulfide with perfluoroethyl anions generated via the thermal decarboxylation of potassium pentafluoropropionate (Figure 6g). The preparation of the fluorinated pyridyl sulfones was achieved by oxidation of the corresponding thioethers. Sulfones **10**–**20** can be synthesized with excellent yields (78–99%) using molybdenum or ruthenium catalysis and either hydrogen peroxide or sodium periodate as terminal oxidants (Figure 6h,i).

## Conclusions

A family of 2-thiofluoroalkyl (SR_F_) and 2-sulfonyl fluoroalkyl (SO_2_R_F_) substituted pyridines with methyl and ethyl fragments was prepared. Their lipophilicity (log*D* ^7.4^) and acid–base properties (p*K*_a_) were experimentally determined by using ^19^F NMR methods. The fluorination pattern and degree in the R_F_ chains significantly influenced lipophilicity, sometimes leading to counterintuitive values. To better understand the log*D* ^7.4^ trends, a computational multilinear model using statistical analysis was developed. An excellent correlation between experimental and predicted values was found when the number of fluorine atoms and the inverse of the Mulliken charge at the sulfur atom were used as descriptors. Introducing fluorine atoms into a thioalkyl chain can enhance or decrease the parent molecule’s lipophilicity. Fluorination increases the hydrophobic surface and at the α-position decreases the hydrogen-bond basicity of the functional groups, favoring higher log*D* ^7.4^, but also impacts the polarization of the sulfur atom, having the opposite effect. By controlling the number of fluorine atoms, their disposition in the R_F_ chain, and the oxidation state of the sulfur atom, a small-step scale in log*D* ^7.4^ can be established. Similarly, the acid–base properties of the pyridines were computationally modeled, revealing that the p*K*_a_ values are significantly influenced by the inductive effects of fluorination. Finally, we demonstrated that the influence of fluorination is highly context-dependent and should not be overlooked. Our findings are expected to be useful for the synthetic community, especially in medicinal chemistry, to fine-tune the physicochemical properties of new active pharmaceutical ingredients.

## Experimental Section

### General Remarks

Proton (^1^H NMR), carbon (^13^C­{^1^H} NMR), and fluorine (^19^F NMR) nuclear magnetic resonance spectra were recorded on a Varian Mercury spectrometer or a Bruker Avance Ultrashield (400 MHz for ^1^H), (100.6 MHz for ^13^C­{^1^H}), and (376.5 MHz for ^19^F). All chemical shifts are quoted on the δ scale in parts per million (ppm) using the residual solvent as an internal standard (^1^H NMR: CDCl_3_ = 7.26, CD_3_OD = 3.31 and (^13^C­{^1^H} NMR): CDCl_3_ = 77.16, CD_3_OD = 49.0). Coupling constants (*J*) are reported in Hz with the following splitting abbreviations: s = singlet, d = doublet, *t* = triplet, q = quartet, and app = apparent. High-resolution mass spectra (HRMS) were recorded on an LC–MS system (UHPLC 1290 Infinity II Series coupled to a qTOF/MS 6550 Series, both Agilent Technologies). For ionization, an ESI operating on positive or negative ionization or an APCI operating on positive or negative ionization was used. Water and methanol with 0.05% formic acid were used as mobile phases. The quadrupole time-of-flight mass spectrometer (qTOF) operated in high-resolution MS scan mode between 100 and 1000 *m*/*z*. For GC–HRMS mass determination, the compounds were directly analyzed by gas chromatography coupled to high-resolution mass spectrometry (7200 GC-qTOF from Agilent Technologies). For ionization, electron impact ionization was used. The chromatographic column was a 5HP-MS from Agilent, and the carrier gas was He. The quadrupole time-of-flight mass spectrometer (qTOF) operated in high-resolution MS scan mode between 100 and 600 *m*/*z*. Nominal and exact *m*/*z* values are reported in Daltons. Thin-layer chromatography (TLC) was carried out using commercial backed sheets coated with 60 ÅM F_254_ silica gel. Visualization of the silica plates was achieved using a UV lamp (λ_max_ = 254 nm), 6% H_2_SO_4_ in EtOH, cerium molybdate, and/or potassium permanganate staining solutions. Flash column chromatography was carried out using silica gel 60 Å CC (230–400 mesh). Mobile phases are reported in relative composition (e.g., 1:1 EtOAc/hexane v/v). All reactions using anhydrous conditions were performed using an oven-dried apparatus under an atmosphere of argon. Brine refers to a saturated solution of sodium chloride. Anhydrous sodium sulfate (Na_2_SO_4_) was used as a drying agent after reaction workup, as indicated. All reagents were purchased from Melius Organics, Sigma-Aldrich, Cymit, Carbosynth, Apollo Scientific, Fluorochem, and Manchester Organics chemical companies. Fluoroethyl tosylates were prepared according to reported procedures.^51^ All compounds synthesized for the purposes of this manuscript were characterized by ^1^H, ^13^C, and ^19^F NMR, and high-resolution mass spectrometry, and determined to be >95% pure by RP-HPLC-UV.

### General Procedure for the Introduction of CH_2_R_F_ Motifs (GP-1)

A round-bottom flask, equipped with a magnetic stir bar, was charged with 2-mercaptopyridine (1 equiv) and potassium carbonate (1.2 equiv). The flask was then evacuated and backfilled with argon three times. Subsequently, anhydrous DMF (1 M) was added using a syringe, and the mixture was sparged with argon for 15 min. Next, the corresponding fluoroalkyl tosylate (1 equiv) was added, and the reaction mixture was stirred overnight at room temperature. Then, the mixture was diluted with diethyl ether, washed with brine, and dried over Na_2_SO_4_. Upon filtration, the organic layer was concentrated under reduced pressure and purified by column chromatography.

### General Procedure for the Oxidation of SR_F_ Motifs with Mo-Catalyst (GP-2)

To a solution of pyridine-SR_F_ (1.0 equiv) in MeOH (0.2 M) were added ammonium molybdate tetrahydrate (from 5 to 10%) and hydrogen peroxide (30% (w/w) in water, from 3 to 6 equiv). The reaction was then left to stir overnight at room temperature. Next, the reaction was quenched with water, extracted with Et_2_O, and washed with brine. The combined organic fractions were dried with Na_2_SO_4_, filtered, and evaporated under reduced pressure. Upon filtration, the organic layer was concentrated under reduced pressure and purified (if needed) by column chromatography to render the oxidized products.

### General Procedure for the Oxidation of SR_F_ Motifs with Ru-Catalyst (GP-3)

To a solution of pyridine-SR_F_ (1.0 equiv) in H_2_O (1 mL), CH_3_CN (0.5 mL), and CHCl_3_ (0.5 mL) were added ruthenium­(III) chloride trihydrate and sodium periodate at 0 °C. The reaction was then left to stir overnight at room temperature. Next, the reaction was quenched with water, extracted with dichloromethane, and washed with brine. The combined organic fractions were dried with Na_2_SO_4_, filtered, and evaporated under reduced pressure. Upon filtration, the organic layer was concentrated under reduced pressure and purified (if needed) by column chromatography to render the oxidized products.

#### 2-((Difluoromethyl)­thio)­pyridine (**2**)

A 50 mL round-bottom flask, equipped with a magnetic stir bar, was charged with dried potassium bromodifluoroacetate (7.93 g, 37.2 mmol, 2.0 equiv), 2-mercaptopyridine (2.07 g, 18.6 mmol, 1.0 equiv), and potassium carbonate (3.86 g, 27.9 mmol, 1.5 equiv). The flask was then evacuated and backfilled with argon three times. Subsequently, anhydrous DMF (11 mL, 1.7 M) was added by using a syringe. Then, the reaction mixture was stirred for 6 h at 60 °C. The mixture was diluted with diethyl ether, washed with brine, and dried over Na_2_SO_4_. Upon filtration, the organic layer was concentrated under reduced pressure and purified by flash column chromatography (SiO_2_, 1:9 ethyl acetate/hexane) to afford **2** as a pale-yellow oil (1.51 g, 50%). ^1^H NMR (CDCl_3_, 400 MHz): δ 8.48 (d, *J* = 4.8, 1H), 7.68 (t, *J* = 56.2 Hz, 1H), 7.59 (t, *J* = 7.7 Hz, 1H), 7.25 (d, *J* = 7.9, 1H), 7.13 (dd, *J* = 7.4, 4.9 Hz, 1H); ^13^C NMR (CDCl_3_, 100.6 MHz): δ 153.2, 150.1, 137.1, 124.3, 121.7, 121.4 (t, *J* = 270.9 Hz); ^19^F NMR (CDCl_3_, 376.5 MHz): δ −96.28 (d, *J* = 56.1 Hz, 2F); HRMS (ESI^+^) for (M + H)^+^ C_6_H_6_F_2_NS^+^ (*m*/*z*): calcd 162.0184; found 162.0188.

#### 2-((Trifluoromethyl)­thio)­pyridine (**3**)

In a 250 mL round-bottom flask equipped with a magnetic stir bar, 2-mercaptopyridine (1 g, 9.0 mmol, 1.0 equiv) and potassium trifluoromethanesulfinate (2.8 g, 18.0 mmol, 2.0 equiv) were added and dissolved in DMSO (90 mL, 0.1 M). Then, the solution was cooled to 0 °C, and *tert*-butyl hydroperoxide (70% (w/w) in water, 3.8 mL, 27.0 mmol, 3.0 equiv) was added dropwise. The mixture was stirred overnight at room temperature. To the reaction mixture, water was added, and it was extracted with CH_2_Cl_2_. The combined organic layers were washed with brine, dried over Na_2_SO_4_, filtered, and concentrated under reduced pressure. The organic residue was purified by flash column chromatography (SiO_2_, 2:8 ethyl acetate/hexane) to afford **3** (597 mg, 37%) as a pale-yellow oil. ^1^H NMR (CDCl_3_, 400 MHz): δ 8.56 (d, *J* = 4.9 Hz, 1H), 7.66 (t, *J* = 7.7 Hz, 1H), 7.52 (d, *J* = 7.9 Hz, 1H), 7.25 (dd, *J* = 7.5, 4.8 Hz, 1H); ^13^C NMR (CDCl_3_, 100.6 MHz): δ 150.7, 149.4, 137.7, 129.4 (q, *J* = 307.6 Hz), 128.2, 123.8; ^19^F NMR (CDCl_3_, 376.5 MHz): δ −40.21 (s, 3F); HRMS (ESI^+^) for (M + H)^+^ C_6_H_5_F_3_NS^+^ (*m*/*z*): calcd 180.0089; found 180.0092.

#### 2-((1,1-Difluoroethyl)­thio)­pyridine (**4**)

Following a reported procedure,^39^ in an 8 mL vial, Ru­(bpy)_3_BF_4_ (7.4 mg, 0.01 mmol, 0.01 equiv) and 2-mercaptopyridine (111 mg, 1.0 mmol, 1.0 equiv) were charged. The flask was then evacuated and backfilled with argon three times. Subsequently, anhydrous acetonitrile (4 mL) was added by using a syringe, and the mixture was sparged with argon for 15 min. Next, a solution of triethylamine (153 μL, 1.1 mmol, 1.1 equiv) in acetonitrile (1 mL) was added to the previous solution, followed by 1,1-difluoro-1-iodoethane (768 mg, 4.0 mmol, 4.0 equiv). The solution was irradiated for 1 h at 25 °C using two Kessil 450 nm lamps in a 3D-printed photochemical reactor.^52^ The mixture was then concentrated under reduced pressure and purified using column chromatography (SiO_2_, 90:10 to 85:15 hexane/EtOAc) to afford **4** as a pale-yellow oil (187 mg, 53%). ^1^H NMR (CDCl_3_, 400 MHz): δ 8.54 (ddd, *J* = 4.8, 1.8, 0.8 Hz, 1H), 7.64 (td, *J* = 7.6, 1.9 Hz, 1H), 7.58 (d, *J* = 7.9 Hz, 1H), 7.20 (ddd, *J* = 7.3, 4.9, 1.3 Hz, 1H), 2.04 (t, *J* = 17.1 Hz, 2H); ^13^C NMR (CDCl_3_, 100.6 MHz): δ 152.5, 150.3, 137.2, 128.7 (t, *J* = 276.6 Hz), 128.2 (t, *J* = 2.2 Hz), 122.9, 26.8 (t, *J* = 25.6 Hz); ^19^F NMR (CDCl_3_, 376.5 MHz): δ −65.29 (q, *J* = 17.1 Hz); HRMS (ESI^+^) for (M + H)^+^ C_7_H_6_F_4_NO_2_S^+^ (*m*/*z*): calc. 244.0050; found 244.0055.

#### 2-(Ethylthio)­pyridine (**5**)

A 25 mL round-bottom flask equipped with a magnetic stir bar was charged with 2-mercaptopyridine (700 mg, 6.3 mmol, 1.0 equiv) and potassium carbonate (1.04 g, 7.6 mmol, 1.2 equiv). The flask was then evacuated and backfilled with argon three times. Subsequently, anhydrous DMF (7 mL, 0.9 M) was added by using a syringe, and the mixture was sparged with argon for 15 min. Next, ethyl iodide (0.5 mL, 6.3 mmol, 1.0 equiv) was added, and the reaction mixture was stirred overnight at room temperature. The crude product was then diluted with diethyl ether, washed with brine, and dried over Na_2_SO_4_. Upon filtration, the organic layer was concentrated under reduced pressure to afford pure **5** (763 mg, 87%) as a colorless oil. The spectroscopic data are in agreement with those reported in the literature.^53^ ^1^H NMR (CDCl_3_, 400 MHz): δ 8.41 (ddd, *J* = 5.0, 1.9, 1.0 Hz, 1H), 7.48–7.41 (m, 1H), 7.14 (dt, *J* = 8.1, 1.0 Hz, 1H), 6.98–6.90 (m, 1H), 3.15 (q, *J* = 7.4 Hz, 2H), 1.36 (t, *J* = 7.4 Hz, 3H); ^13^C NMR (CDCl_3_, 100.6 MHz): δ 159.5, 149.5, 135.9, 122.2, 119.3, 24.5, 14.7.

#### 2-((2,2-Difluoroethyl)­thio)­pyridine (**6**)

Following the general procedure GP-1, starting from K_2_CO_3_ (946 mg, 6.8 mmol), 2-mercaptopyridine (634 mg, 5.7 mmol), 2,2-difluoroethyl tosylate (1.35 g, 5.7 mmol), and DMF (5.7 mL), the pyridine derivative **6** (844 mg, 83%) was obtained as a black oil after purification by flash column chromatography (SiO_2_, 1:9 ethyl acetate/hexane). ^1^H NMR (CDCl_3_, 400 MHz): δ 8.40 (d, *J* = 4.7 Hz, 1H), 7.49 (t, *J* = 7.8 Hz, 1H), 7.19 (d, *J* = 8.1 Hz, 1H), 7.01 (dd, *J* = 7.4, 4.9 Hz, 1H), 6.00 (tt, *J* = 57.1, 4.6 Hz, 1H), 3.58 (td, *J* = 15.0, 4.6 Hz, 2H); ^13^C NMR (CDCl_3_, 100.6 MHz): δ 156.3, 149.5, 136.3, 122.2, 120.1, 115.2 (t, *J* = 241.7 Hz); ^19^F NMR (CDCl_3_, 376.5 MHz): δ −115.18 (dt, *J* = 57.2, 15.5 Hz, 2F); HRMS (ESI^+^) for (M + H)^+^ C_7_H_8_F_2_NS^+^ (*m*/*z*): calc. 176.0340; found 176.0342.

#### 2-((1,1,2,2-Tetrafluoroethyl)­thio)­pyridine (**7**)

A 50 mL round-bottom flask equipped with a magnetic stir bar was charged with potassium hydroxide (1.32 g, 23.5 mmol, 5.0 equiv) and 2-mercaptopyridine (526 mg, 4.7 mmol, 1.0 equiv). The flask was then evacuated and backfilled with argon three times. Subsequently, anhydrous acetonitrile (12 mL, 0.4 M) was added by using a syringe. Then, a current of tetrafluoroethylene was bubbled into the solution for 15 min, and the mixture was stirred overnight at room temperature. Tetrafluoroethylene was generated *ex situ* through the reaction of TMSCF_3_ with NaI in acetonitrile.^54^ The mixture was then acidified with HCl, diluted with diethyl ether, washed with brine, and dried over Na_2_SO_4_. Upon filtration, the organic layer was concentrated under reduced pressure and purified by flash column chromatography (SiO_2_, 1:9 ethyl acetate/hexane) to afford **7** (759 mg, 75%) as a pale-yellow oil. ^1^H NMR (CDCl_3_, 400 MHz): δ 8.55 (d, *J* = 4.6 Hz, 1H), 7.68 (t, *J* = 7.6 Hz, 1H), 7.51 (d, *J* = 7.9 Hz, 1H), 7.30–7.22 (m, 1H), 6.33 (tt, *J* = 53.8, 4.6 Hz, 1H); ^13^C NMR (CDCl_3_, 100.6 MHz): δ 150.5, 149.7 (t, *J* = 4.1 Hz), 137.5, 128.2, 123.5, 122.5 (tt, *J* = 286.6, 28.5 Hz), 109.5 (tt, *J* = 253.9, 34.6 Hz); ^19^F NMR (CDCl_3_, 376.5 MHz): δ −93.65 (m, 2F), −134.75 (dt, *J* = 53.8, 9.7 Hz, 2F); HRMS (ESI^+^) for (M + H)^+^ C_7_H_6_F_4_NS^+^ (*m*/*z*): calc. 212.0152; found 212.0155.

#### 2-((2,2,2-Trifluoroethyl)­thio)­pyridine (**8**)

Following the general procedure GP-1, starting from K_2_CO_3_ (859 mg, 6.2 mmol), 2-mercaptopyridine (575 mg, 5.2 mmol), 2,2,2-trifluoroethyl tosylate (1.32 g, 5.2 mmol), and DMF (5.2 mL), the pyridine derivative **8** (672 mg, 66%) was obtained as a pale yellow oil after purification by flash column chromatography (SiO_2_, 1:9 ethyl acetate/hexane). ^1^H NMR (CDCl_3_, 400 MHz): δ 8.43 (d, *J* = 4.9 Hz, 1H), 7.51 (t, *J* = 7.8 Hz, 1H), 7.22 (d, *J* = 7.9 Hz, 1H), 7.04 (dd, *J* = 7.4, 5.0 Hz, 1H), 4.03 (q, *J* = 9.9 Hz, 2H); ^13^C NMR (CDCl_3_, 100.6 MHz): δ 154.6, 149.4, 136.5, 125.5 (q, *J* = 275.8 Hz), 122.4, 120.5, 30.97 (q, *J* = 33.6 Hz); ^19^F NMR (CDCl_3_, 376.5 MHz): δ −66.65 (t, *J* = 10.2 Hz, 3F); HRMS (ESI^+^) for (M + H)^+^ C_7_H_7_F_3_NS^+^ (*m*/*z*): calcd 194.0246; found 194.0251.

#### 2-((Perfluoroethyl)­thio)­pyridine (**9**)

A 25 mL round-bottom flask, equipped with a magnetic stir bar, was charged with dried potassium perfluoroacetate (1.01 g, 5.3 mmol, 1.2 equiv) and 2,2′-dipyridyldisulfide (0.87 g, 4.4 mmol, 1.0 equiv). The flask was then evacuated and backfilled with argon three times. Subsequently, anhydrous DMF (11 mL, 0.4 M) was added by using a syringe. Then, the reaction mixture was stirred for 5 h at 145 °C. The mixture was diluted with diethyl ether, washed with brine, and dried over Na_2_SO_4_. Upon filtration, the organic layer was concentrated under reduced pressure and purified by flash column chromatography (SiO_2_, 1:9 ethyl acetate/hexane) to afford **9** (672 mg, 44%) as a yellow oil. ^1^H NMR (CDCl_3_, 400 MHz): δ 8.63 (d, *J* = 4.8 Hz, 1H), 7.73 (t, *J* = 7.7 Hz, 1H), 7.65 (d, *J* = 7.9 Hz, 1H), 7.39–7.31 (m, 1H); ^13^C NMR (CDCl_3_, 100.6 MHz): δ 150.9, 147.6 (t, *J* = 2.9 Hz), 137.6, 130.8, 124.5, 120.6 (tq, *J* = 289.6, 41.0 Hz), 118.5 (qt, *J* = 286.5, 36.4 Hz); ^19^F NMR (CDCl_3_, 376.5 MHz): δ −82.86 (t, *J* = 3.5 Hz, 3F), −90.66 (m, 2F); HRMS (ESI^+^) for (M + H)^+^ C_7_H_5_F_5_NS^+^ (*m*/*z*): calc. 230.0057; found 230.0064.

#### 2-(Methylsulfonyl)­pyridine (**10**)

Following the general procedure GP-2, starting from pyridine **1** (225 mg, 1.8 mmol), ammonium molybdate tetrahydrate (111 mg, 0.09 mmol), H_2_O_2_ (551 μL, 4.7 mmol), and MeOH (9 mL), the pyridine derivative **10** (221 mg, 78%) was obtained as a yellowish solid after workup. The spectroscopic data are in agreement with those reported in the literature.^55^ ^1^H NMR (CDCl_3_, 400 MHz): δ 8.72 (ddd, *J* = 4.7, 1.5, 0.9 Hz, 1H), 8.07 (dt, *J* = 7.9, 1.0 Hz, 1H), 7.96 (td, *J* = 7.8, 1.7 Hz, 1H), 7.55 (ddd, *J* = 7.6, 4.7, 1.2 Hz, 1H), 3.21 (s, 3H); ^13^C NMR (CDCl_3_, 100.6 MHz): δ 157.9, 150.1, 138.4, 127.5, 121.1, 40.1.

#### 2-((Fluoromethyl)­sulfonyl)­pyridine (**11**)

Following the general procedure GP-3, starting from pyridine **21** (143 mg, 1 mmol), RuCl_3_·3H_2_O (2 mg), and NaIO_4_ (535 mg, 2.5 mmol), the pyridine derivative **11** (158 mg, 90%) was obtained as a pale-yellow solid. The spectroscopic data are in agreement with those reported in the literature.^56^ ^1^H NMR (CDCl_3_, 400 MHz): δ 8.78 (ddd, *J* = 4.7, 1.6, 0.9 Hz, 1H), 8.15 (dt, *J* = 7.8, 1.0 Hz, 1H), 8.02 (td, *J* = 7.8, 1.7 Hz, 1H), 7.62 (ddd, *J* = 7.7, 4.7, 1.1 Hz, 1H), 5.52 (d, *J* = 46.9 Hz, 2H); ^13^C NMR (CDCl_3_, 100.6 MHz): δ 154.1, 150.6, 138.5, 128.3, 123.7, 88.7 (d, *J* = 219.0 Hz); ^19^F NMR (CDCl_3_, 376.5 MHz): δ −213.6 (t, *J* = 46.9 Hz).

#### 2-((Difluoromethyl)­sulfonyl)­pyridine (**12**)

Following the general procedure GP-3, starting from pyridine **2** (300 mg, 1.9 mmol), RuCl_3_·3H_2_O (1 mg), and NaIO_4_ (995 mg, 4.7 mmol), the pyridine derivative **12** (316 mg, 85%) was obtained as a white solid after purification by flash column chromatography (SiO_2_, 4:6 ethyl acetate/hexane). ^1^H NMR (CDCl_3_, 400 MHz): δ 8.82 (d, *J* = 4.4 Hz, 1H), 8.15 (d, *J* = 7.9 Hz, 1H), 8.04 (t, *J* = 7.8 Hz, 1H), 7.71–7.64 (m, 1H), 6.62 (t, *J* = 53.4 Hz, 1H); ^13^C NMR (CDCl_3_, 100.6 MHz): δ 152.7, 151.0, 138.7, 128.9, 125.1, 114.0 (t, *J* = 286.3 Hz); ^19^F NMR (CDCl_3_, 376.5 MHz): δ −124.45 (d, *J* = 53.6 Hz, 2F); HRMS (ESI^+^) for (M + H)^+^ C_6_H_6_F_2_NO_2_S^+^ (*m*/*z*): calc. 194.0082; found 194.0088.

#### 2-((Trifluoromethyl)­sulfonyl)­pyridine (**13**)

Following the general procedure GP-3, pyridine **3** (300 mg, 1.7 mmol), RuCl_3_·3H_2_O (1 mg), and NaIO_4_ (920 mg, 4.3 mmol), the pyridine derivative **13** (304 mg, 86%) was obtained as a white solid after purification by flash column chromatography (SiO_2_, 4:6 ethyl acetate/hexane). ^1^H NMR (CDCl_3_, 400 MHz): δ 8.90 (d, *J* = 4.5 Hz, 1H), 8.23 (d, *J* = 7.9 Hz, 1H), 8.08 (t, *J* = 7.8 Hz, 1H), 7.73 (dd, *J* = 7.8, 4.7 Hz, 1H); ^13^C NMR (CDCl_3_, 100.6 MHz): δ 151.4, 138.7, 129.5, 126.3, 119.8 (q, *J* = 326.9 Hz); ^19^F NMR (CDCl_3_, 376.5 MHz): δ −75.60 (s, 3F); HRMS (ESI^+^) for (M + H)^+^ C_6_H_5_F_3_NO_2_S^+^ (*m*/*z*): calcd 211.9988; found 211.9986.

#### 2-((2-Fluoroethyl)­sulfonyl)­pyridine (**14**)

Following the general procedure GP-3, starting from pyridine **22** (300 mg, 1.9 mmol), RuCl_3_·3H_2_O (1 mg), and NaIO_4_ (1.02 g, 4.8 mmol), the pyridine derivative **14** (309 mg, 83%) was obtained as a yellow solid after purification by flash column chromatography (SiO_2_, 4:6 ethyl acetate/hexane). ^1^H NMR (CDCl_3_, 400 MHz): δ 8.74 (d, *J* = 4.7 Hz, 1H), 8.09 (d, *J* = 7.8 Hz, 1H), 7.97 (t, *J* = 7.8 Hz, 1H), 7.56 (dd, *J* = 7.7, 4.7 Hz, 1H), 4.85 (dt, J = 46.4, 5.7 Hz, 2H), 3.82 (dt, *J* = 21.9, 5.7 Hz, 2H); ^13^C NMR (CDCl_3_, 100.6 MHz): δ 157.4, 150.2, 138.3, 127.6, 122.0, 77.0 (d, *J* = 172.7 Hz), 52.5 (d, *J* = 21.8 Hz); ^19^F NMR (CDCl_3_, 376.5 MHz): δ −221.35 (tt, *J* = 46.5, 21.9 Hz, 1F); HRMS (ESI^+^) for (M + H)^+^ C_7_H_9_FNO_2_S^+^ (*m*/*z*): calc. 190.0333; found 190.0341.

#### 2-(Ethylsulfonyl)­pyridine (**15**)

Following the general procedure GP-2, starting from pyridine **5** (250 mg, 1.8 mmol), ammonium molybdate tetrahydrate (111 mg, 0.09 mmol), H_2_O_2_ (551 μL, 4.7 mmol), and MeOH (9 mL), the pyridine derivative **15** (277 mg, 90%) was obtained as a yellowish solid after workup. The spectroscopic data are in agreement with those reported in the literature.^57^ ^1^H NMR (CDCl_3_, 400 MHz): δ 8.85–8.69 (m, 1H), 8.08 (d, *J* = 7.8 Hz, 1H), 7.96 (td, *J* = 7.8, 1.6 Hz, 1H), 7.55 (dd, *J* = 7.1, 4.9 Hz, 1H), 3.41 (q, *J* = 7.5 Hz, 2H), 1.28 (t, *J* = 7.5 Hz, 3H); ^13^C NMR (CDCl_3_, 100.6 MHz): δ 156.5, 150.3, 138.2, 127.5, 122.4, 46.4, 6.8.

#### 2-((2,2-Difluoroethyl)­sulfonyl)­pyridine (**16**)

Following the general procedure GP-2, starting from pyridine **6** (300 mg, 1.7 mmol), ammonium molybdate tetrahydrate (105 mg, 0.09 mmol), H_2_O_2_ (525 μL, 5.1 mmol), and MeOH (5.2 mL), the pyridine derivative **16** (308 mg, 78%) was obtained as a white solid after purification by flash column chromatography (SiO_2_, 4:6 ethyl acetate/hexane). *R*_*f*_: (4:6 ethyl acetate/hexane): 0.33; ^1^H NMR (CDCl_3_, 400 MHz): δ 8.76 (d, *J* = 4.6 Hz, 1H), 8.08 (d, *J* = 7.9 Hz, 1H), 7.99 (t, *J* = 7.8 Hz, 1H), 7.59 (dd, *J* = 7.7, 4.7 Hz, 1H), 6.26 (tt, *J* = 54.8, 4.6 Hz, 1H), 4.00 (td, *J* = 13.6, 4.6 Hz, 2H); ^13^C NMR (CDCl_3_, 100.6 MHz): δ 156.8, 150.4, 138.5, 128.0, 122.0, 111.7 (t, *J* = 243.6 Hz), 55.0 (t, *J* = 25.1 Hz); ^19^F NMR (CDCl_3_, 376.5 MHz): δ −115.11 (dt, *J* = 55.0, 13.7 Hz, 2F); HRMS (ESI^+^) for (M + H)^+^ C_7_H_8_F_2_NO_2_S^+^ (*m*/*z*): calc. 208.0238; found 208.0247.

#### 2-((1,1-Difluoroethyl)­sulfonyl)­pyridine (**17**)

Following the general procedure GP-3, starting from pyridine **4** (15 mg, 0.09 mmol), RuCl_3_·3H_2_O (0.3 mg), and NaIO_4_ (45.8 mg, 0.214 mmol), the pyridine derivative **17** (18 mg, 99%) was obtained as a yellow solid after workup. The spectroscopic data are consistent with those reported previously.^58^ ^1^H NMR (CDCl_3_, 400 MHz): δ 9.00–8.75 (m, 1H), 8.18 (d, *J* = 7.9 Hz, 1H), 8.04 (td, *J* = 7.8, 1.7 Hz, 1H), 7.67 (ddd, *J* = 7.7, 4.7, 1.1 Hz, 1H), 2.12 (t, *J* = 18.7 Hz, 3H); ^13^C NMR (CDCl_3_, 100.6 MHz): δ 152.3, 151.1, 138.4, 128.8, 126.5, 124.7 (t, *J* = 284.7 Hz), 17.7 (t, *J* = 21.5 Hz); ^19^F NMR (CDCl_3_, 376.5 MHz): δ −95.6 (q, *J* = 18.6 Hz).

#### 2-((2,2,2-Trifluoroethyl)­sulfonyl)­pyridine (**18**)

Following the general procedure GP-2, starting from pyridine **8** (300 mg, 1.6 mmol), ammonium molybdate tetrahydrate (96 mg, 0.08 mmol), H_2_O_2_ (476 μL, 4.7 mmol), and MeOH (5.2 mL), the pyridine derivative **18** (329 mg, 90%) was obtained as a white solid after purification by flash column chromatography (SiO_2_, 4:6 ethyl acetate/hexane). ^1^H NMR (CDCl_3_, 400 MHz): δ 8.76 (d, *J* = 4.4 Hz, 1H), 8.12 (d, *J* = 7.9 Hz, 1H), 8.01 (t, *J* = 7.8 Hz, 1H), 7.65–7.57 (m, 1H), 4.31 (qt, *J* = 9.0, 1.1, 2H); ^13^C NMR (CDCl_3_, 100.6 MHz): δ 156.3, 150.4, 138.6, 128.2, 122.2, 121.2 (q, *J* = 276.9 Hz), 53.4 (q, *J* = 31.8 Hz); ^19^F NMR (CDCl_3_, 376.5 MHz): δ −61.11 (t, *J* = 9.0 Hz, 3F); HRMS (ESI^+^) for (M + H)^+^ C_7_H_7_F_3_NO_2_S^+^ (*m*/*z*): calc. 226.0144; found 226.0159.

#### 2-((1,1,2,2-Tetrafluoroethyl)­sulfonyl)­pyridine (**19**)

Following the general procedure GP-3, starting from pyridine **7** (300 mg, 1.4 mmol), RuCl_3_·3H_2_O (1 mg), and NaIO_4_ (760 mg, 3.5 mmol), the pyridine derivative **19** (339 mg, 97%) was obtained as a white solid after purification by flash column chromatography (SiO_2_, 4:6 ethyl acetate/hexane). ^1^H NMR (CDCl_3_, 400 MHz): δ 8.90–8.84 (m, 1H), 8.19 (d, *J* = 7.8 Hz, 1H), 8.08 (t, *J* = 7.6 Hz, 1H), 7.76–7.68 (m, 1H), 6.39 (tt, *J* = 52.1, 5.8 Hz, 1H); ^13^C NMR (CDCl_3_, 100.6 MHz): δ 151.8, 151.2, 138.9, 129.6, 126.3, 115.1 (tt, *J* = 298.2, 27.0 Hz), 107.8 (tt, *J* = 256.3, 28.3 Hz); ^19^F NMR (CDCl_3_, 376.5 MHz): δ −119.20 (m, 2F), −135.01 (dt, *J* = 52.1, 8.3 Hz, 2F); HRMS (ESI^+^) for (M + H)^+^ C_7_H_6_F_4_NO_2_S^+^ (*m*/*z*): calc. 244.0050; found 244.0055.

#### 2-((Perfluoroethyl)­sulfonyl)­pyridine (**20**)

Following the general procedure GP-3, starting from pyridine **9** (300 mg, 1.3 mmol), RuCl_3_·3H_2_O (1 mg), and NaIO_4_ (700 mg, 3.3 mmol), the pyridine derivative **20** (308 mg, 89%) was obtained as a white solid after purification by flash column chromatography (SiO_2_, 4:6 ethyl acetate/hexane). ^1^H NMR (CDCl_3_, 400 MHz): δ 8.90 (d, *J* = 4.8 Hz, 1H), 8.22 (d, *J* = 7.9 Hz, 1H), 8.09 (t, *J* = 7.8 Hz, 1H), 7.77–7.70 (m, 1H); ^13^C NMR (CDCl_3_, 100.6 MHz): δ 151.6, 151.4, 138.7, 129.6, 126.8, 116.0 (m), 113.1 (m); ^19^F NMR (CDCl_3_, 376.5 MHz): δ −78.08 (s, 3F), −114.80 (s, 2F); HRMS (ESI^+^) for (M + H)^+^ C_7_H_5_F_5_NO_2_S^+^ (*m*/*z*): calc. 261.9956; found 261.9962.

#### 2-((Fluoromethyl)­thio)­pyridine (**21**)

A 10 mL round-bottom flask equipped with a magnetic stir bar was charged with 2-mercaptopyridine (556 mg, 5 mmol, 1.0 equiv) and potassium carbonate (830 mg, 6 mmol, 1.2 equiv). The flask was then evacuated and backfilled with argon three times. Subsequently, anhydrous DMF (5 mL, 1 M) was added by using a syringe. Then, bromofluoromethane (∼850 mg, 7.5 mmol, 1.5 equiv) was bubbled into the reaction mixture at 0 °C, and the reaction was stirred overnight at room temperature with a water bath. The crude product was diluted with diethyl ether, washed with brine, and dried over Na_2_SO_4_. Upon filtration, the organic layer was concentrated under reduced pressure to afford pure **21** (1.00 g, 98%) as a yellow oil. ^1^H NMR (CDCl_3_, 400 MHz): δ 8.51 (ddd, *J* = 4.9, 1.8, 0.9 Hz, 1H), 7.58 (td, *J* = 7.8, 1.9 Hz, 1H), 7.28 (dt, *J* = 8.0, 0.9 Hz, 1H), 7.10 (ddd, *J* = 7.4, 4.9, 1.0 Hz, 1H), 6.15 (d, *J* = 51.7 Hz, 2H); ^13^C NMR (CDCl_3_, 100.6 MHz): δ 155.3, 149.9, 136.9, 122.9 (d, *J* = 1.9 Hz), 121.1, 83.5 (d, *J* = 215.9 Hz); ^19^F NMR (CDCl_3_, 376.5 MHz): δ −187.53 (t, *J* = 51.7 Hz, 1F); HRMS (ESI^+^) for (M+H)^+^ C_6_H_7_FNS^+^ (*m*/*z*): calc. 144.0278; found 144.0278.

### General Procedure for Log *D* ^7.4^ Determination Using ^19^F NMR

A protocol developed by the group of Linclau^33^ was followed for the determination of the log *D* ^7.4^ values of the 2-substituted pyridines. The 4-step process was replicated three times for each compound:

**Partitioning:** to a 10 mL pear-shaped flask were added 2 mL of 1-octanol, 2-substituted pyridine (1–10 mg), trifluoroethanol (5 μL), and 2 mL of phosphate buffer (pH 7.4). The resulting mixture was stirred at 25 °C for 2 h by controlling the temperature by an immersion cooler, and then, it was left to stand at 25 °C overnight to enable complete phase separation.
**Sample preparation:** using a 1 mL disposable syringe, an aliquot of 0.6 mL was carefully taken from the aqueous or the 1-octanol phase. Next, the needle was carefully wiped with a dry tissue, and the aliquot was placed into an NMR tube, followed by addition of 0.1 mL of acetone-*d*_6_. The NMR tubes were sealed using a rubber septum stopper and shaken to obtain a homogeneous solution for NMR measurement. When taking an aliquot of the lower water phase, to avoid the contamination of the syringe with the upper octanol phase, 0.05 mL of air was taken into the syringe before putting the needle into the solution, and while immersing it through the upper octanol layer, the air was gently pushed out. Upon reaching the water phase, all air bubbles are pushed out of the syringe, the aliquot is taken, and the needle is quickly removed from the solution. Then, a small amount of the water phase was discarded to ensure all traces of octanol are out of the needle, leaving a 0.6 mL sample in the syringe.
**NMR measurement:** fluorine (^19^F­{1H} NMR) nuclear magnetic resonance spectra were recorded on a Varian Mercury spectrometer or Bruker Avance Ultrashield (376.5 MHz for ^19^F NMR). Parameters used in the determination of lipophilicities were obtained from the experiments carried out by Linclau and coworkers.^33^ First, the sealed tube was inserted to the NMR spectrometer, and following automatic locking and gradient shimming, a simple ^19^F spectrum was recorded on the nonspinning sample to assess the required spectral width (SW) and frequency offset point (O1P). The O1P is centered between the two diagnostic F signals, and the spectral width (SW) is left at 200 ppm (it can be reduced if a better S/N ratio is required). Then, the 90° pulse was measured with the automated pulsecal routine obtaining a power (PW) of 16 μs. The measured 90° pulse, SW, and O1P were transferred into the inversion–recovery experiment. For practical purposes, it is recommended to use a D1 of 30 s for the octanol sample and of 60 s for the water sample as pulse delay, given the D1 value should be greater than 5*T1 for quantitative integration. The number of transients (NS) is selected for each sample to afford a suitable signal-to-noise ratio (SNR should be >250).
**Data processing:**data were processed using Mestre Nova NMR software. The obtained FID file was reprocessed using the following conditions: WFunction (LB = 2, exponential), zero filling (increasing points from 65,536 to 262,144) and then Fourier transform, followed by phasing with mouse and auto baseline correction. The integration ratio was obtained by manual integration.

### General Procedure for Log *D* ^7.4^ Determination Using HPLC-UV

A protocol described by Ràfols and coworkers^59^ was followed for the determination of the log *D* ^7.4^ values of the nonfluorinated pyridines. The 4-step process was replicated three times for each compound:

**Stocksolution preparation:** in a 12 mL vial, 15 mg of the pyridine was charged. Then, 6 mL of phosphate buffer at pH 7.4 was added, and the mixture was vigorously shaken for 2 h. An aliquot of this stock solution was measured by HPLC-UV.
**Partitioning:** three partitioning experiments were carried out for each compound. To prepare an experiment, in a vial containing a magnetic stir bar, a known amount of the stock solution was introduced, followed by another known amount of 1-octanol. Next, the biphasic mixture was stirred for 2 h and left to stand overnight to allow phase partition. For the selection of the different volume ratios, it was followed reported recommendations,^59^ although 1:1, 10:1, and 1:10 octanol:stock solutions were usually employed.
**Sample preparation and measurement:** to prepare the sample, 0.1 mL of the aqueous phase was taken and introduced in a 2 mL vial, following the precautions described in the previous section. The samples were then analyzed with the same HPLC-UV method as that of the stock solution. The log *D* ^7.4^ value was obtaining by comparing the initial amount of pyridine in the aqueous phase before and after partitioning, taking into consideration the volume rates.

### General Procedure for p*K*_a_ Determination Using NMR

A protocol developed by the group of Leito^48^ was followed for the determination of the log *D* ^7.4^ values of fluorinated 2-substituted pyridines using ^19^F NMR. For the nonfluorinated ones, a similar protocol developed by the group of Gift^49^ using ^1^H NMR was followed.

**Aqueous solutions:** 11 aqueous stock solutions (10 mL) with different p*K*_a_ values (from −1 to 12) were prepared using HCl, NaOH, Na_2_CO_3_, and Milli-Q water.
Sample preparation: in an NMR tube, a small amount of the pyridine of interest was introduced (∼1 mg), followed by 0.5 mL of a given pH aqueous solution. Next, the tube was caped and shaken vigorously, and the pH of the resulting solution was measured inside the tube with a pH meter with an NMR-tube probe. This procedure was repeated for all of the aqueous solutions to have a battery of NMR tubes at different pH values. If needed, additional solutions were prepared to ensure a continuum of pH values.
**Sample measurement:** for ^19^F NMR measurements, a 3 mm NMR tube containing a solution of 8 mg of KF in 1 mL of D_2_O was inserted into the NMR tube of interest to serve as an external standard. Next, a standard ^19^F NMR experiment was recorded to determine the chemical shifts of the external standard and the sample of interest. For the ^1^H method, the same protocol was applied by changing the external standard for 5 μL of trifluoroethanol instead of KF. The battery of NMR samples at different pH values was measured.
**Data processing:** using MestReNova NMR software, the chemical shifts of the pyridines were determined. For ^19^F NMR experiments, the KF reference was set to −125.00 ppm, and the closest ^19^F signal of the pyridine was chosen for the chemical shift determination. For ^1^H NMR experiments, the trifluoroethanol reference (CF_3_CH_2_OH) was set to 3.14 ppm, and the methyl signals of the nonfluorinated pyridines were chosen for the chemical shift determination. Next, the selected signal for each pyridine was plotted against the pH of the measured solution. Using Prism GraphPad software, a sigmoidal curve was adjusted, and the second derivate was performed to obtain the p*K*_a_ value of the given pyridine.

### Computational Details

Density functional theory (DFT) calculations were performed with the Gaussian 16 software,^60^ using the B3LYP functional^61^ with Grimme’s GD3-BJ dispersion correction to enhance accuracy^62^ and the 6-31+G** basis set for all atoms.^63^ Gibbs free energies were computed at 298.15 K. Solvent effects of water and *n*-octanol were included using the implicit solvation model IEF-PCM.^64^ A conformational analysis of all studied substrates was done using CREST (Conformer–Rotamer Ensemble Sampling Tool)^65^ that uses the semiempirical method GFN*n*-xTB^66^ and automatically performs a conformational sampling based on metadynamics simulations. Then, the lowest energy conformers obtained with CREST (2 to 6 structures) were reoptimized at the DFT level described above. The details of descriptor calculations and statistical tools are provided in Supporting Information.

## Supplementary Material





## Notes

Data for compound characterization, log*D* ^7.4^ and p*K*_a_ raw numeric data, details of the statistical modeling, and the Cartesian coordinates of the computed structures (PDF)
Molecular formula strings (CSV)

## Abbreviations

#F: number of fluorine atoms
DFT: density functional theory
DMF: *N*,*N*-dimethylformamide
DMSO: dimethyl sulfoxide
*e*: unitary atomic unit of charge
IMHB: intramolecular hydrogen bonding
MAE: mean average error
NCI: non-covalent interactions
QMPSA: quantum mechanics polar surface area
*q*_s_: Mulliken charge at the sulfur atom
R_F_: fluoroalkyl chain
r.t.: room temperature
*t*Bu: *tert*-butyl
TPSA: topological polar surface area
Ts: tosyl
