# Supplementary material for: Impact of Fluorine Pattern on Lipophilicity and Acid–Base Properties of 2‑(Thiofluoroalkyl)pyridines: Insights from Experiments and Statistical Modeling
Source: J Med Chem. 2025 Feb 17;68(4):4787–800. doi: 10.1021/acs.jmedchem.4c03045 (PMC12124721; doi:10.1021/acs.jmedchem.4c03045)
Supplement: Supplementary file 1 [file jm4c03045_si_001.pdf]

Supporting Information

Impact of Fluorine Pattern on Lipophilicity and Acid-Base Properties of 2-(Thiofluoroalkyl)pyridines: Insights from Experiments and Statistical Modeling

Miguel Bernús,[†,‡] Gonzalo D. Núñez,[§,‡] Will C. Hartley,[†] Marc Guasch,[†] Jordi Mestre,[†] Maria Besora,[*,§] Jorge J. Carbó,[*,§] and Omar Boutureira[*,†]

[†]*Departament de Química Analítica i Química Orgànica, Universitat Rovira i Virgili, 43007 Tarragona, Spain*

[§]*Departament de Química Física i Inorgànica, Universitat Rovira i Virgili, 43007 Tarragona, Spain*

[‡]*Equal contribution*

**E-mail(s): omar.boutureira@urv.cat, maria.besora@urv.cat, j.carbo@urv.cat*

Table of Contents

1. Synthetic attempts towards compound **4** ... 3
2. $\log D^{7.4}$ measurements data ... 6
3. $\log D^{7.4}$ HPLC-UV measurements data ... 7
4. pK_a NMR measurements data ... 8
5. Additional computational details ... 9
6. Workflow of the computational study ... 9
7. Descriptors used in the multivariate data analysis ... 10
8. Data sets used for the statistical analysis ... 17
9. Statistical analysis ... 18
   9.1. Regression process ... 18
   9.2. Code used for statistical analysis ... 19
10. Regression model selection ... 19
   10.1. Selected model overview ... 19
   10.2. Response variable and descriptors values for the model ... 20
   10.3. The model results ... 21
   10.4. Descriptor's weight. Key to understand the model ... 22
11. Other Tested Models ... 23
   11.1. Model 2: TPSA ... 23
   11.2. Model 3: QMPSA ... 24
   11.3. Model 4: Traditional DFT-based log*D* prediction ... 24
   11.4. Energy-weighted descriptors values for the different isomers ... 26
12. Relevance of hydrogen bonds in the studied substrates ... 27
13. Comparison of lipophilicity with literature precedents ... 31
14. pK_a predictions ... 32
15. Cartesian coordinates (in Angstroom) and potential energies (in Hartree) of the optimized structures. ... 34
16. NMR spectra ... 39
17. Purity of compounds **1**, **5**, **10**, and **15** ... 74
18. References ... 77

1. Synthetic attempts towards compound 4

The synthesis of pyridine **4** proved challenging, with up to six different strategies pursued without success (Scheme S1). Firstly, the 2-thioalkylnyl pyridine **int-1** was hypothesized as a precursor to **4**. Use of TIPS-EBX reagent resulted in decomposition (Scheme S1, A.1). Nucleophilic alkynylation (Scheme S1, A.2 and A.3) was also unsuccessful. Attempts to methylate pyridine **2** resulted in only decomposition products when THF was used as a solvent, or byproduct **bp-1** when DMF was used (Scheme S1, B). Electrophilic sources of the fluoroalkyl fragment were used to alkylate 2-pyridyl thiol (Scheme S1, C and D), but only starting material was recovered after multiple attempts. Finally, a strategy using radical addition to the disulfide also failed to deliver the targeted pyridine **4** (Scheme S1, E).

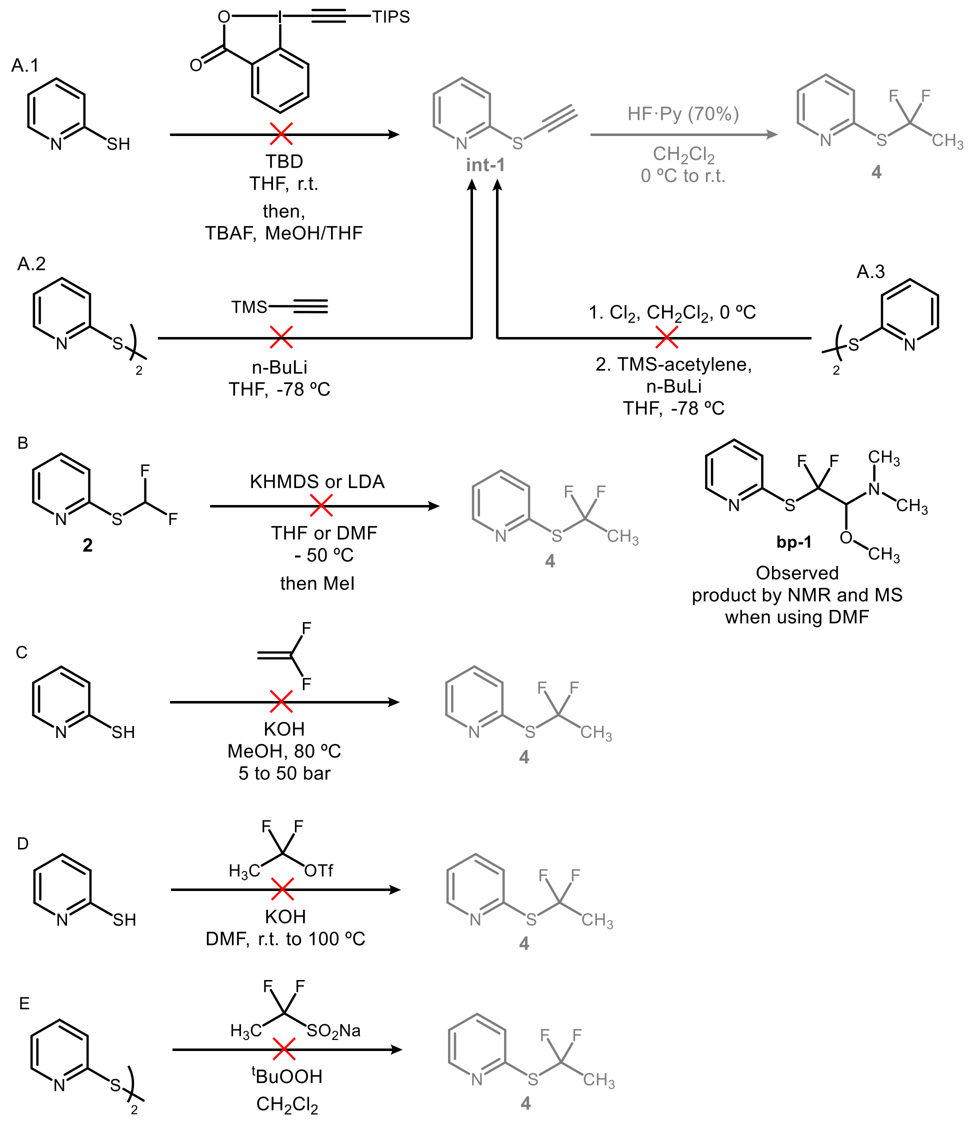

Scheme S1. Synthetic attempts towards compound **4**

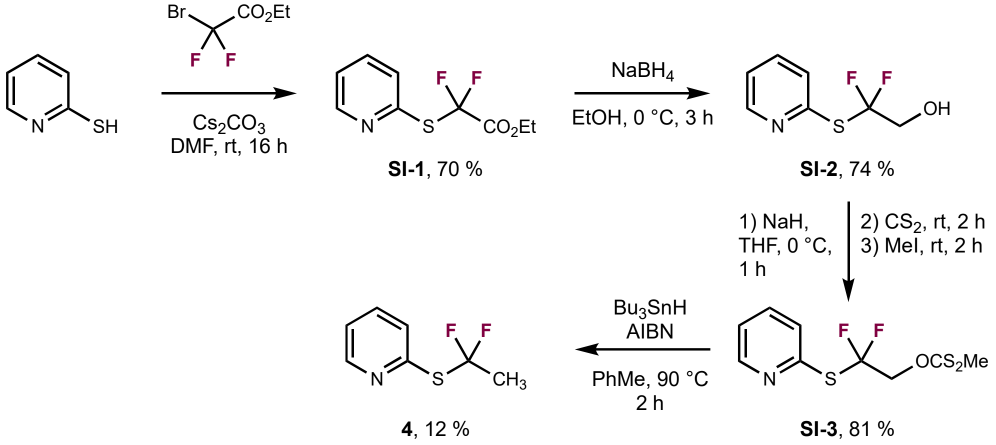

Scheme S2. First successful route towards pyridine **4**

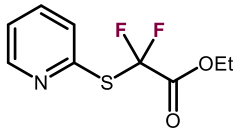

Ethyl 2,2-difluoro-2-(pyridin-2-ylthio)acetate (S1). A 100 mL round-bottom flask, equipped with a magnetic stir bar, was charged with 2-mercaptopyridine (1.67 g, 15 mmol, 1.0 equiv.) and cesium carbonate (9.77 g, 30 mmol, 2.0 equiv.). The flask was then evacuated and backfilled with argon three times. Subsequently, anhydrous DMF (50 mL, 0.3 M) was added using a syringe, and the mixture was sparged with argon for 15 min. Next, ethyl bromodifluoroacetate (2.89 mL, 22.5 mmol, 1.5 equiv.) was added and the reaction mixture was stirred overnight at room temperature. The mixture was diluted with diethyl ether, washed with brine several times, and dried over anhydrous Na_2SO_4. Upon filtration, the organic layer was concentrated under reduced pressure, and purified by column chromatography (SiO_2, 1:9 ethyl acetate/hexane) to afford **S1** as a yellow oil (2.45 g, 70%). **^{1}H NMR** ($CDCl_3$, 400 MHz): δ 8.51 (ddd, J = 4.9, 1.9, 0.9 Hz, 1H), 7.66 (td, J = 7.7, 1.9 Hz, 1H), 7.50 (dd, J = 7.9, 1.0 Hz, 1H), 7.37–7.19 (m, 1H), 4.32 (q, J = 7.1 Hz, 2H), 1.28 (td, J = 7.2, 0.8 Hz, 3H). **^{13}C NMR** ($CDCl_3$, 101 MHz): δ 161.8 (t, J = 31.7 Hz), 151.1, 150.3, 137.4, 127.6, 123.3, 123.2, 119.0 (t, J = 286.1 Hz), 63.8, 13.9. **^{19}F NMR** ($CDCl_3$, 377 MHz): δ –82.3. (s, 1F). **HRMS** (ESI^+) for $(M+Na)^+$ $C_9H_9F_2NNaO_2S^+$ (*m/z*): calc. 256.0214; found 256.0212.

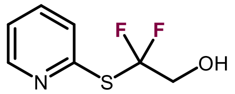

2,2-Difluoro-2-(pyridin-2-ylthio)ethan-1-ol (S2). A 50 mL round-bottom flask, equipped with a magnetic stir bar, was charged with ethyl 2,2-difluoro-2-(pyridin-2-ylthio)acetate **S1,** (2.45 g, 10.5 mmol, 1.0 equiv.). The flask was then evacuated and backfilled with argon three times. Subsequently, anhydrous ethanol (25 mL, 0.42 M) was added using a syringe. Next, sodium borohydride (776 mg, 20.5 mmol, 2.0 equiv.) was added at 0 ºC, and the reaction mixture was stirred for 3 h in an ice bath. The mixture was diluted with dichloromethane, washed with brine, and dried over anhydrous Na_2SO_4. Upon filtration, the organic layer was concentrated under reduced pressure, and purified by column chromatography (SiO_2, from 5:95 to 20:80 ethyl acetate/hexane) to afford **S2** as a yellow solid (1.49 g, 74%). **^{1}H NMR** ($CDCl_3$, 400 MHz): δ 8.54 (ddd, J = 4.9, 2.0, 0.9 Hz, 1H), 7.71 (td, J = 7.7, 1.9 Hz, 1H), 7.59 (dt, J = 7.9, 1.3 Hz, 1H), 7.30 (ddd, J = 7.5, 4.9, 1.3 Hz, 1H), 4.84 (bs, 1H), 3.98 (t, J = 12.1 Hz, 2H). **^{13}C NMR** ($CDCl_3$, 101 MHz): δ 151.0 (t, J = 4.6 Hz), 150.1, 138.0, 129.7 (t, J = 282.6 Hz), 129.2, 123.6, 65.4 (t, J = 31.2 Hz). **^{19}F NMR** ($CDCl_3$, 377 MHz): δ –80.23 (t, J = 11.9 Hz). **HRMS** (ESI^+) for $(M+H)^+$ $C_7H_8F_2NOS^+$ (*m/z*): calc. 192.0289; found 192.0287.

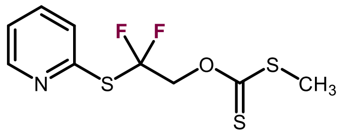

***O*-(2,2-Difluoro-2-(pyridin-2-ylthio)ethyl) *S*-methyl carbonodithioate (S3).** A 10 mL round-bottom flask, equipped with a magnetic stir bar, was charged with 2,2-difluoro-2-(pyridin-2-ylthio)ethan-1-ol **S2** (382 mg, 2 mmol, 1.0 equiv.). The flask was then evacuated and backfilled with argon three times. Subsequently, anhydrous THF (5 mL, 0.40 M) was added using a syringe. Next, sodium hydride (60% wt. dispersion in mineral oil, 200 mg, 5 mmol, 2.5 equiv.) was added at 0 ºC, and the reaction stirred for 1 h at room temperature. The mixture was cooled to 0 ºC, and carbon disulfide (604 µL, 10 mmol, 5.0 equiv.) was added via syringe, and the mixture was stirred for 2 h at room temperature. The mixture was cooled to 0 ºC and methyl iodide (250 µL, 4 mmol, 2.0 equiv.) added via syringe, and stirred for 2 h at room temperature. The reaction was quenched with aqueous NH_4Cl, diluted with dichloromethane, washed with brine, and dried over anhydrous Na_2SO_4. Upon filtration, the organic layer was concentrated under reduced pressure, and purified by column chromatography (SiO_2, 1:9 ethyl acetate/hexane) to afford **S3** as a red oil (453 mg, 81%). **^{1}H NMR** ($CDCl_3$, 400 MHz): δ 8.58 (dd, *J* = 4.8, 1.1 Hz, 1H), 7.67 (td, *J* = 7.7, 1.9 Hz, 1H), 7.55 (d, *J* = 7.9 Hz, 1H), 7.40–7.22 (m, 1H), 5.18 (t, *J* = 12.5 Hz, 2H), 2.59 (s, 3H). **^{13}C NMR** ($CDCl_3$, 101 MHz): δ 214.8, 151.1 (t, *J* = 3.9 Hz), 150.6, 137.5, 128.4, 127.5 (t, *J* = 281.7 Hz), 123.4, 72.2 (t, *J* = 29.5 Hz), 19.4. **^{19}F NMR** ($CDCl_3$, 377MHz): δ –80.15 (t, J = 12.6 Hz). **HRMS** (ESI^+) for $(M+H)^+$ $C_9H_{10}F_2NOS_3^+$ (*m/z*): calc. 281.9887; found 281.9882.

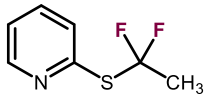

2-((1,1-Difluoroethyl)thio)pyridine (4). A 5 mL round-bottom flask, equipped with a magnetic stir bar, was charged with *O*-(2,2-difluoro-2-(pyridin-2-ylthio)ethyl) *S*-methyl carbonodithioate **S3**, (400 mg, 1.42 mmol, 1.0 equiv.), tributyltin hydride (575 µL, 2.13 mmol, 1.5 equiv.), and azobisisobutyronitrile (AIBN) (35 mg, 0.21 mmol, 0.15 equiv.). The flask was rapidly evacuated and backfilled with argon three times. Anhydrous toluene (3 mL, 0.47 M) was added via syringe, and the reaction mixture was stirred for 2 h at 90 °C. The mixture was concentrated under reduced pressure and purified using preparative thin-layer chromatography (SiO_2, 3:7 Et_2O/pentane) to afford **4** as a pale-yellow oil (30 mg, 12%). **^{1}H NMR** (CD_2Cl_2, 400 MHz): δ 8.68–8.49 (m, 1H), 7.69 (td, *J* = 7.7, 1.9 Hz, 1H), 7.59 (d, *J* = 8.0 Hz, 1H), 7.26 (ddd, *J* = 7.5, 4.8, 1.1 Hz, 1H), 2.08 (t, *J* = 17.3 Hz, 3H). **^{13}C NMR** (CD_2Cl_2, 101 MHz): δ 152.8, 150.6, 137.5, 129.3 (t, *J* = 275.7 Hz), 128.2, 123.2, 27.0 (t, *J* = 25.5 Hz). **^{19}F NMR** (CD_2Cl_2, 377 MHz): δ –65.6 (q, *J* = 17.2 Hz). **HRMS** (ESI^+) for $(M+H)^+$ $C_7H_6F_4NO_2S^+$ (*m/z*): calc. 244.0050; found 244.0055.

2. log$D^{7.4}$ measurements data

Table S1. Raw integration values for the calculation of the log$D^{7.4}$ values using ^{19}F NMR

Compound	n-Octanol		Buffer						Mean	Deviation
	$I_{compound}$	I_{ref}	$I_{compound}$	I_{ref}	ρ_{oct}	ρ_{aq}	D	log$D$	log$D$	σ
**2**	58,74	41,26	3,76	96,24	1,424	0,039	13,12	1,922	1,95	0,03
	57,16	42,84	3,35	96,65	1,334	0,035	13,86	1,945		
	51,64	48,36	2,45	97,55	1,068	0,025	15,31	1,989		
**3**	58,67	41,33	2,38	97,62	1,420	0,024	20,96	2,125	2,13	0,01
	63,61	36,39	2,93	97,07	1,748	0,030	20,85	2,123		
	62,88	37,12	2,75	97,25	1,694	0,028	21,57	2,137		
**4**	1,00	55,14	1,00	1399,72	0,018	0,001	9,14	1,765	1,82	0,21
	1,00	39,16	1,00	1913,57	0,026	0,001	17,59	2,049		
	1,00	53,94	1,00	1012,86	0,019	0,001	6,76	1,634		
**6**	48,92	51,08	1,18	98,82	0,958	0,012	28,87	2,264	2,26	0,01
	50,84	49,16	1,31	98,69	1,034	0,013	28,05	2,252		
	55,57	44,43	1,51	98,49	1,251	0,015	29,37	2,272		
**7**	62,34	37,66	2,16	97,85	1,655	0,022	27,00	2,235	2,27	0,03
	69,57	30,43	2,57	97,44	2,286	0,026	31,21	2,298		
	68,05	31,95	2,53	97,47	2,130	0,026	29,54	2,274		
**8**	63,30	36,70	0,75	99,25	1,725	0,008	82,17	2,718	2,71	0,03
	56,34	43,66	0,55	99,45	1,290	0,006	84,00	2,728		
	58,35	41,65	0,67	99,33	1,401	0,007	74,77	2,677		
**9**	65,79	34,21	0,85	99,15	1,923	0,009	80,76	2,711	2,76	0,05
	68,15	31,85	0,76	99,24	2,140	0,008	100,58	2,806		
	67,83	32,16	0,80	99,20	2,109	0,008	94,15	2,778		
**11**	1,00	5,01	1,00	2,71	0,200	0,369	0,19	0,093	0,10	0,01
	1,00	5,04	1,00	2,85	0,198	0,351	0,20	0,112		
	1,00	5,10	1,00	2,70	0,196	0,370	0,19	0,084		
**12**	44,05	55,95	32,37	67,63	0,787	0,479	0,59	0,576	0,55	0,02
	40,09	59,91	30,63	69,37	0,669	0,442	0,55	0,541		
	44,97	55,03	33,78	62,22	0,817	0,543	0,54	0,538		
**13**	49,82	50,18	7,67	92,33	0,993	0,083	4,30	1,437	1,43	0,00
	50,93	49,07	8,09	91,91	1,038	0,088	4,24	1,432		
	50,98	49,02	8,08	91,92	1,040	0,088	4,26	1,433		
**14**	14,46	85,54	37,99	62,01	0,169	0,613	0,10	-0,199	-0,21	0,06
	15,68	84,32	37,99	62,01	0,186	0,613	0,11	-0,158		
	14,59	85,41	42,38	57,62	0,171	0,736	0,08	-0,274		
**16**	34,57	65,43	37,54	62,46	0,528	0,601	0,32	0,304	0,31	0,01
	34,79	65,21	37,20	62,80	0,534	0,592	0,32	0,315		
	36,82	63,18	39,58	60,42	0,583	0,655	0,32	0,309		
**17**	1,00	3,18	1,00	8,48	0,314	0,118	0,96	0,786	0,79	0,01
	1,00	3,10	1,00	8,22	0,323	0,122	0,95	0,784		
	1,00	3,19	1,00	8,67	0,313	0,115	0,98	0,794		
**18**	53,64	46,36	28,95	71,05	1,157	0,407	1,02	0,813	0,81	0,01
	52,62	47,38	28,62	71,38	1,111	0,401	1,00	0,802		
	56,85	43,15	32,22	67,78	1,317	0,475	1,00	0,803		
**19**	41,79	58,21	5,69	94,31	0,718	0,060	4,28	1,436	1,43	0,00
	57,38	42,62	10,21	89,79	1,346	0,114	4,26	1,433		
	54,84	45,16	9,34	90,65	1,214	0,103	4,24	1,431		
**20**	61,15	38,85	2,33	97,68	1,574	0,024	23,76	2,179	2,21	0,03
	61,73	38,27	2,10	97,90	1,613	0,021	27,07	2,236		
	61,41	38,59	2,20	97,79	1,591	0,022	25,46	2,210		

3. log$D^{7.4}$ HPLC-UV measurements data

Table S2. Raw integration values for the calculation of the log$D^{7.4}$ values using HPLC-UV

Compound	Sample	Intensity	Volume buffer (mL)	Volume n-octanol (mL)	Intensity Ratio	Volume ratio	log*D*	log*D* mean	σ
1	Initial	1626,203						1,69	0,05
	A	36,503	0,5	0,5	43,550	1	1,639		
	B	269,722	1	0,1	5,029	10	1,701		
	C	1055,761	1	0,01	0,540	100	1,733		
5	Initial	1444,289						2,26	0,05
	A	7,672	0,5	0,5	187,256	1	2,272		
	B	85,699	1	0,1	15,853	10	2,200		
	C	480,685	1	0,01	2,005	100	2,302		
10	Initial	1580,581						-0,43	0,03
	A	321,605	0,5	5	3,915	0,1	-0,407		
	B	1140,856	0,5	0,5	0,385	1	-0,414		
	C	1528,718	1	0,1	0,034	10	-0,469		
15	Initial	1446,022						-0,07	0,04
	A	140,648	0,5	5	9,281	0,1	-0,032		
	B	781,317	0,5	0,5	0,851	1	-0,070		
	C	1343,707	1	0,1	0,076	10	-0,118		

4. pK_a NMR measurements data

Table S3. Raw chemical ^{19}F shift data for different pyridines

Compound 1	
pH	^{1}H δ (ppm)
-1	2.59
0.09	2.59
0.31	2.59
1.16	2.6
2.52	2.58
2.86	2.56
3.84	2.45
4.93	2.36
5.88	2.34
6.29	2.35
6.38	2.35
6.54	2.35
6.86	2.35
10.74	2.35

Compound 2	
pH	^{19}F δ (ppm)
-2	-94.01
-0.84	-94.00
0.19	-94.03
0.57	-94.11
1.17	-94.43
1.23	-94.4
1.41	-94.67
1.92	-95.14
2.07	-95.3
2.22	-95.42
2.47	-95.58
2.8	-95.74
3.26	-95.84
3.93	-95.83
5.02	-95.87
5.82	-95.85
7.02	-95.82
7.53	-95.84
9.36	-95.86
11.97	-95.82

Compound 3	
pH	^{19}F δ (ppm)
-2.01	-42.97
-0.96	-42.96
0.06	-42.98
0.47	-43.14
1.19	-43.33
1.51	-43.42
2.04	-43.51
2.32	-43.53
3.21	-43.55
6.55	-43.55
7.31	-43.55
9.92	-43.55
11.37	-43.55

Compound 4	
pH	^{19}F δ (ppm)
-1	-65.34
0.14	-65.46
0.99	-65.62
1.94	-66.3
2.76	-67.1
3.74	-67.45
4.51	-67.48
4.6	-67.5
5.15	-67.5
5.46	-67.5
8.45	-67.48
10.52	-67.5

Compound 5	
pH	^{1}H δ (ppm)
-1	3.14
0.17	3.14
1.08	3.15
2.21	3.14
3.56	3.05
4.39	2.96
5.34	2.93
6.29	2.93
7.41	2.93
10.73	2.92

Compound 6	
pH	^{19}F δ (ppm)
0.45	-119.17
0.67	-119.17
1.05	-119.17
1.3	-119.15
1.51	-119.14
2.14	-118.98
2.37	-118.93
2.7	-118.81
3.42	-118.64
4.09	-118.62
5.03	-118.62
6.59	-118.58
6.99	-118.58
7.08	-118.58
7.37	-118.58
7.68	-118.58
9.36	-118.58
10.81	-118.58
11.78	-118.58

Compound 7	
pH	^{19}F δ (ppm)
-1,98	-91,41
-0,89	-91,43
-0,04	-91,42
0,49	-91,62
0,72	-91,82
1,15	-92,43
1,45	-92,92
1,7	-93,25
1,92	-93,63
2,03	-93,76
2,46	-93,87
3,21	-94,00
3,80	-94,03
4,59	-94,03
5,94	-94,03
6,05	-94,02
6,38	-94,02
7,16	-94,03
11,44	-94,03

Compound 8	
pH	^{19}F δ (ppm)
-1.92	-69.19
-0.87	-69.19
0.19	-69.23
1.91	-69.31
2.25	-69.32
3.1	-69.35
3.63	-69.38
6.48	-69.39
10.25	-69.39
11.36	-69.39

Compound 9	
pH	^{19}F δ (ppm)
-1.78	-93.02
-0.74	-93.05
0.22	-93.11
0.7	-93.34
1.27	-93.62
2.1	-93.87
3.21	-93.93
4.15	-93.93
5.37	-93.92
6.92	-93.9
8.36	-93.9
11.07	-93.9

Compound 22	
pH	^{19}F δ (ppm)
-1	-218.69
0.08	-218.76
0.9	-218.81
0.13	-218.75
1.02	-218.81
2.9	-218.01
3.89	-217.01
4.67	-216.83
5.3	-216.79
6.51	-216.8
7.21	-216.79
7.49	-216.79
9.99	-216.79
10.52	-216.81

Compound 21	
pH	^{19}F δ (ppm)
-1	-190.44
0.14	-190.44
1.05	-190.41
2.09	-189.85
2.98	-188.93
4.1	-188.64
4.5	-188.62
6.44	-188.59
6.65	-188.51
6.9	-188.56
9.19	-188.41
11.41	-188.42

5. Additional computational details

The geometric and electronic structures of compounds were obtained through a combination of a conformational search protocol and DFT calculations, both described in the Computational Details of the main text. Natural Bond Orbital (NBO),[1,2] analysis, version 3.1, were used to obtain some of the descriptors (see Descriptors Used in the Multivariate Data Analysis section of this Supporting Information). NBO calculations were done at the same level of theory as described above for DFT optimizations. Topological polar surface area (TPSA)[3] and Quantum polar surface area (QPSA)[4] descriptors were obtained using Schaftenaar and de Vlieg method and through Molden visualization software[5] (see "Other Tested Models" section for more information).

6. Workflow of the computational study

Scheme S3 shows the workflow used to study substrates **1** to **22**, integrates semi-empirical (GFN*n*-xTB) and Density Functional Theory (DFT) calculations. We started the study optimizing an initial geometry of the substrates at DFT level. Subsequently, conformers of each substrate were systematically searched using CREST (see Computational Details in the main text), leading to the selection of the most stable conformers based on computational predictions at GFN*n*-xTB level. After, the GFN*n*-xTB conformers were re-optimized at DFT level. Descriptors were then generated from the DFT calculations and the chemical structures of the compounds (see further details below at Descriptors Used in the Multivariate Data Analysis section). These descriptors serve as quantitative measures of various molecular properties, enabling further analysis. Mathematical operations were applied to these descriptors, resulting in the derivation of 215 descriptors that collectively characterize the fluorine compounds under study. Statistical techniques were employed to analyze the generated descriptors, revealing patterns, correlations, and trends within the dataset. More than 23,000 regression models were developed based on these descriptors, aiming to establish relationships between molecular characteristics and properties of interest. Regression models with high coefficients of determination (R^2) were selected, to identify meaningful chemical interpretations and valuable insights into the properties of the studied substrates. This integrated workflow allows for a comprehensive investigation of substrates, combining theoretical calculations with statistical data analysis.

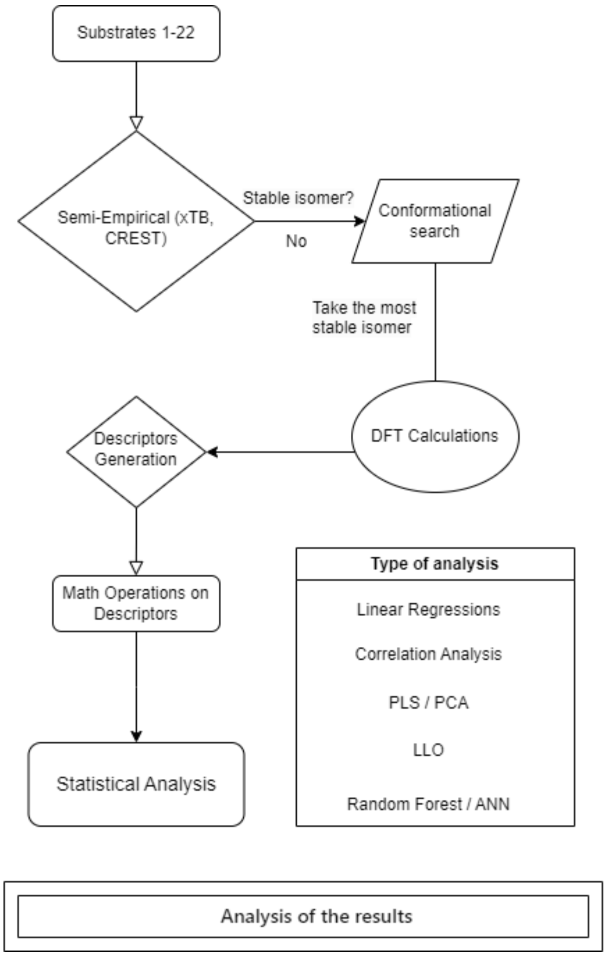

Scheme S3. Representation of the workflow of the computational study

7. Descriptors used in the multivariate data analysis

Descriptors for all studied substrates, **1** to **22**, were obtained by different means. 43 main descriptors, comprising 6 topological descriptors (quantifying molecular structure without spatial details) and 37 Density Functional Theory (DFT)-derived descriptors. For each of these descriptors, we performed the following operations: squaring, taking the logarithm, computing the reciprocal, and obtaining the reciprocal of the square. This resulted in a set of 30 descriptors from chemical structure that is topological/structural descriptors such as the number of atoms or the number of fluorine atoms. A complete list is presented in Table S4. And another set of 185 descriptors was obtained from DFT calculations, descriptors such as HOMO or LUMO energies, atomic charges, etc. a complete list is presented in Table S5.

All descriptors have been pre-treated by performing a normalization to ensure uniformity of numerical magnitudes of the descriptors. Normalization is done by dividing all crude values by the maximum absolute value obtained for that descriptor in all substrates in the training set. With this simple mathematical operation values ranging from 0 to 1 are obtained.

Table S4. Definition of the topological/structural descriptors used in the multivariate data analysis

**1**	D	Number of atoms
**2**	D	Number of Fluorines
**3**	D	Number of Fluorines in alpha
**4**	D	Number of Fluorines in beta
**5**	D	Length of Carbon chain
**6**	D	Number of Hydrogens in the chain
**7**	Log(D)	Number of atoms
**8**	Log(D)	Number of Fluorines
**9**	Log(D)	Number of Fluorines in alpha
**10**	Log(D)	Number of Fluorines in beta
**11**	Log(D)	Length of Carbon chain
**12**	Log(D)	Number of Hydrogens in the chain
**13**	1/D	Number of atoms
**14**	1/D	Number of Fluorines
**15**	1/D	Number of Fluorines in alpha
**16**	1/D	Number of Fluorines in beta
**17**	1/D	Length of Carbon chain
**18**	1/D	Number of Hydrogens in the chain
**19**	D^2	Number of atoms
**20**	D^2	Number of Fluorines
**21**	D^2	Number of Fluorines in alpha
**22**	D^2	Number of Fluorines in beta
**23**	D^2	Length of Carbon chain
**24**	D^2	Number of Hydrogens in the chain
**25**	$1/(D^2)$	Number of atoms
**26**	$1/(D^2)$	Number of Fluorines
**27**	$1/(D^2)$	Number of Fluorines in alpha
**28**	$1/(D^2)$	Number of Fluorines in beta
**29**	$1/(D^2)$	Length of Carbon chain
**30**	$1/(D^2)$	Number of Hydrogens in the chain

Table S5. Definition of the computational descriptors used in the multivariate data analysis

**1**	D	Energy of the HUMO orbital of the molecule (water)
**2**	D	Energy of the LUMO orbital of the molecule (water)
**3**	D	HOMO - LUMO gap (n) (water)
**4**	D	Summation of the Mulliken charges (water)
**5**	D	Minimum value of the atomic Mulliken charges (water)
**6**	D	Maximum value of the atomic Mulliken charges (water)
**7**	D	Dipolar moment of the molecule (water)
**8**	D	DFT Free Energy with water as solvent

**9**	D	DFT Free Energy with n-octanol as solvent
**10**	D	Theoretical pKa
**11**	D	Volume of the molecule
**12**	D	Surface area of the molecule
**13**	D	Quantum polar surface area (QPSA) (0.01 isovalue)
**14**	D	Minimum value of the polar surface area (0.01 isovalue)
**15**	D	Maximum value of the polar surface area (0.01 isovalue)
**16**	D	Quantum polar surface area (QPSA) (0.05 isovalue)
**17**	D	Minimum value of the polar surface area (0.05 isovalue)
**18**	D	Maximum value of the polar surface area (0.05 isovalue)
**19**	D	Topological polar surface area (TPSA)
**20**	D	Mulliken Charge on the Sulphur in water
**21**	D	Mulliken Charge on the Sulphur in n-octanol
**22**	D	Difference of the Mulliken charge in water and in n-octanol
**23**	D	Energy of the HUMO orbital of the molecule (n-octanol)
**24**	D	Energy of the LUMO orbital of the molecule (n-octanol)
**25**	D	HOMO - LUMO gap (n) (n-octanol)
**26**	D	Summation of the Mulliken charges (n-octanol)
**27**	D	Minimum value of the atomic Mulliken charges (n-octanol)
**28**	D	Maximum value of the atomic Mulliken charges (n-octanol)
**29**	D	Dipolar moment of the molecule (n-octanol)
**30**	D	APT Charge on the Sulphur in water
**31**	D	NBO Charge on the Sulphur in water
**32**	D	High long pair electrons of the Sulphur
**33**	D	Low long pair electrons of the Sulphur
**34**	D	APT Charge on the Nitrogen in water
**35**	D	NBO Charge on the Nitrogen in water
**36**	D	Low long pair electrons of the Nitrogen
**37**	D	Mulliken Charge on the Nitrogen in water
**38**	Log(D)	Energy of the HUMO orbital of the molecule (water)
**39**	Log(D)	Energy of the LUMO orbital of the molecule (water)
**40**	Log(D)	HOMO - LUMO gap (n) (water)
**41**	Log(D)	Summation of the Mulliken charges (water)
**42**	Log(D)	Minimum value of the atomic Mulliken charges (water)
**43**	Log(D)	Maximum value of the atomic Mulliken charges (water)
**44**	Log(D)	Dipolar moment of the molecule (water)
**45**	Log(D)	DFT Free Energy with water as solvent
**46**	Log(D)	DFT Free Energy with n-octanol as solvent
**47**	Log(D)	Theoretical pKa
**48**	Log(D)	Volume of the molecule
**49**	Log(D)	Surface area of the molecule
**50**	Log(D)	Quantum polar surface area (QPSA) (0.01 isovalue)
**51**	Log(D)	Minimum value of the polar surface area (0.01 isovalue)

**52**	Log(D)	Maximum value of the polar surface area (0.01 isovalue)
**53**	Log(D)	Quantum polar surface area (QPSA) (0.05 isovalue)
**54**	Log(D)	Minimum value of the polar surface area (0.05 isovalue)
**55**	Log(D)	Maximum value of the polar surface area (0.05 isovalue)
**56**	Log(D)	Topological polar surface area (TPSA)
**57**	Log(D)	Mulliken Charge on the Sulphur in water
**58**	Log(D)	Mulliken Charge on the Sulphur in n-octanol
**59**	Log(D)	Difference of the Mulliken charge in water and in n-octanol
**60**	Log(D)	Energy of the HUMO orbital of the molecule (n-octanol)
**61**	Log(D)	Energy of the LUMO orbital of the molecule (n-octanol)
**62**	Log(D)	HOMO - LUMO gap (n) (n-octanol)
**63**	Log(D)	Summation of the Mulliken charges (n-octanol)
**64**	Log(D)	Minimum value of the atomic Mulliken charges (n-octanol)
**65**	Log(D)	Maximum value of the atomic Mulliken charges (n-octanol)
**66**	Log(D)	Dipolar moment of the molecule (n-octanol)
**67**	Log(D)	APT Charge on the Sulphur in water
**68**	Log(D)	NBO Charge on the Sulphur in water
**69**	Log(D)	High long pair electrons of the Sulphur
**70**	Log(D)	Low long pair electrons of the Sulphur
**71**	Log(D)	APT Charge on the Nitrogen in water
**72**	Log(D)	NBO Charge on the Nitrogen in water
**73**	Log(D)	Low long pair electrons of the Nitrogen
**74**	Log(D)	Mulliken Charge on the Nitrogen in water
**75**	1/D	Energy of the HUMO orbital of the molecule (water)
**76**	1/D	Energy of the LUMO orbital of the molecule (water)
**77**	1/D	HOMO - LUMO gap (n) (water)
**78**	1/D	Summation of the Mulliken charges (water)
**79**	1/D	Minimum value of the atomic Mulliken charges (water)
**80**	1/D	Maximum value of the atomic Mulliken charges (water)
**81**	1/D	Dipolar moment of the molecule (water)
**82**	1/D	DFT Free Energy with water as solvent
**83**	1/D	DFT Free Energy with n-octanol as solvent
**84**	1/D	Theoretical pKa
**85**	1/D	Volume of the molecule
**86**	1/D	Surface area of the molecule
**87**	1/D	Quantum polar surface area (QPSA) (0.01 isovalue)
**88**	1/D	Minimum value of the polar surface area (0.01 isovalue)
**89**	1/D	Maximum value of the polar surface area (0.01 isovalue)
**90**	1/D	Quantum polar surface area (QPSA) (0.05 isovalue)
**91**	1/D	Minimum value of the polar surface area (0.05 isovalue)
**92**	1/D	Maximum value of the polar surface area (0.05 isovalue)
**93**	1/D	Topological polar surface area (TPSA)
**94**	1/D	Mulliken Charge on the Sulphur in water

**95**	1/D	Mulliken Charge on the Sulphur in n-octanol
**96**	1/D	Difference of the Mulliken charge in water and in n-octanol
**97**	1/D	Energy of the HUMO orbital of the molecule (n-octanol)
**98**	1/D	Energy of the LUMO orbital of the molecule (n-octanol)
**99**	1/D	HOMO - LUMO gap (n) (n-octanol)
**100**	1/D	Summation of the Mulliken charges (n-octanol)
**101**	1/D	Minimum value of the atomic Mulliken charges (n-octanol)
**102**	1/D	Maximum value of the atomic Mulliken charges (n-octanol)
**103**	1/D	Dipolar moment of the molecule (n-octanol)
**104**	1/D	APT Charge on the Sulphur in water
**105**	1/D	NBO Charge on the Sulphur in water
**106**	1/D	High long pair electrons of the Sulphur
**107**	1/D	Low long pair electrons of the Sulphur
**108**	1/D	APT Charge on the Nitrogen in water
**109**	1/D	NBO Charge on the Nitrogen in water
**110**	1/D	Low long pair electrons of the Nitrogen
**111**	1/D	Mulliken Charge on the Nitrogen in water
**112**	D^2	Energy of the HUMO orbital of the molecule (water)
**113**	D^2	Energy of the LUMO orbital of the molecule (water)
**114**	D^2	HOMO - LUMO gap (n) (water)
**115**	D^2	Summation of the Mulliken charges (water)
**116**	D^2	Minimum value of the atomic Mulliken charges (water)
**117**	D^2	Maximum value of the atomic Mulliken charges (water)
**118**	D^2	Dipolar moment of the molecule (water)
**119**	D^2	DFT Free Energy with water as solvent
**120**	D^2	DFT Free Energy with n-octanol as solvent
**121**	D^2	Theoretical pKa
**122**	D^2	Volume of the molecule
**123**	D^2	Surface area of the molecule
**124**	D^2	Quantum polar surface area (QPSA) (0.01 isovalue)
**125**	D^2	Minimum value of the polar surface area (0.01 isovalue)
**126**	D^2	Maximum value of the polar surface area (0.01 isovalue)
**127**	D^2	Quantum polar surface area (QPSA) (0.05 isovalue)
**128**	D^2	Minimum value of the polar surface area (0.05 isovalue)
**129**	D^2	Maximum value of the polar surface area (0.05 isovalue)
**130**	D^2	Topological polar surface area (TPSA)
**131**	D^2	Mulliken Charge on the Sulphur in water
**132**	D^2	Mulliken Charge on the Sulphur in n-octanol
**133**	D^2	Difference of the Mulliken charge in water and in n-octanol

**134**	D^2	Energy of the HUMO orbital of the molecule (n-octanol)
**135**	D^2	Energy of the LUMO orbital of the molecule (n-octanol)
**136**	D^2	HOMO - LUMO gap (n) (n-octanol)
**137**	D^2	Summation of the Mulliken charges (n-octanol)
**138**	D^2	Minimum value of the atomic Mulliken charges (n-octanol)
**139**	D^2	Maximum value of the atomic Mulliken charges (n-octanol)
**140**	D^2	Dipolar moment of the molecule (n-octanol)
**141**	D^2	APT Charge on the Sulphur in water
**142**	D^2	NBO Charge on the Sulphur in water
**143**	D^2	High long pair electrons of the Sulphur
**144**	D^2	Low long pair electrons of the Sulphur
**145**	D^2	APT Charge on the Nitrogen in water
**146**	D^2	NBO Charge on the Nitrogen in water
**147**	D^2	Low long pair electrons of the Nitrogen
**148**	D^2	Mulliken Charge on the Nitrogen in water
**149**	$1/(D^2)$	Energy of the HUMO orbital of the molecule (water)
**150**	$1/(D^2)$	Energy of the LUMO orbital of the molecule (water)
**151**	$1/(D^2)$	HOMO - LUMO gap (n) (water)
**152**	$1/(D^2)$	Summation of the Mulliken charges (water)
**153**	$1/(D^2)$	Minimum value of the atomic Mulliken charges (water)
**154**	$1/(D^2)$	Maximum value of the atomic Mulliken charges (water)
**155**	$1/(D^2)$	Dipolar moment of the molecule (water)
**156**	$1/(D^2)$	DFT Free Energy with water as solvent
**157**	$1/(D^2)$	DFT Free Energy with n-octanol as solvent
**158**	$1/(D^2)$	Theoretical pKa
**159**	$1/(D^2)$	Volume of the molecule
**160**	$1/(D^2)$	Surface area of the molecule
**161**	$1/(D^2)$	Quantum polar surface area (QPSA) (0.01 isovalue)
**162**	$1/(D^2)$	Minimum value of the polar surface area (0.01 isovalue)
**163**	$1/(D^2)$	Maximum value of the polar surface area (0.01 isovalue)
**164**	$1/(D^2)$	Quantum polar surface area (QPSA) (0.05 isovalue)
**165**	$1/(D^2)$	Minimum in the polar surface area (0.05 isovalue)
**166**	$1/(D^2)$	Maximum in the polar surface area (0.05 isovalue)
**167**	$1/(D^2)$	Topological polar surface area (TPSA)
**168**	$1/(D^2)$	Mulliken Charge on the Sulphur in water
**169**	$1/(D^2)$	Mulliken Charge on the Sulphur in n-octanol

**170**	$1/(D^2)$	Difference of the Mulliken charge in water and in n-octanol
**171**	$1/(D^2)$	Energy of the HUMO orbital of the molecule (n-octanol)
**172**	$1/(D^2)$	Energy of the LUMO orbital of the molecule (n-octanol)
**173**	$1/(D^2)$	HOMO - LUMO gap (n) (n-octanol)
**174**	$1/(D^2)$	Summation of the Mulliken charges (n-octanol)
**175**	$1/(D^2)$	Minimum value of the atomic Mulliken charges (n-octanol)
**176**	$1/(D^2)$	Maximum value of the atomic Mulliken charges (n-octanol)
**177**	$1/(D^2)$	Dipolar moment of the molecule (n-octanol)
**178**	$1/(D^2)$	APT Charge on the Sulphur in water
**179**	$1/(D^2)$	NBO Charge on the Sulphur in water
**180**	$1/(D^2)$	High long pair electrons of the Sulphur
**181**	$1/(D^2)$	Low long pair electrons of the Sulphur
**182**	$1/(D^2)$	APT Charge on the Nitrogen in water
**183**	$1/(D^2)$	NBO Charge on the Nitrogen in water
**184**	$1/(D^2)$	Low long pair electrons of the Nitrogen
**185**	$1/(D^2)$	Mulliken Charge on the Nitrogen in water

8. Data sets used for the statistical analysis

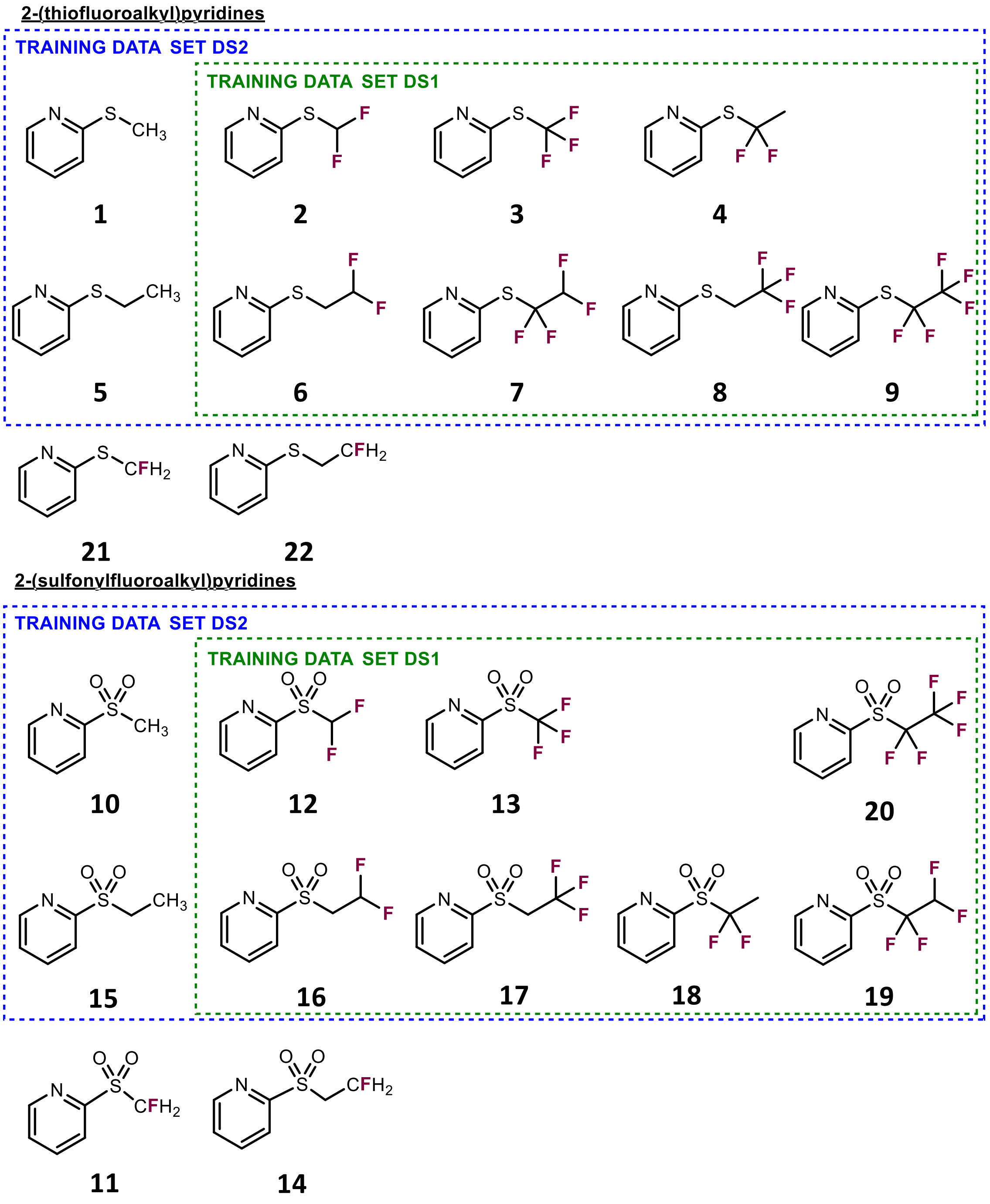

Figure S1. 2D structure representation of the compounds in data sets **DS1** and **DS2** used in the statistical analysis

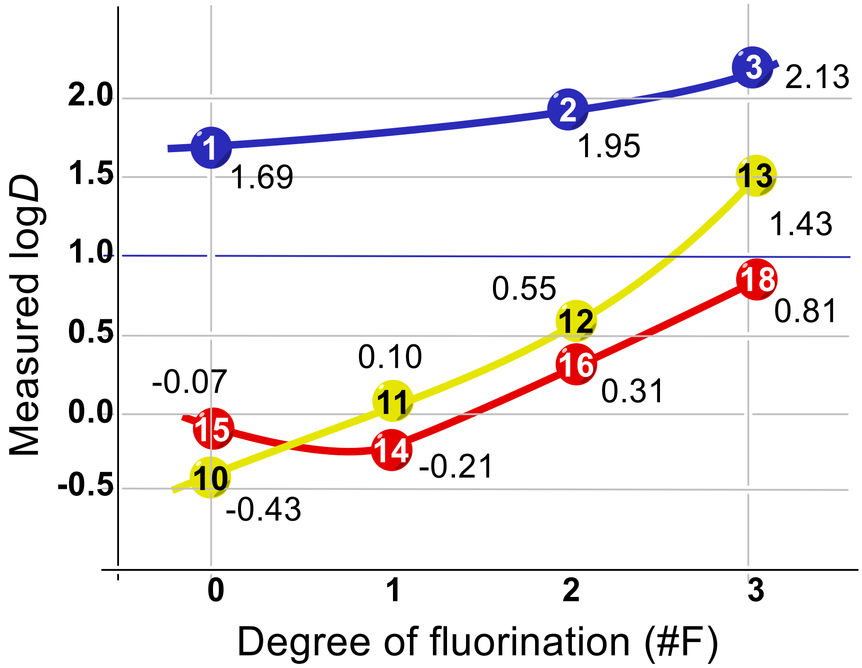

Figure S2. Juxtaposition of log$D^{7.4}$ dependency on the degree of alkyl fluorination (number of fluorine atoms, #F) for 3 series of compounds: (1) methyl thioethers **1-3** (blue line, exponential profile); (2) methyl sulfones **10-13** (yellow line, exponential profile), and (3) and ethyl sulfones **14**, **15**, **16**, and **18** (red line, parabolic profile)

9. Statistical analysis

9.1. Regression process

To build the regression model, we wrote a Python code, using libraries: NumPy, pandas, scikit-learn, and Matplotlib. Using as reference experimental measures, we built two data sets, one containing the 2-(thiofluoroalkyl)pyridines **2**, **3**, **4**, **6**, **7**, **8**, **9** and the corresponding 2-(sulfonylfluoroalkyl)pyridines **12**, **13**, **16**, **17**, **18**, **19**, **20** (**DS1** data set 1, 14 samples), the other adding also non-fluorinated compounds **1**, **5**, **10**, and **15** (**DS2**, data set 2, 18 samples), see Figure S1. Unless otherwise stated the data presented corresponds to the model built with the compounds of data set **DS2**. As described in the section Descriptors Used in the Multivariate Data Analysis of this Supporting Information, a descriptors dataset containing 215 entries was obtained. Subsequently, we generated over 23000 regression models.

To assess collinearity among descriptors, we conducted a regression model for all possible combinations of descriptors, filtering out those with an R^2 exceeding 0.6. Additionally, we employed the Variance Inflation Factor (VIF) as a diagnostic tool to identify collinearity. The VIF formula is given by:

$$VIF\beta^j = \frac{1}{1 - R^2}$$

A VIF close to 1 indicates a total absence of collinearity, while values between 1 and 10 suggest potential minor collinearity. VIF values exceeding 10 are indicative of significant collinearity. The

correlation matrix, visually represented, facilitated a comprehensive understanding of the relationships among descriptors. After this analysis from the > 23000 regressions, 904 were excluded, with a VIF > 10. For further analysis we focused on the models with an R^2 greater than 0.8 (~1000). Selected regression models were carefully analyzed and their meaning and underlying trends chemically interpreted.

9.2. Code used for statistical analysis

The python code written on purpose for this project was developed. It is uploaded and available on GitHub: https://github.com/gonzaurv/logD_descriptors_prediction, together with detailed information about how to use it, as well as the datasets used for this project.

10. Regression model selection

10.1. Selected model overview.

As detailed in the main text, after the extensive analysis of the regression models, we selected the model with the number of fluorine atoms (**#F**) and the inverse of calculated Mulliken charge at the sulfur (**1/q$_s$**), as was able to predict and explain the experimental trends. In **Figure S3**, four substrates with different experimental log$D^{7.4}$ (compounds **6**, **7**, **8** and **9**) are shown and the equation obtained for the selected model is presented as function of the two descriptors. We can also see that the log$D^{7.4}$ increases with the number of fluorine atoms, but as this happens the charge on the sulfur also increases, due to the electron density withdrawing from fluorines. And with this the descriptor **1/q$_s$** decreases and decreases the log$D^{7.4}$.

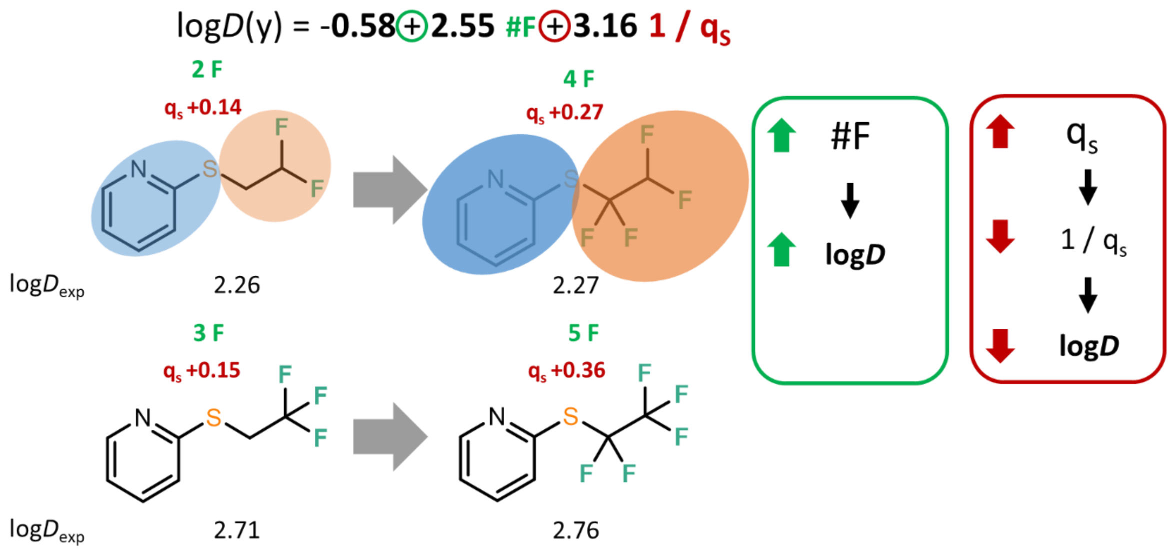

Figure S3. Top, equation derived from the multivariate data analysis for the selected model. Bottom, schematic representation of the selected model: number of fluorine atoms (**#F**) and inverse of the calculated Mulliken charge at the sulfur atom (**1/q$_s$**). Intensity of the colors refers to electron density of each fragment of the substrate

10.2. Response variable and descriptors values for the model

As previously mentioned in the Descriptors Used in the Multivariate Data Analysis section of this Supporting Information, all descriptors have been normalized. For the selected regression model, with the number of atoms (**#F**) and the inverse of the calculated Mulliken charge at the sulfur atom (**$1/q_s$**), the crude descriptors before the pre-processing, and the normalized descriptors after data pre-processing are presented in Table S6 for each compound. The experimental log$D^{7.4}$ and the substrate number are also depicted. The values for the training set **DS1** are shown in white background. In blue background, we show the non-fluorinated compounds (**1**, **5**, **10** and **15**) used for constructing training set **DS2**. The values in orange background correspond to compounds used for the training set. At the bottom of Table S6 substrates not experimentally measured (**21** and **22**) are also presented.

Table S6. Crude and Normalized values of descriptors **#F** (number of fluorine atoms) and **$1/q_s$** (inverse of the calculated Mulliken charge at the sulfur atom)

		Crude descriptors		Normalized descriptors	
**Substrate**	**log$D^{7.4}_{exp}$**	**#F**	**$1/q_s$**	**#F**	**$1/q_s$**
**1**	1.69	0	8.8257	0.0	0.6733
**2**	1.95	2	6.2966	0.4	0.4803
**3**	2.13	3	3.6577	0.6	0.2790
**4**	1.82	2	5.4765	0.4	0.4178
**5**	2.26	0	13.108	0.0	1.0000
**6**	2.26	2	6.9678	0.4	0.5315
**7**	2.27	4	3.6949	0.8	0.2819
**8**	2.71	3	6.7983	0.6	0.5186
**9**	2.76	5	2.7377	1.0	0.2088
**10**	-0.43	0	0.8709	0.0	0.0664
**11**	0.10	1	0.8488	0.2	0.0647
**12**	0.55	2	0.9380	0.4	0.0716
**13**	1.43	3	1.0538	0.6	0.0804
**14**	-0.21	1	0.9370	0.2	0.0715
**15**	-0.07	0	0.9122	0.0	0.0696
**16**	0.31	2	0.8973	0.4	0.0685
**17**	0.79	2	1.1224	0.4	0.0856
**18**	0.81	3	0.9200	0.6	0.0702
**19**	1.43	4	1.0119	0.8	0.0772
**20**	2.21	5	0.8473	1.0	0.0646
**21**	(a)	1	8.2563	0.2	0.6298
**22**	(a)	1	7.8118	0.2	0.5959

(a) Not synthetized, decompose in water.

10.3. The model results

The selected regression model can reproduce the chemical behavior of the substrates, namely the log$D^{7.4}$, when applied on both training data sets **DS1** and **DS2**. In Table S7, the calculated log$D^{7.4}$ values are collected together with their errors respect to the experimental ones.

Table S7. Values for experimental log$D^{7.4}$ (**log*D*$_{exp}$**), calculated log$D^{7.4}$ using training set **DS1** (**log*D*$_{DS1}$**), and calculated log$D^{7.4}$ using training set **DS2** (**log*D*$_{DS2}$**). Square of correlation coefficient (R^2), root-mean-square deviation (RMSE), and mean absolute error (MAE) for the two data sets

	experimental	calculated			
		DS1		DS2	
**Substrate**	**log*D*$^{7.4}_{exp}$**	**log*D*$_{DS1}$**	ΔΔlogD_{DS1}	**log*D*$_{DS2}$**	ΔΔlogD_{DS2}
**1**	1.69	1.67	-0.02	1.55	-0.14
**2**	1.95	2.05	+0.10	1.95	0.00
**3**	2.13	1.85	-0.28	1.83	-0.30
**4**	1.82	1.82	0.00	1.76	-0.06
**5**	2.26	2.89	+0.63	2.58	+0.32
**6**	2.26	2.24	-0.02	2.12	-0.14
**7**	2.27	2.41	+0.14	2.35	+0.08
**8**	2.71	2.74	+0.03	2.59	-0.12
**9**	2.76	2.68	-0.08	2.63	-0.13
**10**	-0.43	-0.58	-0.15	-0.37	+0.06
**11**	0.10	-0.04	-0.14	0.15	+0.05
**12**	0.55	0.54	-0.01	0.66	+0.11
**13**	1.43	1.11	-0.32	1.20	-0.23
**14**	-0.21	-0.01	+0.20	0.13	+0.34
**15**	-0.07	-0.57	-0.50	-0.36	-0.29
**16**	0.31	0.52	+0.21	0.65	+0.34
**17**	0.79	0.59	-0.20	0.71	-0.06
**18**	0.81	1.08	+0.27	1.17	+0.36
**19**	1.43	1.65	+0.22	1.70	+0.27
**20**	2.21	2.15	-0.06	2.17	-0.04
**21**	a)	2.06	-	1.92	-
**22**	a)	1.93	-	1.81	-
**R^2**		0.95		0.95	
**RMSE**		0.03		0.04	
**MAE**		0.14		0.17	

(a) Not synthetized, decomposes in water.

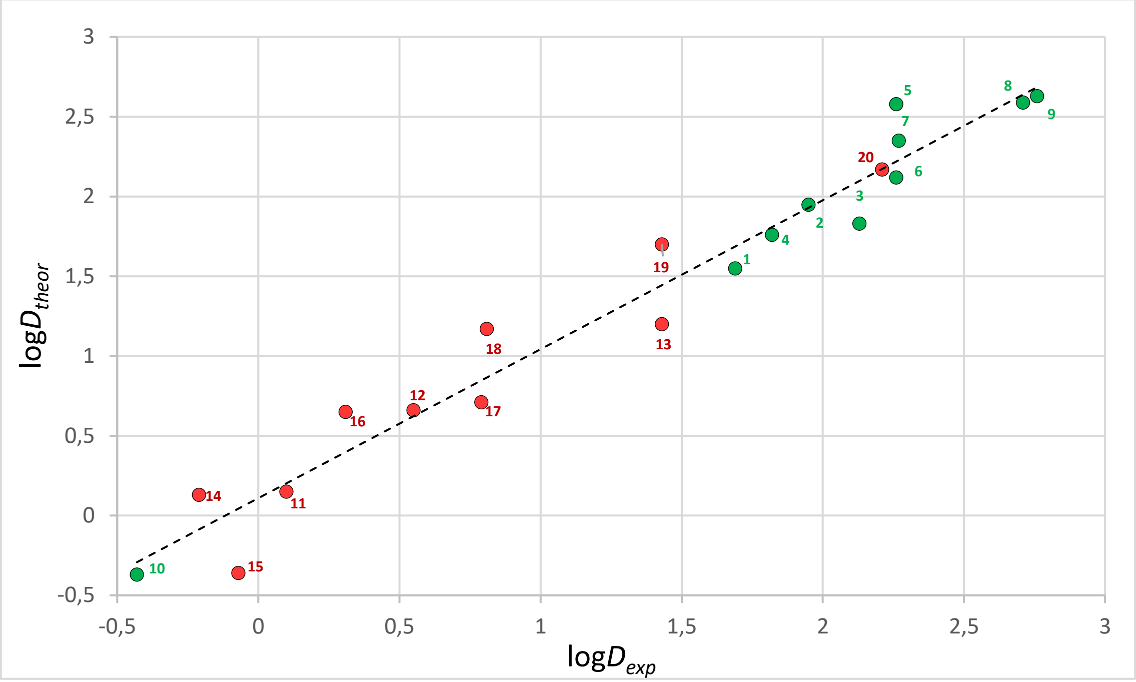

Figure S4. Experimental *vs.* predicted log*D* values using the regression model derived from data set **DS2**. Green and red dots correspond to thioether and sulphonyl compound, respectively

10.4. Descriptor's weight. Key to understand the model

Observing the experimental log$D^{7.4}$ of the studied substrates it is easy to see that oxidized and non-oxidized substrates present significant differences and follow different trends. Analyzing the regression model this difference can also be observed. For oxidized substrates, the regression model utilizing the descriptors **#F** (number of fluorine atoms) and **1/q$_s$** (inverse of the calculated Mulliken charge at the sulfur atom) is predominantly influenced by the **#F**, while the second descriptor shows negligible impact across the various compounds. Conversely, in non-oxidized substrates, both descriptors exert influence on the log$D^{7.4}$ prediction. This can be observed in Figure S5, where the descriptors weights (*descriptor weight* = *regression coefficient* x *descriptor value*) is plotted for the two selected descriptors.

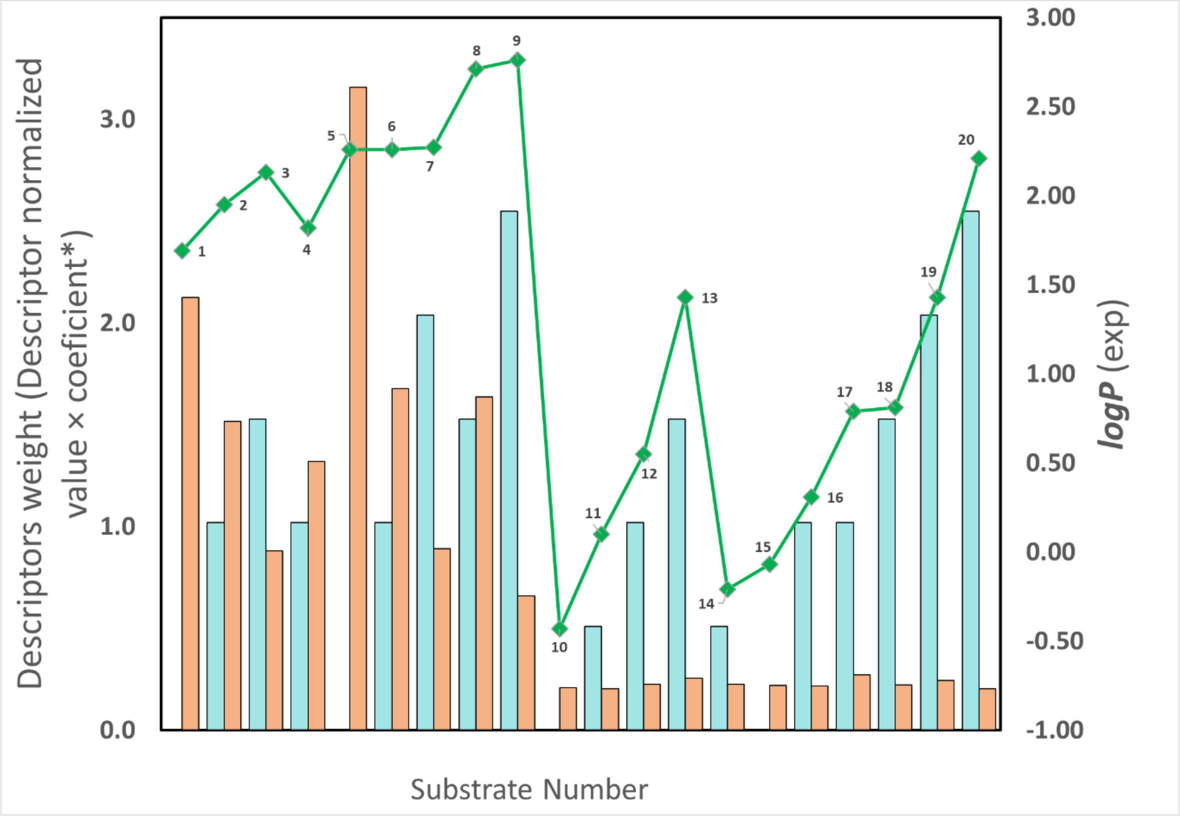

Figure S5. Plot of the descriptors weights for each descriptor using data set **DS2**. In orange weights corresponding to the second coefficient (3.16) by **1/q$_s$** for each substrate. In cyan weight corresponding to first coefficient (2.55) by **#F**. The left area of the graph corresponds to non-oxidized compounds, and the right to the oxidized compounds

11. Other Tested Models

Initially, univariate regressions were tested with traditional descriptors for estimating lipophilicity of organic molecules. This descriptor includes Topological polar surface area (TPSA) and Quantum polar surface area (QPSA), described above.

11.1. Model 2: TPSA

Topological polar surface area (TPSA) has been used in chemistry to predict lipophilicity.[6–9] TPSA quantifies the part of a molecule's surface area occupied by polar atoms or functional groups able to participate in hydrogen bonds or other polar interactions. The calculation considers the contributions from the different polar functional groups within the molecule under study (hydroxyl, amino, carbonyl, nitro groups). Estimation of the contribution of each functional group generally relies on empirical equations based on large data sets or algorithms primarily based on simple atomic parameters such as atomic radio or partial charges.

Model 2 consists of the application of TPSA as descriptor in linear regressions to predict the lipophilicity of dataset **DS2,** the results for this model are presented in Table S8. The coefficient of

determination obtained is quite low (0.63) the root-mean square deviate-on (RMSE) quite large (0.56), and the Mean Absolute Error (MAE) 0.41. RMSE for this model is twice that found for the above-described selected model (results presented in Table S7) and the MAE is also much larger. Looking at the reported errors for each substrate (Table S7), it is clear that the predictive power of this approach is low for the studied set of substrates.

11.2. Model 3: QMPSA

The quantum polar surface area (QMPSA) is an index used in computational chemistry for measuring molecular polarity. Like TPSA, QMPSA evaluates the surface of the molecule containing polar atoms or functional groups and has also been used to predict lipophilicity.[10–14] QMPSA calculations needs van der Waals radii and electrostatic potentials obtained from quantum mechanics calculations.

The results obtained when using QPSA as descriptor, Model 3, are presented in Table S8. Errors are smaller than for TPSA (Model 2), although correlation is smaller. Errors are 0.56 for the RMSE and 0.54 for the MAE. It is also clear that the predictive power of this approach is too low for the studied set of substrates.

11.3. Model 4: Traditional DFT-based log*D* prediction

Model 4 is based on the free energies in solution obtained from Density Functional Theory (DFT) calculation. This type of log*D* prediction, is a well-established method employed in recent years for lipophilicity predictions in pharmaceutical and pesticides chemistry.[15–17] The equation used within for this approach is:

$$DFT(logD) = \frac{\Delta G_{\text{water}} - \Delta G_{\text{n-octanol}}}{2.303\ RT}$$

Results obtained using Model 4 are presented also in Table S8. The coefficient of determination is low, and the errors are large. Even higher than the errors obtained by the simple TPSA. Hence the use of this method has to be also discarded for the prediction of substrates within the studied set of substrates.

Table S8. Experimental values of log$D^{7.4}$ (**logD_{exp}**) and calculated log$D$ values using Model 2 (TPSA descriptor), Model 3 (QPSA descriptor) and Model 4 (DFT-based log$D$ calculation). In all cases the training set used is data set **DS2**

		**Model 2**		**Model 3**		**Model4**	
**Substrate**	**logD_{exp}**	**logD_{M2}**	ΔΔlogD_{M2}	**logD_{M3}**	ΔΔlogD_{M3}	**logD_{M4}**	ΔΔlogD_{M4}
**1**	1.69	1.85	+0.16	2.40	+0.73	2.31	+0.62
**2**	1.95	1.85	-0.10	1.80	-0.15	2.10	+0.15
**3**	2.13	2.13	0.00	2.28	+0.15	2.01	-0.12
**4**	1.82	2.13	+0.31	1.73	-0.09	1.82	0.00
**5**	2.26	2.26	0.00	2.38	+0.12	2.26	0.00
**6**	2.26	2.29	+0.03	1.77	-0.49	2.12	-0.14
**7**	2.27	2.29	+0.02	1.78	-0.49	2.09	-0.18
**8**	2.71	2.25	-0.46	2.37	-0.34	2.17	-0.64
**9**	2.76	2.16	-0.60	2.16	+0.60	2.03	-0.74
**10**	-0.43	0.73	+1.16	1.04	+1.47	1.37	+1.80
**11**	0.10	0.74	+0.64	1.12	+1.02	-0.03	-0.13
**12**	0.55	0.74	+0.19	0.84	+0.29	1.40	+0.85
**13**	1.43	0.73	-0.70	0.96	-0.47	1.69	+0.26
**14**	-0.21	0.79	+1.00	0.62	+0.83	1.37	+1.58
**15**	-0.07	0.74	+0.81	1.06	+1.13	1.36	+1.43
**16**	0.31	0.77	+0.46	1.08	+0.77	1.42	+1.11
**17**	0.79	0.83	+0.04	0.92	+0.13	1.51	+0.72
**18**	0.81	0.78	-0.03	0.92	+0.11	1.40	+0.59
**19**	1.43	0.88	-0.55	0.53	-0.90	1.48	+0.05
**20**	2.21	0.75	-1.46	0.86	-1.35	1.74	+0.47
**21**	(a)	2.21	-	1.88	-	2.10	-
**22**	(a)	2.27	-	1.88	-	2.01	-
**R^2**		0.60		0.42		0.58	
**RMSE**		0.34		0.47		0.73	
**MAE**		0.42		0.54		0.54	

(a) Not synthetized, decompose in water.

11.4. Energy-weighted descriptors values for the different isomers

We also performed an in-depth analysis of effect of conformational isomers on the performance of the regression models. As shown previously, there are significant differences in lipophilicity between the conformers of a given molecule.[18–23] The conformational search protocol for generating the different structures is explained in the Computational Details section of the main text. Two distinct prediction approaches were used to enhance our understanding of these molecular systems.

1. **Approach 1**: This approach was required only to determine the most stable (lowest energy) isomer/conformer for each molecule. And only this single most stable isomer was used to extract descriptors.
2. **Approach 2**: The second approach needed a more thorough analysis. A weighted value of each descriptor was used instead of a single value. The weighted value was obtained considering that all isomers of each molecule with a significant representation at a temperature of 25 °C would contribute on the descriptors. Hence descriptors were obtained for all isomers and then weighted following a Boltzmann distribution of states.

The results obtained, are presented in Table S9. There is no significant difference between the two approaches **#1** and **#2**. As a result, models based on low-energy design, widely accepted and frequently used, are shown to be a suitable choice for the prediction and regression analysis. This analysis was done for substrate in data set **DS1** (14 substrates).

Table S9. Experimental log$D^{7.4}$ and calculated log$D$ obtained with the selected regression model, with the **#F** and **1/q$_s$** and the approaches **#1** and **#2**

substrate	log$D^{7.4}_{exp}$ (a)	Approach 1<br>log$D_{minimum\text{-}energy}$	Approach 2<br>log$D_{weighted}$
**2**	1.95	2.05	1.95
**3**	2.13	1.85	1.85
**4**	1.82	1.82	1.71
**6**	2.26	2.24	2.25
**7**	2.27	2.41	2.38
**8**	2.71	2.74	2.72
**9**	2.76	2.68	2.69
**12**	0.55	0.55	0.48
**13**	1.43	1.11	1.20
**16**	0.31	0.52	0.47
**17**	0.79	0.59	1.03
**18**	0.81	1.08	1.08
**19**	1.43	1.65	1.63
**20**	2.21	2.15	2.16
**R^2**		0.95	0.95
**RMSE**		0.03	0.03
**MAE**		0.14	0.13

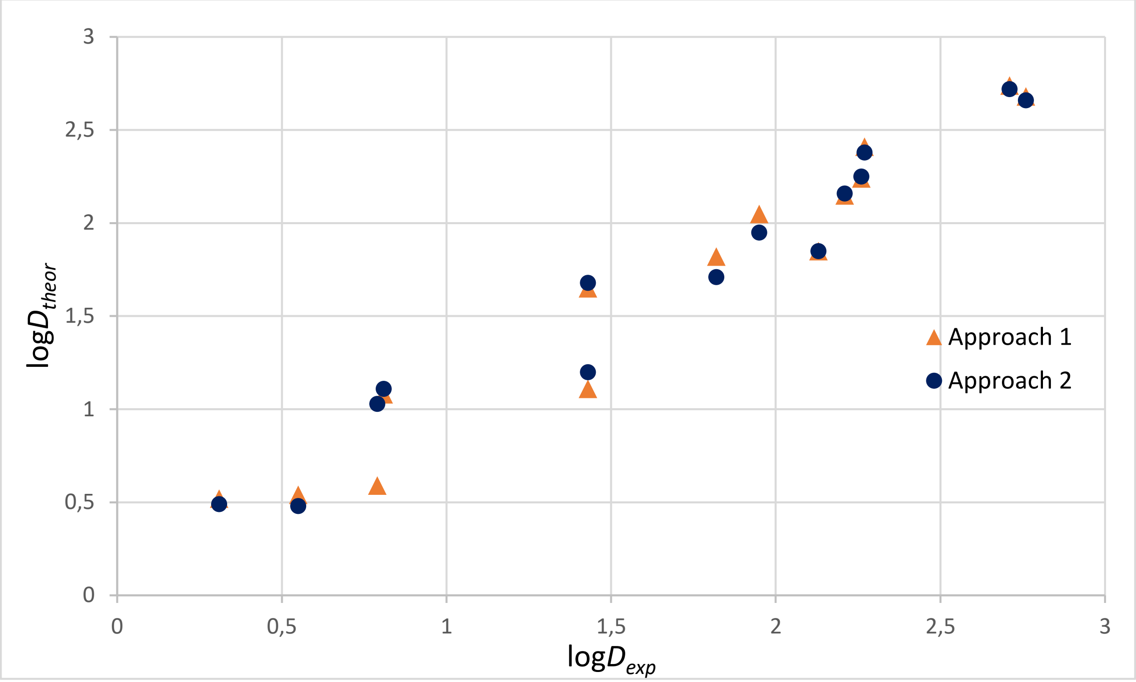

Figure S6. Experimental *vs.* predicted log*D* values using regression model with **#F** and **1/q$_s$** descriptors and data set **DS1**. Comparison of approach **#1** (lowest-energy isomers) and **#2** (energy-weighted values).

12. Relevance of hydrogen bonds in the studied substrates

Hydrogen bonds (H-bonds)[24,25] have been proposed to be a key factor in determining the lipophilicity of some sulfur-containing compounds, particularly those with fluorinated alkyl groups. Previous studies have reported that fluorinated groups act as weak H-bond donor modulators, enhancing the lipophilicity of the compounds[26,27], especially in functional groups such as sulfides, sulfoxides, and sulfones[28,29,30]. These studies support that the interplay between fluorination, H-bond basicity, and molecular volume is crucial for predicting the behavior of these compounds. To further understand the role of IntraMolecular Hydrogen Bonds (IMHBs)[31] and their impact on the physicochemical properties of the substrates, we computationally studied the presence and strength of IMHBs in our series of 2-SR$_F$ and 2-SO$_2$R$_F$ substituted pyridines. To do so, we evaluated the key bond lengths in the most stable conformers, carried Bader's Atoms in Molecules (AIM) analysis and plot maps of Non-Covalent Interaction (NCI). In Table S10, the shortest N··H bond length and the N··H-C angle for each compound is presented when smaller than 3.0 Å. Compounds **3**, **9**, **13** and **20** have not been included in the table as there are no hydrogens in the -SR or -SO$_2$R moiety, as they are fully fluorinated. The presence of N··H critical points, following Bader's Atoms in Molecules (AIM)[32] analysis, is also reported in the table. AIM uses the topology of the electron density to identify chemical bonds. By studying the electron density distribution and its critical points, non-covalent interactions like hydrogen bonds/interactions can be characterized. For hydrogen bonds/interactions, the presence of a (3,−1) bond critical points (BCPs) between the hydrogen and the acceptor provides quantitative evidence of hydrogen bonding[33]. The strength of the N··H bond/interaction can be measured with the electron charge density ρ(r) at the BCP, the presence of BCPs and their electron

charge density ρ(r) is also collected in Table S10. On top of this, NCI plots[34,35,], a visualization index based on the electron density and its derivatives, have been used to reveal the presence of weak interactions in these compounds. These plots enable the identification of non-covalent interactions on the basis of peaks that appear in the reduced density gradient (RDG) at low densities, and represent attractive interactions in blue, weak interactions in green and repulsive interactions in red. Strong hydrogen bonds appear as clearly defined blue circles, weak hydrogen bonds appear as bluish-green circles while weaker van der Waals interactions are presented in green surfaces in different shapes and steric effects are orange or red depending on their strength. Based on the analysis performed, very few compounds present hydrogen bonds with medium strength, just compounds **7** and **8** (Figure S7); weak hydrogen bonds/interactions are present in **2**, **6**, **18**, **19**, **21**, and **22** (Figure S7 and S8). No BCP between N and H is located in compounds **1**, **4**, **5**, **10**, **11**, **12**, **14**, **15**, **16**, and **17**, which means that either there is not a hydrogen bond/interaction or it is very weak.

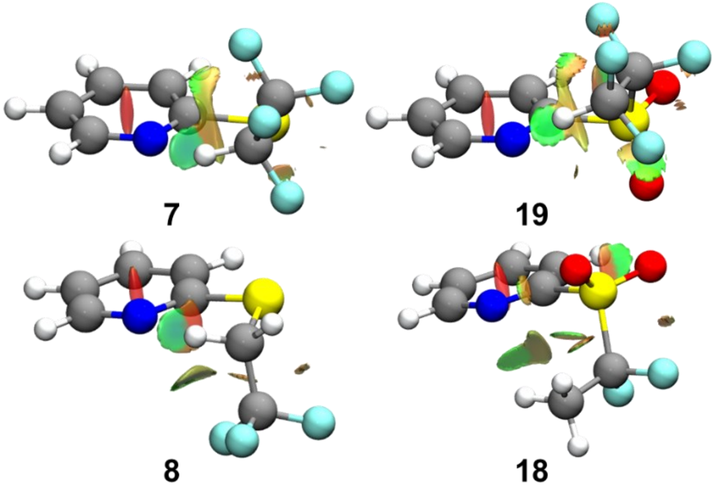

Figure S7. Non-covalent interactions (NCI) plots for compounds **7**, **8**, **18**, and **19**, in blue attractive interactions, in green weak interactions and in red repulsive interactions

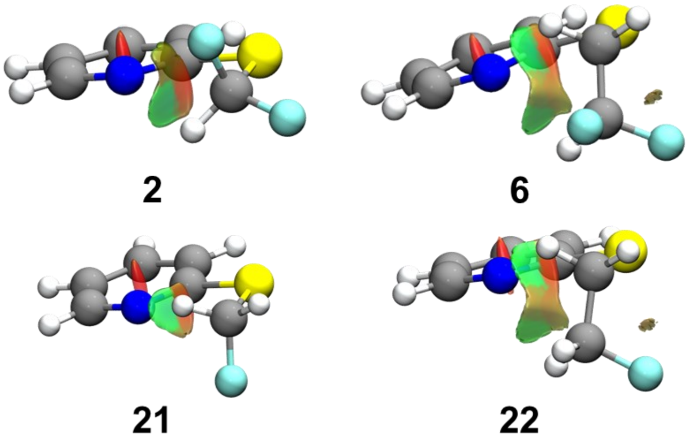

Figure S8. Non-covalent interactions (NCI) plots for compounds **2**, **6**, **21**, and **22**, in blue attractive interactions, in green weak interactions and in red repulsive interactions

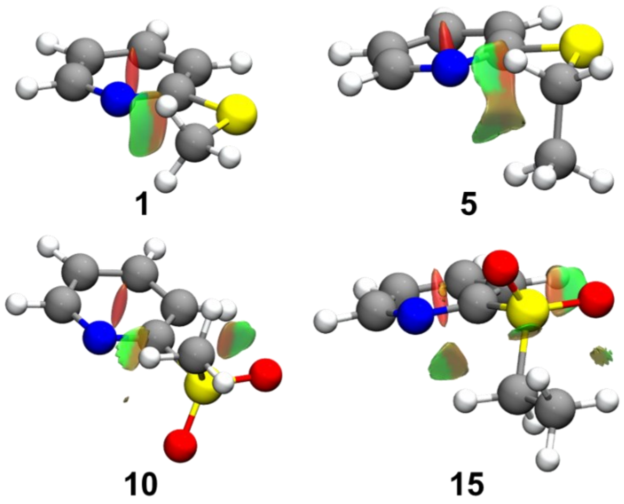

Figure S9. Non-covalent interactions (NCI) plots for non-fluorinated compounds **1**, **5**, **10**, and **15**, in blue attractive interactions, in green weak interactions and in red repulsive interactions

The two compounds, **7** and **8,** have hydrogen bonds of medium strength, they are constituted by moieties -S-CF_2CF_2H and -S-CH_2CF_3, see Figure S6. These same two alkyl moieties in sulfones, -SO_2-CF_2CF_2H and -SO_2-CH_2CF_3, compounds **19** and **18**, present only weak hydrogen bonds/interactions. It is interesting to note that no other sulfone (-SO_2R) has an interaction of significant strength (no BCP). Apart from sulfones, S-R compounds that don't present hydrogen bonds are: non-fluorinated alkyl chains (compounds **1** and **5**, see Figure S9) and S-CF_2CH_3 (compound **4**).

If we have a look at general trends, we can see that substitution of two hydrogens by two fluorine atoms in non-fluorinated compounds **1** and **5**, -S-CH_3 and -S-CH_2CH_3, leads to compounds with and without hydrogen bonds: -S-CF_2H (compound **2**) and -S-CH_2CF_2H (compound **6**) present weak hydrogen bonds, however -S-CF_2CH_3 (compound **4**) and all equivalent SO_2R compounds with two fluorines, present no hydrogen bond.

Based on computed N··H distances and the electron charge density, ρ(r), at the N··H bond critical point, we can order the strength of the hydrogen bonds/interactions of the studied -S-R compounds. The strength follows -S-CF_2CF_2H > -S-CH_2CF_3 > -S-CF_2H > -S-CFH_2 > -S-CH_2CF_2H > -S-CH_2CFH_2 (compounds **8**, **7**, **2**, **21**, **6** and **22**, Figure S6 and S7), while as mentioned, for -S-CF_2CH_3, -S-CH_2CH_3 and -S-CH_3 (compounds **4**, **5** and **1**), no BCP was located.

Table S10. log$D^{7.4}$ experimental values, normalized values of descriptors **#F** (number of fluorine atoms), **1/q$_s$** (inverse of the calculated Mulliken charge at the sulfur atom) and H··N distances (between the Nitrogen of the pyridine with the closest Hydrogen of the alkyl chain), N··H-C angles, presence of bond critical point and ρ(r) electron charge density

Compound	log$D^{7.4}_{exp}$	Norm. descriptor #F	Norm. descriptor 1/q$_s$	Distance H···N [b]	Angle (degree) N···H-C	ρ(r) electron charge density at the H··N BCP[c]
**1**	1.69	0.0	0.67254	2.801	83.4	No BCP
**2**	1.95	0.4	0.47982	2.442	99.2	0.014
**4**	1.82	0.4	0.41721	-	-	-
**5**	2.26	0.0	1.00000	2.553	98.2	No BCP
**6**	2.26	0.4	0.53101	2.528	96.4	0.009
**7**	2.27	0.8	0.28156	2.355	123.7	0.015
**8**	2.71	0.6	0.51835	2.310	111.6	0.017
**10**	-0.43	0.0	0.06636	2.827	98.4	No BCP
**11**	0.10	0.2	0.06467	2.792	60.7	No BCP
**12**	0.55	0.4	0.14127	2.849	64.8	No BCP
**14**	-0.21	0.2	0.07140	2.617	109.6	No BCP
**15**	-0.07	0.0	0.06478	2.787	102.3	No BCP
**16**	0.31	0.4	0.06838	2.741	103.5	No BCP
**17**	0.79	0.4	0.08553	2.639	124.6	No BCP
**18**	0.81	0.6	0.07010	2.645	105.2	0.008
**19**	1.43	0.8	0.07711	2.463	125.2	0.012
**21**	(a)	0.2	0.62194	2.436	103.0	0.014
**22**	(a)	0.2	0.59527	2.526	110.7	0.008

(a) Not synthetized, decomposes in water. Calculated values of log$D^{7.4}$ for species **21** and **22**, 1.92 and 1.81 respectively. (b) Distances in Angstroms. (c) BCP = Bond Critical Point (Atoms In Molecules analysis).

The analysis can also be done for the different conformers of the same compound. NCI plots for compound **6** (log$D^{7.4}$ 2.26) show at the geometry of its lowest energy conformer (left, Figure S10) two weak attractive interactions, $CF_2H\cdots N_{pyridyl}$ and $CH_2\cdots N_{pyridyl}$ with 2.528 and 2.651 Å and the Bader's Atoms In Molecules analysis shows only one bond critical point for $CF_2H\cdots N_{pyridyl}$ with ρ(r) = 0.009. At the geometry of the second most stable conformer (right, Figure S10), a stronger IMHB, $CH_2\cdots N_{pyridyl}$ with 2.441 Å distance can be observed and a BCP was located with ρ(r) = 0.017. Then, it is interesting to note that the conformer with the strongest hydrogen bond is 1.1 kcal.mol^{-1} higher in energy than the conformer with a weaker hydrogen bond, highlighting other interactions different to hydrogen bonds must also play a role to structure and physicochemical properties.

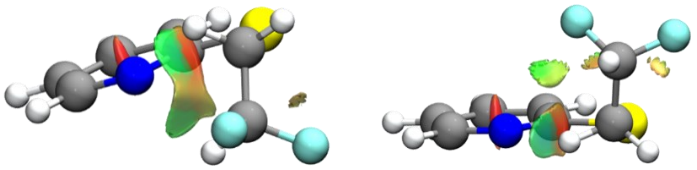

Figure S10. Non-covalent interaction (NCI) plots for the two lowest conformers of compound **6**: left, most stable conformer, right the second most stable conformer. In blue attractive interactions, in green weak interactions and in red repulsive interactions

Finally, we analyzed the role of the IMHBs on the lipophilicity. This is a complex analysis as it is hard to separate properties and determine causality. Hydrogen bond strength is associated to properties such as polarity or charges, that are also associated to lipophilicity. On top of that they altogether have an impact to structure, that indeed also plays a role in lipophilicity. Analyzing the data obtained we can see that: a) from one hand, the hydrogen bonds/interactions presented are in general very weak, also that other weak interactions might also have importance in lipophilicity; b) our results in general support that in most cases larger log$D^{7.4}$s are accompanied by stronger hydrogen bonds (compounds **8** > **7** > **6** > **4**) with exceptions; c) but, on the other hand some compounds like **5** and **6** have the same log$D^{7.4}$ (2.26) but one presents a hydrogen bond (compound **6**) and the other doesn't (compound **5**), d) similarly, compound **9** has the largest log$D^{7.4}$ but does not present any hydrogen bond (it is fully fluorinated) although it does present other weak interactions; e) changes in lipophilicity between SR and SO_2R cannot only be explained by strength of hydrogen bonds or interactions, as compounds with similar hydrogen bonds have lower lipophilicity for sulfones.

13. Comparison of lipophilicity with literature precedents

We can compare lipophilicity trends with those reported for thiofluoroalkyl benzenes by the O'Hagan group,[36] which are analogs of compounds **1**, **3**, **4**, **5**, and **8** (Table S11). In this latter report, log*P* (=log$D^{7.4}$ in this case) was measured indirectly by an HPLC method based on the retention times of the compounds.

Table S11. Comparison of log$D^{7.4}$ values between Ph and Py substituents.

SR_F	log$D^{7.4}$	
	Ph	Py
SCF_3	3.70	2.13
SCH_3	2.87	1.69
SCH_2CH_3	3.42	2.26
SCF_2CH_3	3.38	1.82
SCH_2CF_3	3.30	2.71

Overall, the log*D* values of our thioether pyridine compounds are lower than those for analogous complexes with phenyl group. The reason is that pyridyl is more polar than phenyl group and can act

as hydrogen acceptor resulting in an overall reduction of lipophilicity. Similar to our observations, for methyl thioethers, the tri-fluorination yields to a significant increase of log*D* value, while for ethyl thioethers, the α-fluorination lowers the log*D* value. Contrary to thioether pyridines, β-fluorination of ethyl fragment results in a decrease of log*D* value in the case of thioether benzene compounds, the latter trend being somewhat counterintuitive.

14. pK_a predictions

With the same descriptors used for the prediction of the log$D^{7.4}$, **#F** and **1/q$_s$**, we attempted the prediction of the experimental pK_as. As there is no experimental pK_a data available for a large number of compounds a new data set was used, **DS3** defined in Table S12. Results obtained are presented in Table S12. These results are quite interesting as the same descriptors used to predict the experimental log$D^{7.4}$ are able to predict with moderate errors the experimental pK_as of the studied substrates. The equation obtained in the multivariate regression for this model is the following:

$$pKa = 2.14 - 1.91 \cdot \mathbf{\#F} + 1.60 \cdot \mathbf{1/qs}$$

Table S12. Experimental and calculated pK_a values obtained with the regression model using the descriptors: **#F** and **1/q$_s$** for compounds in data set **DS3**

compounds	pK_a_Experimental	pK_a_predicted	error
**1**	3.69	3.22	0.47
**2**	1.70	2.15	0.45
**3**	0.97	1.44	0.47
**4**	2.05	2.05	0.00
**5**	3.68	3.74	0.06
**6**	2.43	2.23	0.20
**7**	1.33	1.07	0.26
**8**	1.49	1.83	0.34
**9**	0.94	0.57	0.37
**22**	3.08	2.71	0.37
**21**	2.43	2.77	0.34
**R^2**		0.89	
**RMSE**		0.11	
**MAE**		0.29	

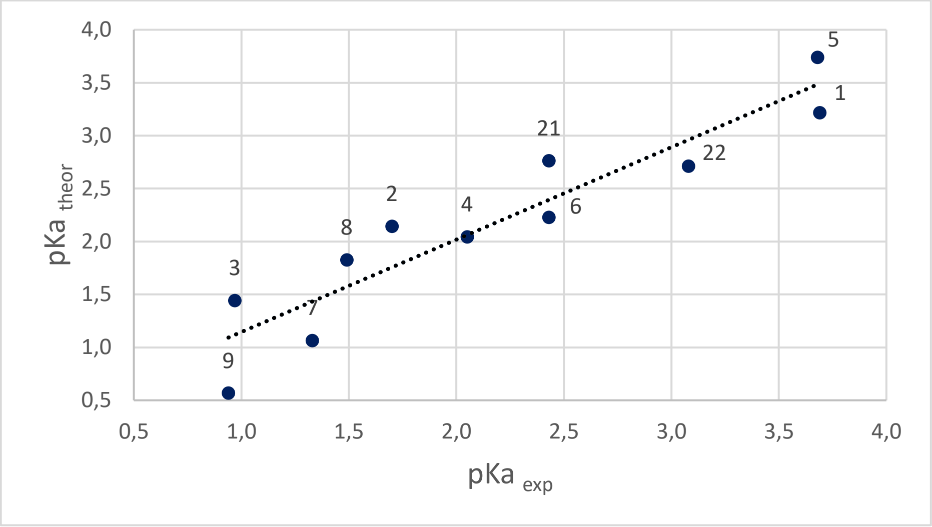

Figure S11. Experimental *vs*. the predicted pK_a values

15. Cartesian coordinates (in Angstroom) and potential energies (in Hartree) of the optimized structures.

Substrate 1
E= -685.847364 h

C	0.076883	-0.275028	0.000111
C	-0.887943	-1.300002	0.000254
C	-2.229130	-0.940020	0.000257
C	-2.572358	0.418210	0.000011
C	-1.541557	1.352460	-0.000239
N	-0.236144	1.022999	-0.000206
H	-2.998424	-1.705789	0.000446
H	-0.585377	-2.341991	0.000428
H	-3.607416	0.740883	-0.000085
H	-1.759672	2.417476	-0.000346
S	1.793649	-0.757453	-0.000407
C	2.644642	0.853120	0.000553
H	2.388039	1.423745	0.893462
H	2.388332	1.424538	-0.891930
H	3.711231	0.620612	0.000681

Substrate 2
E= -884.326560 h

C	0.479663	-0.268681	-0.539760
C	1.441440	-0.070169	-1.540407
C	2.777990	-0.246292	-1.196611
C	3.105538	-0.597890	0.117050
C	2.069483	-0.761870	1.032471
N	0.771438	-0.609917	0.713943
H	3.553571	-0.104943	-1.942187
H	1.149960	0.215103	-2.545260
H	4.135412	-0.741091	0.423466
H	2.274942	-1.032322	2.064427
S	-1.244182	-0.058133	-1.001671
C	-2.015348	-0.129243	0.643642
H	-1.382154	0.304381	1.414970
F	-3.207213	0.545610	0.567605
F	-2.326998	-1.417113	1.006657

Substrate 3
E= -983.572316 h

C	-2.313873	0.080414	-0.041860
C	-0.917035	0.074695	-0.055521
C	-0.259811	1.305082	0.004733
C	-1.021216	2.470896	0.067539
C	-2.414348	2.361591	0.064380
N	-3.059538	1.187122	0.007533
H	0.824244	1.347009	-0.000462
H	-0.369577	-0.858285	-0.108898
H	-0.552870	3.447755	0.113342
H	-3.039487	3.249067	0.105146
S	-3.205379	-1.489377	-0.160370
C	-3.347692	-1.858263	1.613823
F	-3.992631	-3.037910	1.746785
F	-2.151427	-1.974106	2.236958
F	-4.046980	-0.929970	2.305329

Substrate 4
E= -923.657458 h

N	1.132767	1.098349	-0.264431
C	0.782202	-0.191649	-0.230612
C	1.683414	-1.236031	0.000318
C	3.020086	-0.911017	0.233579
C	3.400314	0.430543	0.208095
C	2.424013	1.394842	-0.050642
S	-0.952419	-0.560204	-0.562927
C	-1.553097	-0.844117	1.143509
C	-3.031253	-1.137702	1.174578
F	-0.851766	-1.882239	1.735013
F	-1.267346	0.253159	1.937201
H	3.748345	-1.692637	0.423345
H	1.343673	-2.264117	-0.001913
H	4.428892	0.728827	0.378149
H	2.684196	2.449128	-0.087469
H	-3.589648	-0.293214	0.767082
H	-3.329970	-1.302886	2.212634
H	-3.247533	-2.033365	0.589559

Substrate 5
E= -725.171022 h

C	-0.286615	-0.400402	-0.087712
C	-1.370450	-1.223039	0.271204
C	-2.636685	-0.655096	0.331600
C	-2.789189	0.706785	0.043369
C	-1.651540	1.433235	-0.295770
N	-0.419362	0.898748	-0.365120
H	-3.493781	-1.263046	0.603987
H	-1.213492	-2.273032	0.494887
H	-3.759161	1.189659	0.083063
H	-1.719579	2.493883	-0.524796
S	1.325947	-1.158814	-0.193552
C	2.425293	0.286093	-0.455633
H	1.974209	0.902418	-1.235351
H	3.345226	-0.153457	-0.850964
C	2.692151	1.093331	0.810857
H	3.372035	1.922895	0.584109
H	1.763270	1.512117	1.205637
H	3.153859	0.472903	1.584605

Substrate 6

E= -923.661384 h

C	1.097912	0.457440	-0.099734
N	0.947541	-0.849111	-0.319071
C	2.026514	-1.638829	-0.166475
C	3.278098	-1.161929	0.210594
C	3.418447	0.211982	0.440913
C	2.315485	1.043099	0.285181
H	2.387776	2.112503	0.452112
H	4.375958	0.628890	0.736210
H	4.114585	-1.842448	0.321737
H	1.867849	-2.697410	-0.354126
S	-0.315645	1.538165	-0.297399
C	-1.585055	0.365148	-0.874912
C	-2.169855	-0.471406	0.241253
F	-2.894429	0.318446	1.115004
F	-3.075350	-1.369362	-0.302610
H	-1.439863	-1.037052	0.819039
H	-2.375071	0.965995	-1.331596
H	-1.146760	-0.296615	-1.622765

Substrate 7

E= -1122.130775 h

N	-2.569398	-1.225216	0.742959
C	-1.619453	-0.950883	-0.152598
C	-1.563299	0.232463	-0.894768
C	-2.574290	1.172622	-0.693863
C	-3.567855	0.909341	0.249800
C	-3.514953	-0.296081	0.951047
S	-0.369244	-2.205508	-0.488276
C	0.081487	-2.709287	1.211101
C	-0.956335	-3.574118	1.951308
F	-0.441177	-3.921470	3.173843
F	1.241423	-3.423184	1.092436
F	0.343986	-1.625786	2.002187
F	-1.151839	-4.731307	1.248804
H	-2.572924	2.101341	-1.254758
H	-0.754967	0.411762	-1.593890
H	-4.364087	1.618918	0.443850
H	-4.262641	-0.537383	1.701212
H	-1.901932	-3.048051	2.075751

Substrate 8

E= -1022.912787 h

C	-1.670425	-0.064109	0.058574
C	-0.632129	0.379110	-0.775714
C	-0.356743	1.741847	-0.804589
C	-1.110243	2.608853	-0.005203
C	-2.114043	2.062337	0.789457
N	-2.396557	0.747833	0.822416
H	0.438572	2.122246	-1.437440
H	-0.063045	-0.323644	-1.374848
H	-0.922946	3.676553	0.003896
H	-2.723910	2.694398	1.429294
S	-2.037534	-1.822735	0.037983
C	-3.219532	-1.965729	1.412235
C	-2.574624	-2.349933	2.726393
F	-1.642195	-1.459371	3.142177
F	-3.512336	-2.427356	3.709536
F	-1.963248	-3.561733	2.675759
H	-3.721683	-1.004642	1.537364
H	-3.947138	-2.735030	1.150727

Substrate 9

E= -1221.375052 h

C	-2.170725	0.052998	-0.059193
C	-0.810586	0.157667	-0.360872
C	-0.224204	1.422463	-0.290196
C	-1.011756	2.508435	0.088845
C	-2.362571	2.288497	0.370869
N	-2.944977	1.083633	0.285187
H	0.828720	1.550322	-0.518025
H	-0.234791	-0.719936	-0.628785
H	-0.596263	3.507174	0.162993
H	-3.007869	3.111169	0.665791
S	-2.964413	-1.562533	-0.231707
C	-3.345534	-1.977538	1.501687
F	-4.016970	-3.165321	1.458758
F	-4.156869	-1.066397	2.105889
C	-2.131234	-2.184326	2.450935
F	-1.456828	-1.034611	2.621595
F	-2.556723	-2.604676	3.657432
F	-1.290662	-3.107902	1.958320

Substrate 10

E= -836.251665 h

C	0.358918	-0.206551	0.048720
C	1.150115	-1.305034	-0.266646
C	2.532946	-1.100179	-0.274608
C	3.029812	0.164212	0.038196
C	2.128986	1.188310	0.349570
N	0.800904	1.010517	0.352330
H	3.204957	-1.916300	-0.517034
H	0.703847	-2.265721	-0.490576
H	4.095891	0.360959	0.046186
H	2.482677	2.182998	0.603536
S	-1.448915	-0.371452	0.046715
C	-1.925226	0.111968	1.709643
H	-1.573577	1.128702	1.882664
H	-1.477353	-0.590500	2.412944
H	-3.014805	0.059710	1.743856
O	-2.011319	0.612734	-0.906859
O	-1.765127	-1.804773	-0.153913

Substrate 11

E= -824.410745 h

C	0.630120	-0.808475	-0.005975
N	0.475087	0.287925	-0.750535
C	1.583912	0.937594	-1.149974
C	2.873606	0.526777	-0.827723
C	3.021147	-0.626537	-0.047093
C	1.887644	-1.311685	0.372328
H	1.964153	-2.209948	0.975875
H	4.007400	-0.986185	0.228598
H	3.732933	1.088002	-1.176928
H	1.419170	1.824918	-1.755796
S	-0.817195	-1.693242	0.558311
C	-2.167983	-0.733919	-0.216825
C	-2.371454	-1.015877	-1.689656
F	-2.807340	-2.352595	-1.874922
H	-1.451773	-0.894105	-2.263312
H	-3.066557	-0.991809	0.349333
H	-1.959311	0.329285	-0.080934
H	-3.156702	-0.370398	-2.094439

Substrate 12

E= -1034.715272 h

C	-2.178152	0.033877	-0.030492
C	-0.835617	0.104903	-0.379695
C	-0.247555	1.371975	-0.319780
C	-1.020060	2.456322	0.093330
C	-2.361098	2.247960	0.436080
N	-2.944073	1.043907	0.371230
H	0.795404	1.502802	-0.586340
H	-0.285919	-0.778707	-0.678677
H	-0.596358	3.452114	0.156203
H	-2.986292	3.069600	0.771023
S	-3.044151	-1.550685	-0.088227
C	-3.631871	-1.718998	1.687507
H	-4.301292	-0.899850	1.951424
F	-4.262012	-2.913919	1.776910
F	-2.543140	-1.735174	2.493637
O	-2.066610	-2.621479	-0.349952
O	-4.267454	-1.443753	-0.902857

Substrate 13

E= -1133.958819 h

C	-2.254337	0.097188	-0.050220
C	-0.883168	0.093734	-0.279443
C	-0.244171	1.335744	-0.218593
C	-0.999922	2.469859	0.071999
C	-2.376133	2.335078	0.292777
N	-3.006089	1.155533	0.230224
H	0.823435	1.408352	-0.393584
H	-0.350404	-0.823608	-0.494968
H	-0.538187	3.448892	0.130022
H	-2.991332	3.198719	0.524713
S	-3.157438	-1.468626	-0.119327
C	-3.496852	-1.834806	1.711826
F	-3.993877	-3.072476	1.804538
F	-2.354037	-1.761359	2.404767
F	-4.375461	-0.964731	2.215187
O	-2.240476	-2.529462	-0.560227
O	-4.475160	-1.290668	-0.740183

Substrate 14

E= -785.084560 h

C	0.534622	-0.834484	-0.445002
C	1.697107	-1.469519	0.017620
C	2.817956	-0.681749	0.258486
C	2.745373	0.696582	0.030190
C	1.542282	1.222883	-0.432929
N	0.450108	0.474787	-0.670179
H	3.735409	-1.136438	0.617991
H	1.715773	-2.541608	0.181833
H	3.597064	1.344430	0.203745
H	1.439248	2.287034	-0.627508
S	-0.909054	-1.868603	-0.726885
C	-2.100759	-0.678558	-1.359373
H	-1.970392	0.282306	-0.864157
F	-1.944536	-0.462616	-2.744918
H	-3.093736	-1.100529	-1.202664

Substrate 15

E= -875.575820 h

C	0.733620	-0.257868	-0.029541
C	1.690493	-1.213197	0.293749
C	3.024292	-0.794942	0.282490
C	3.314284	0.525721	-0.056867
C	2.261558	1.390639	-0.374419
N	0.977618	1.007771	-0.358969
H	3.817900	-1.491342	0.530855
H	1.401249	-2.227259	0.539217
H	4.336272	0.886807	-0.080472
H	2.453337	2.423604	-0.648855
S	-1.025185	-0.706845	-0.014929
C	-1.554417	-0.402459	-1.722904
H	-0.927851	-1.052101	-2.339190
H	-1.299442	0.640459	-1.923157
C	-3.042681	-0.690553	-1.892500
H	-3.317010	-0.499840	-2.933314
H	-3.274482	-1.733460	-1.663040
H	-3.647093	-0.042209	-1.253976
O	-1.746499	0.226928	0.882655
O	-1.111177	-2.158859	0.274648

Substrate 16

E= -1074.058363 h

C	-1.047285	-0.325052	0.135312
N	-1.354239	0.335070	1.246666
C	-2.484384	1.054040	1.226379
C	-3.320285	1.116652	0.105968
C	-2.966278	0.411122	-1.043058
C	-1.784839	-0.336177	-1.041761
H	-1.448698	-0.894263	-1.906498
H	-3.591364	0.442741	-1.928756
H	-4.225942	1.711473	0.140632
H	-2.727595	1.596642	2.134737
S	0.483029	-1.289853	0.243059
O	0.795068	-1.803419	-1.108071
O	0.376006	-2.236595	1.371057
C	1.752586	-0.072272	0.704818
C	2.149001	0.834060	-0.441760
F	3.180602	1.642770	-0.018892
F	1.101936	1.667523	-0.775147
H	2.466404	0.312586	-1.345174
H	1.365614	0.500298	1.550064
H	2.608907	-0.674440	1.017975

Substrate 17

E= -1173.307324 h

C	-1.150916	-0.357117	0.025909
N	-1.417963	0.192275	1.205424
C	-2.533204	0.929967	1.286883
C	-3.393873	1.119099	0.200015
C	-3.081564	0.526551	-1.022622
C	-1.915524	-0.237493	-1.127409
H	-1.611205	-0.709842	-2.052903
H	-3.726684	0.658341	-1.884318
H	-4.286298	1.723273	0.316953
H	-2.742865	1.383011	2.250862
S	0.355743	-1.363348	0.000108
O	0.682266	-1.670740	-1.404511
O	0.219661	-2.465541	0.971907
C	1.623953	-0.259562	0.713429
C	2.084785	0.872466	-0.182238
F	2.895522	1.704566	0.515661
F	1.052200	1.616977	-0.647470
F	2.782850	0.441591	-1.254222
H	1.192375	0.146257	1.629740
H	2.469121	-0.907254	0.953772

Substrate 18

E= -1074.051737 h

C	-1.382532	-0.203359	0.242513
C	-2.384650	-0.787014	-0.523973
C	-3.561862	-0.048668	-0.675442
C	-3.654440	1.205000	-0.073450
C	-2.567675	1.686857	0.665102
N	-1.436219	0.988761	0.827575
H	-4.385861	-0.448762	-1.255937
H	-2.249483	-1.763046	-0.972152
H	-4.550241	1.807612	-0.171476
H	-2.605356	2.661431	1.141876
S	0.171998	-1.101889	0.495954
C	1.417772	-0.127606	-0.555803
F	0.826511	0.018908	-1.779723
F	2.467944	-0.983735	-0.718243
C	1.843054	1.181450	0.033521
H	0.975225	1.828777	0.165084
O	0.035613	-2.432705	-0.125339
O	0.610306	-0.976979	1.897939
H	2.315891	1.010732	1.001370
H	2.557066	1.647761	-0.650345

Substrate 19

E= -1272.516644 h

C	1.405838	-0.461092	0.169278
C	1.730271	0.592424	1.013337
C	2.921089	1.267129	0.725911
C	3.679871	0.863203	-0.371364
C	3.231585	-0.206098	-1.155701
N	2.101931	-0.872273	-0.885276
H	3.241616	2.095560	1.347810
H	1.090868	0.866719	1.842820
H	4.605153	1.367031	-0.626115
H	3.792515	-0.538517	-2.023262
S	-0.089716	-1.429849	0.475002
C	-1.070317	-1.168782	-1.136448
F	-1.037491	0.160176	-1.397356
F	-2.342551	-1.527334	-0.859921
C	-0.547256	-1.938273	-2.366364
H	0.513210	-1.759260	-2.545033
F	-0.777121	-3.267930	-2.186520
F	-1.279935	-1.529197	-3.446939
O	-0.879561	-0.773152	1.526877
O	0.232021	-2.861251	0.559283

Substrate 20

E= -1371.759880 h

C	-1.409352	0.464217	0.052384
C	-1.912673	-0.134552	1.201415
C	-3.084094	-0.881369	1.044675
C	-3.652244	-0.987186	-0.223459
C	-3.036464	-0.343109	-1.303590
N	-1.919485	0.382942	-1.170501
H	-3.536806	-1.370438	1.899971
H	-1.421047	-0.019404	2.158948
H	-4.558655	-1.559814	-0.383373
H	-3.451758	-0.410041	-2.304146
S	0.094182	1.465423	0.172792
C	1.391541	0.297551	-0.605808

F	1.264658	0.359568	-1.944403
F	1.124307	-0.958738	-0.190285
C	2.854555	0.655119	-0.221609
F	3.093837	0.363763	1.061814
F	3.702459	-0.050001	-0.987498
F	3.077931	1.963599	-0.425360
O	0.465897	1.599149	1.588791
O	0.015117	2.633282	-0.710646

Substrate 21

E= -974.810911 h

C	0.820937	-0.286251	0.029719
N	1.117906	0.327782	-1.111659
C	2.320323	0.913372	-1.187318
C	3.240971	0.889436	-0.133917
C	2.896614	0.239337	1.050014
C	1.641656	-0.369172	1.148173
H	1.312157	-0.880648	2.043737
H	3.586346	0.207072	1.886418
H	4.203077	1.376303	-0.246237
H	2.553614	1.417804	-2.120054
S	-0.808704	-1.086428	0.053087
O	-1.042067	-1.575451	1.430315
O	-0.881250	-2.055169	-1.065022
C	-1.939768	0.291209	-0.328137
C	-3.283174	-0.192076	-0.827174
F	-3.979200	-0.854344	0.206400
H	-3.886993	0.665221	-1.132885
H	-2.020984	0.891782	0.580128
H	-1.442432	0.869590	-1.111467
H	-3.181178	-0.896605	-1.654684

Substrate 22

E= -935.477382 h

C	0.688577	0.118448	0.070250
N	1.092384	-1.127595	0.297834
C	2.404734	-1.366150	0.175268
C	3.325388	-0.369045	-0.166426
C	2.865878	0.925809	-0.401368
C	1.497540	1.189606	-0.288400
H	1.077268	2.170785	-0.469687
H	3.554371	1.718556	-0.672807
H	4.378556	-0.612443	-0.248854
H	2.728931	-2.386336	0.356118
S	-1.094384	0.364442	0.273104
O	-1.516400	-0.078740	1.618239
O	-1.421048	1.729547	-0.184368
C	-1.821413	-0.851847	-0.889608
F	-1.431999	-0.531928	-2.175949
H	-1.446523	-1.839459	-0.619894
H	-2.905157	-0.763899	-0.790107

16.NMR spectra

^{1}H NMR (400 MHz, $CDCl_3$) of **2**

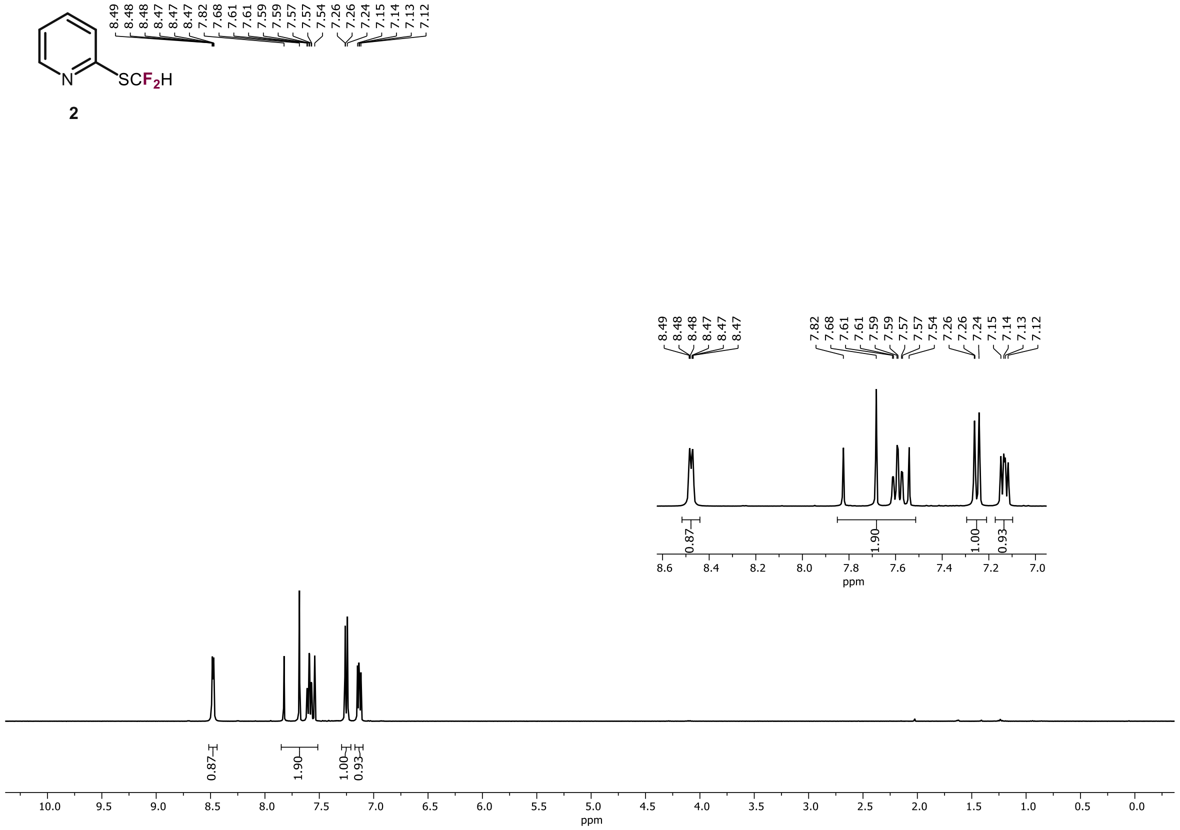

$^{13}C\{^{1}H\}$ NMR (100.6 MHz, $CDCl_3$) of **2**

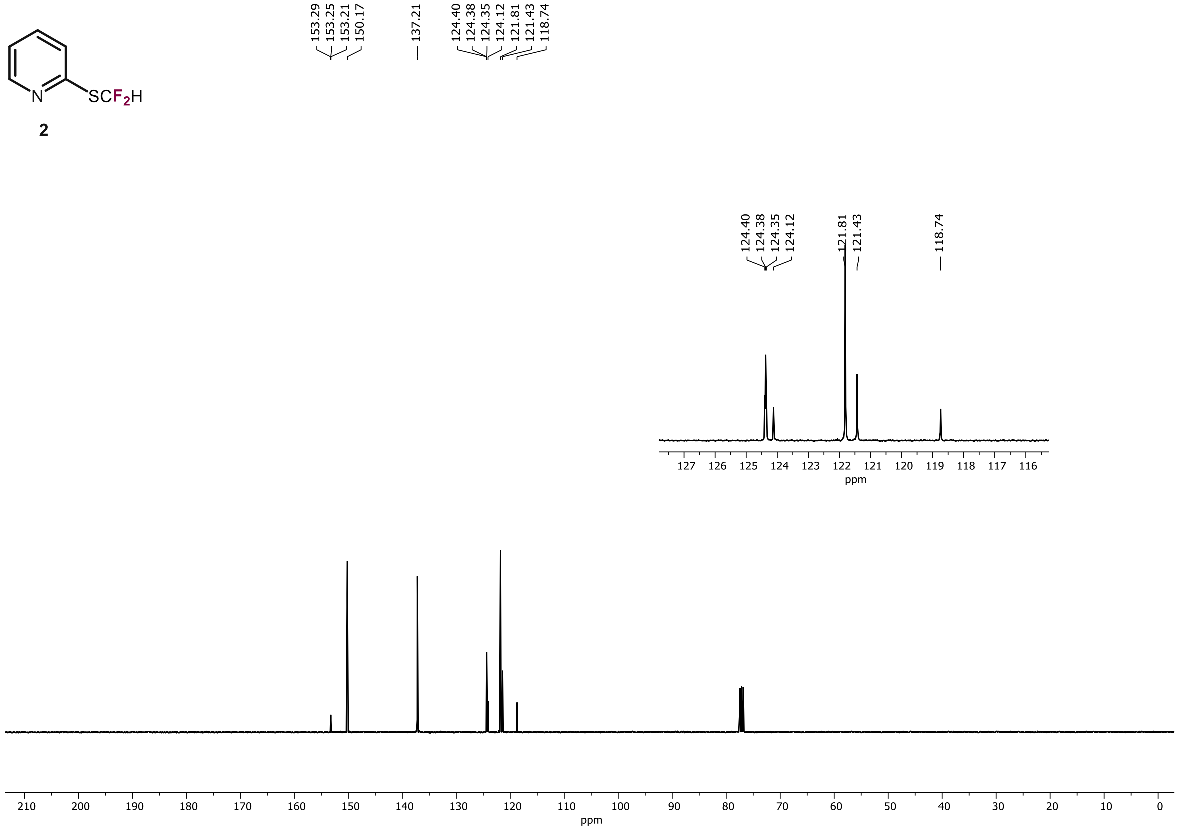

^{19}F NMR (376.5 MHz, $CDCl_3$) of **2**

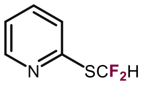

2

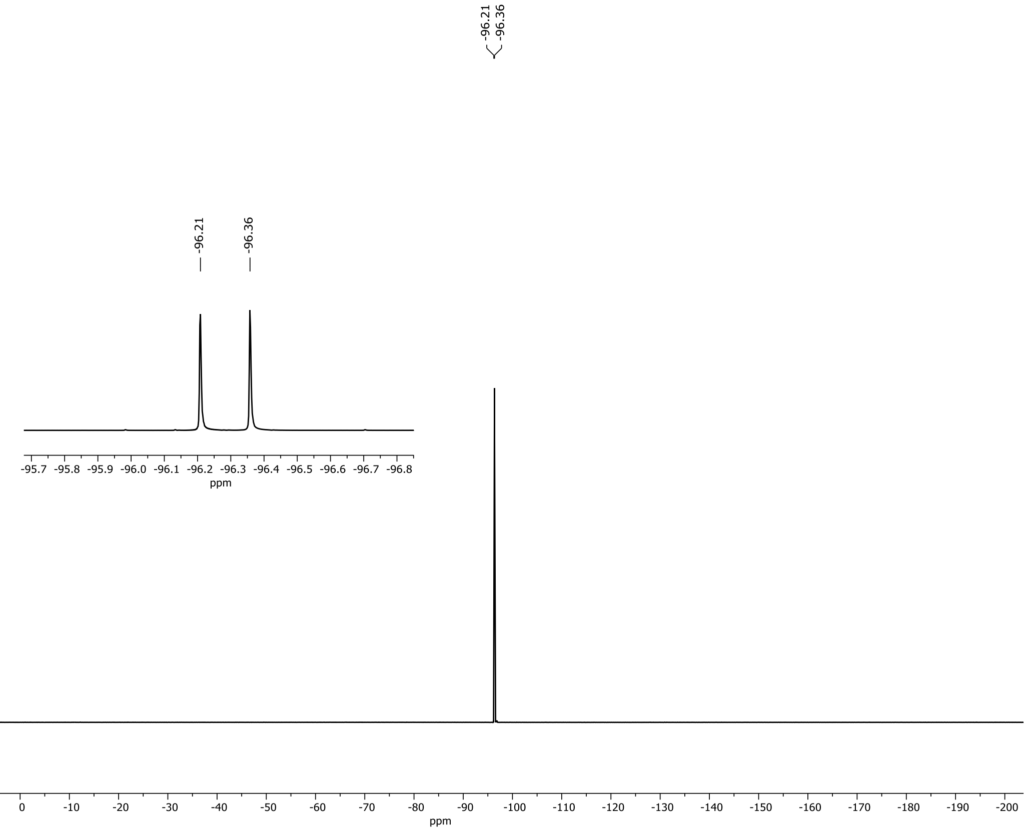

^{1}H NMR (400 MHz, $CDCl_3$) of **3**

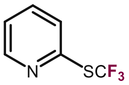

3

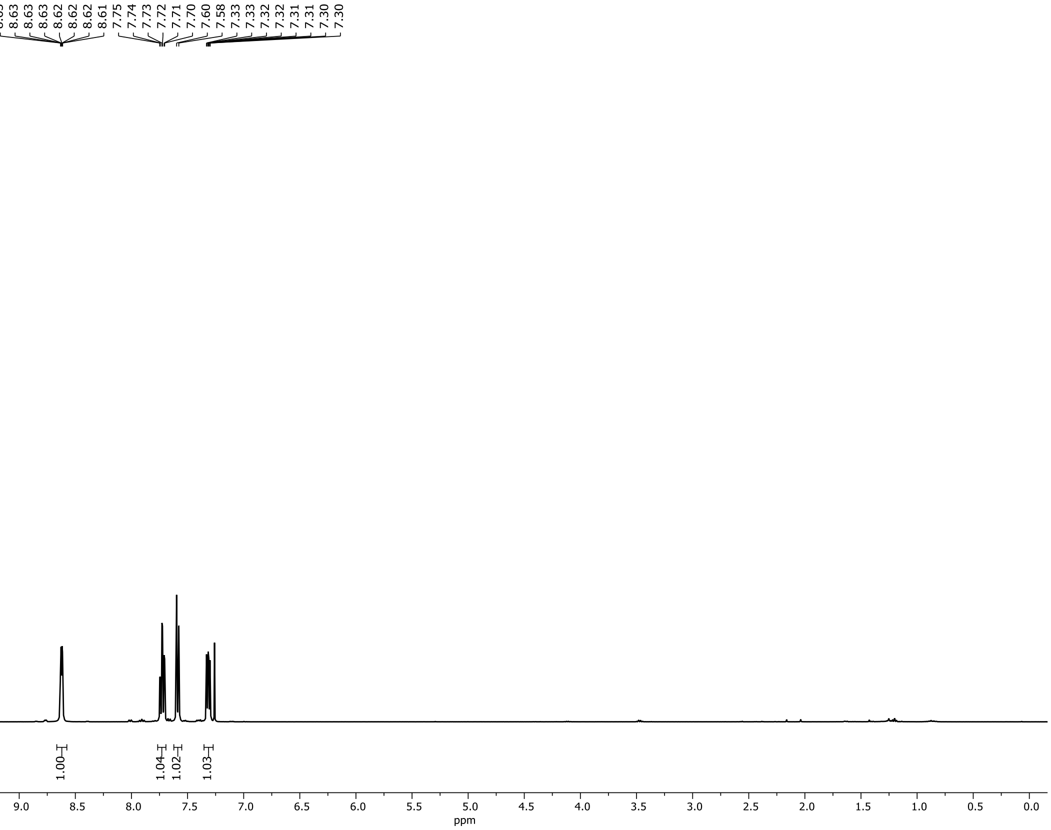

$^{13}C\{^1H\}$ NMR (100.6 MHz, $CDCl_3$) of **3**

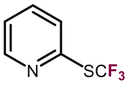

3

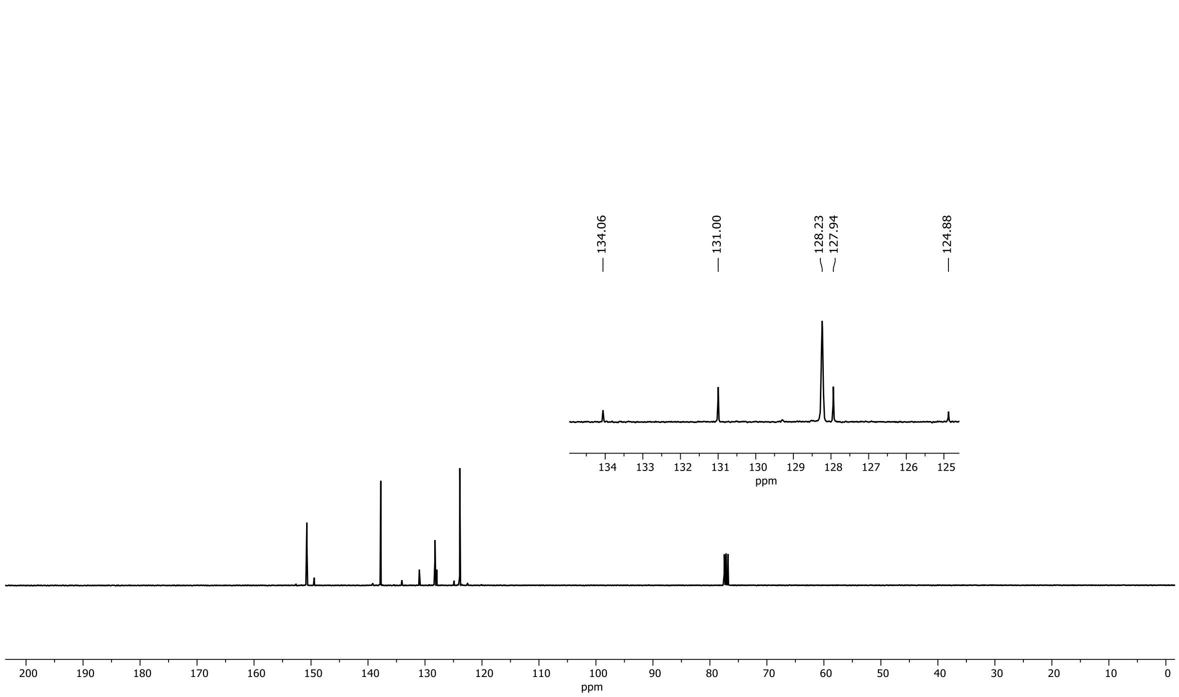

^{19}F NMR (376.5 MHz, $CDCl_3$) of **3**

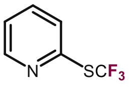

3

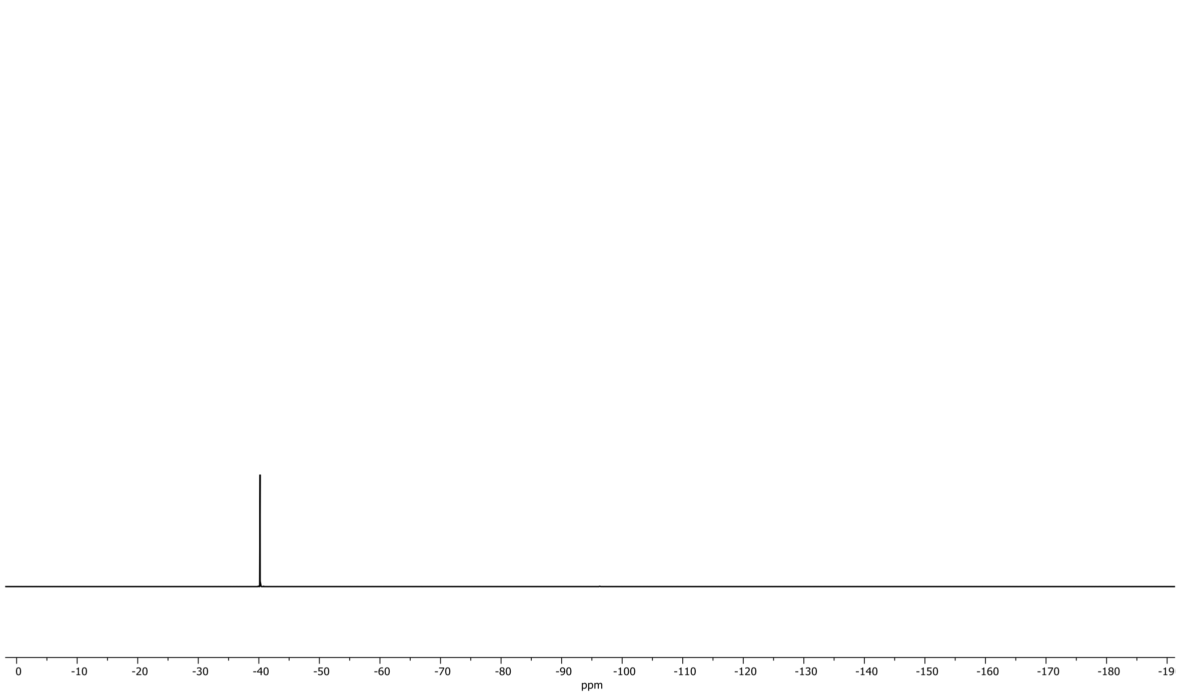

^{1}H NMR (400 MHz, $CDCl_3$) of **4**

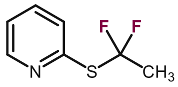

4

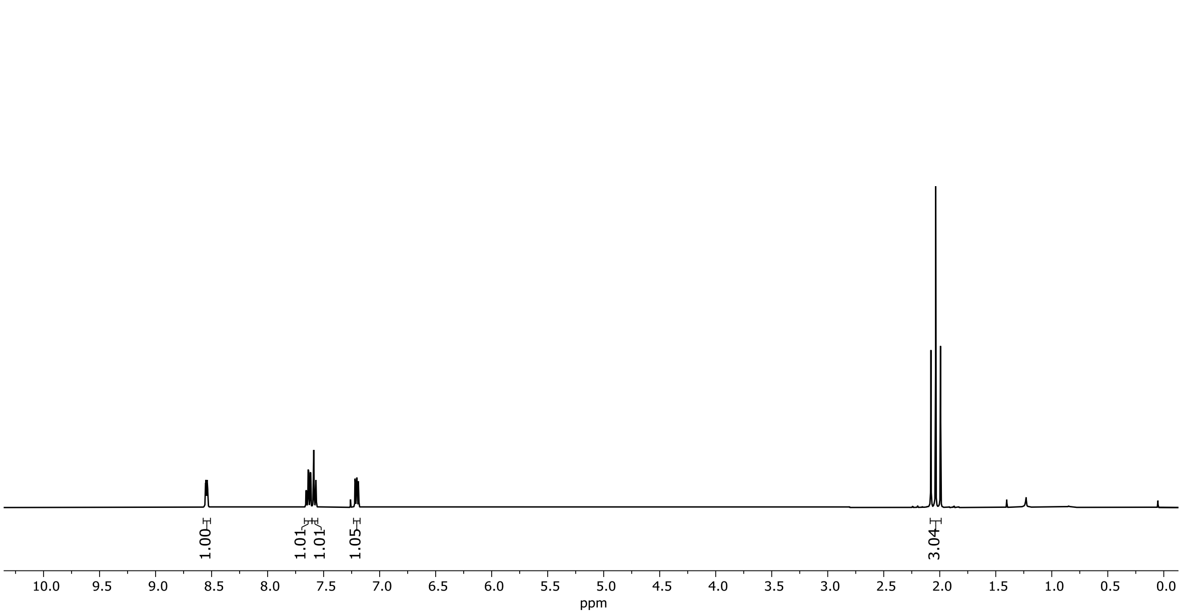

$^{13}C\{^{1}H\}$ NMR (100.6 MHz, $CDCl_3$) of **4**

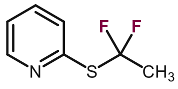

4

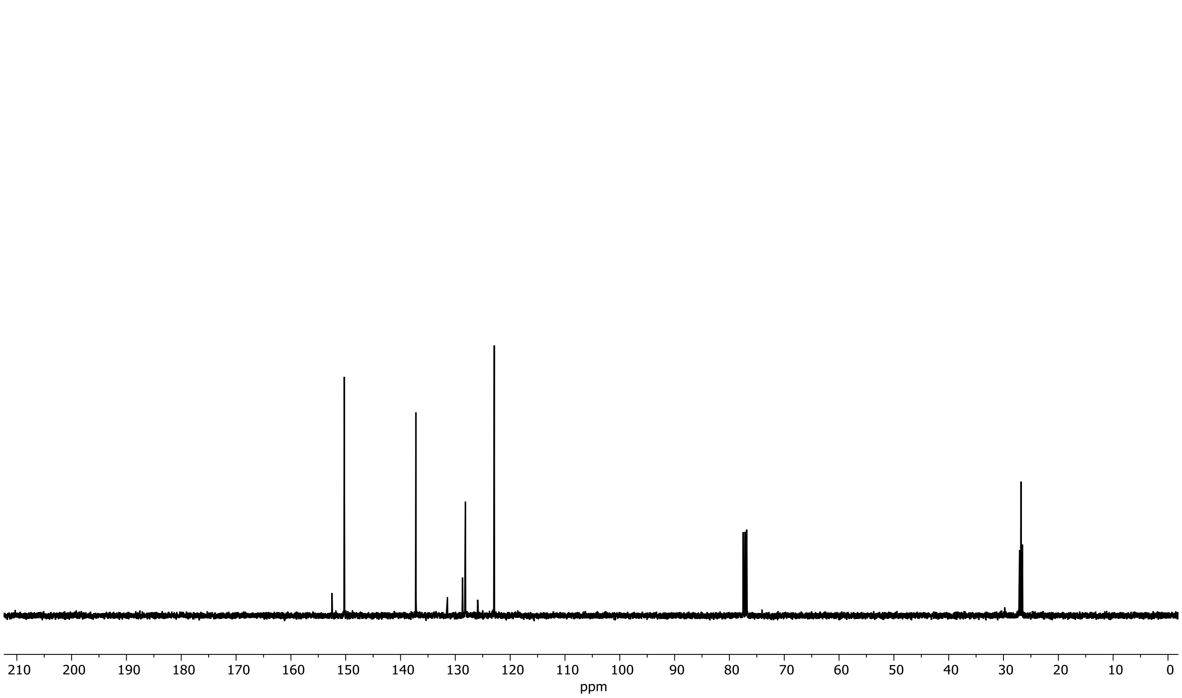

^{19}F NMR (376.5 MHz, $CDCl_3$) of **4**

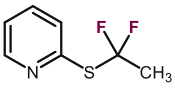

4

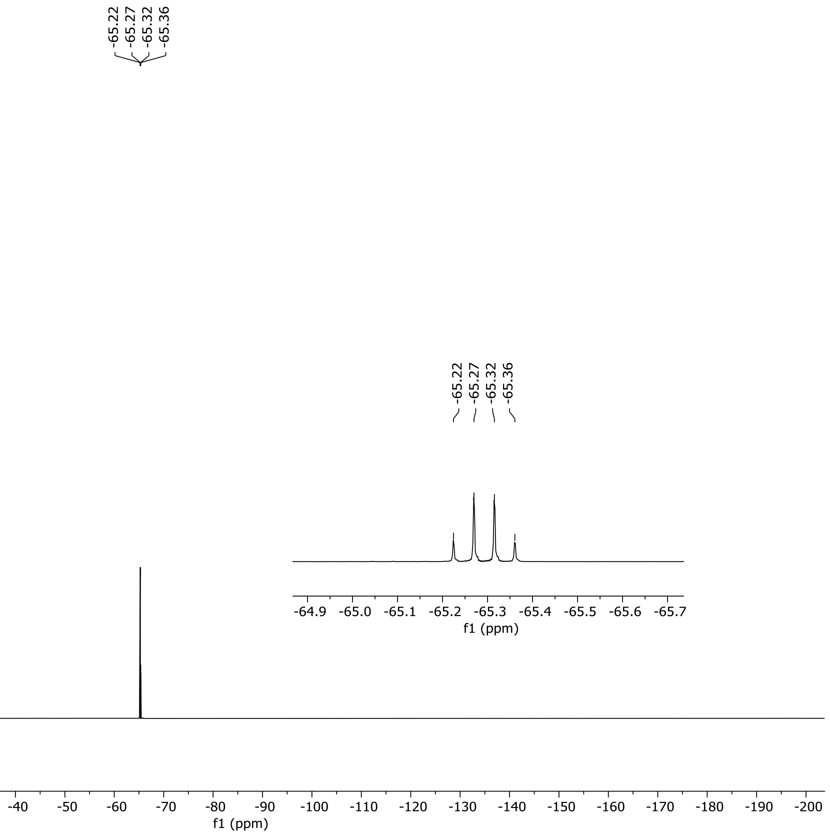

^{1}H NMR (400 MHz, $CDCl_3$) of **5**

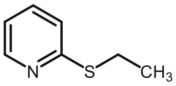

5

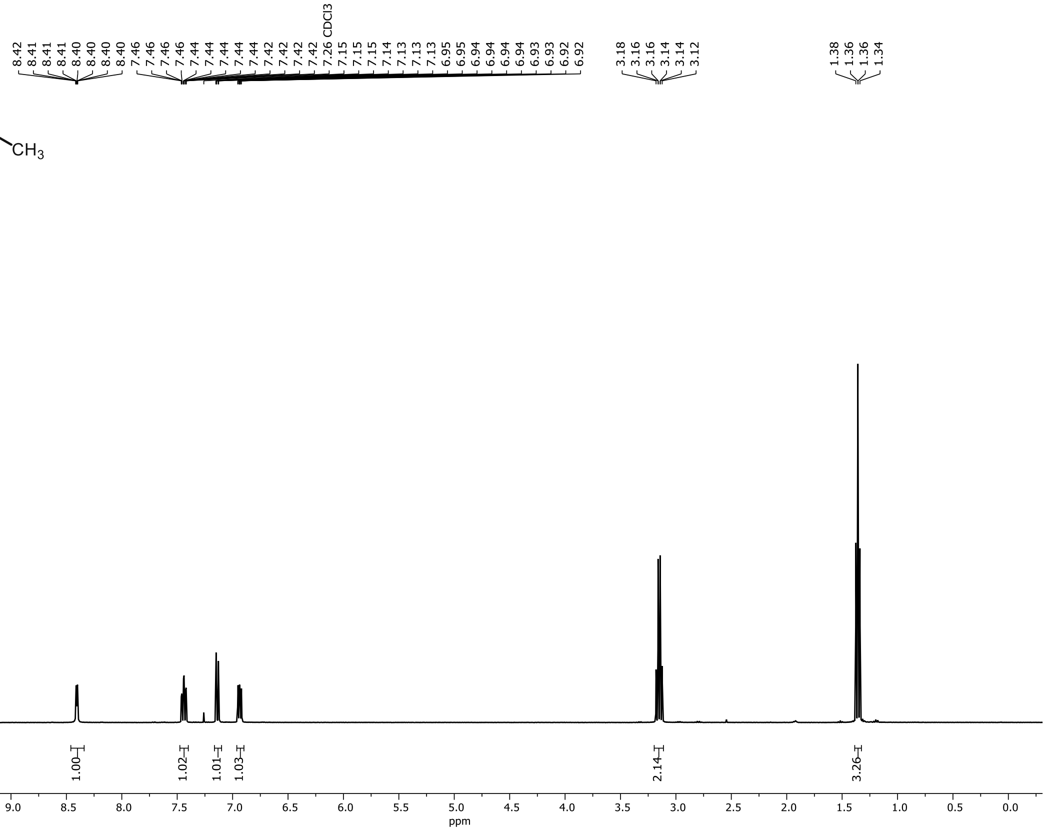

$^{13}C\{^{1}H\}$ NMR (100.6 MHz, $CDCl_3$) of **5**

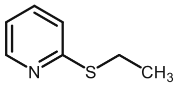

5

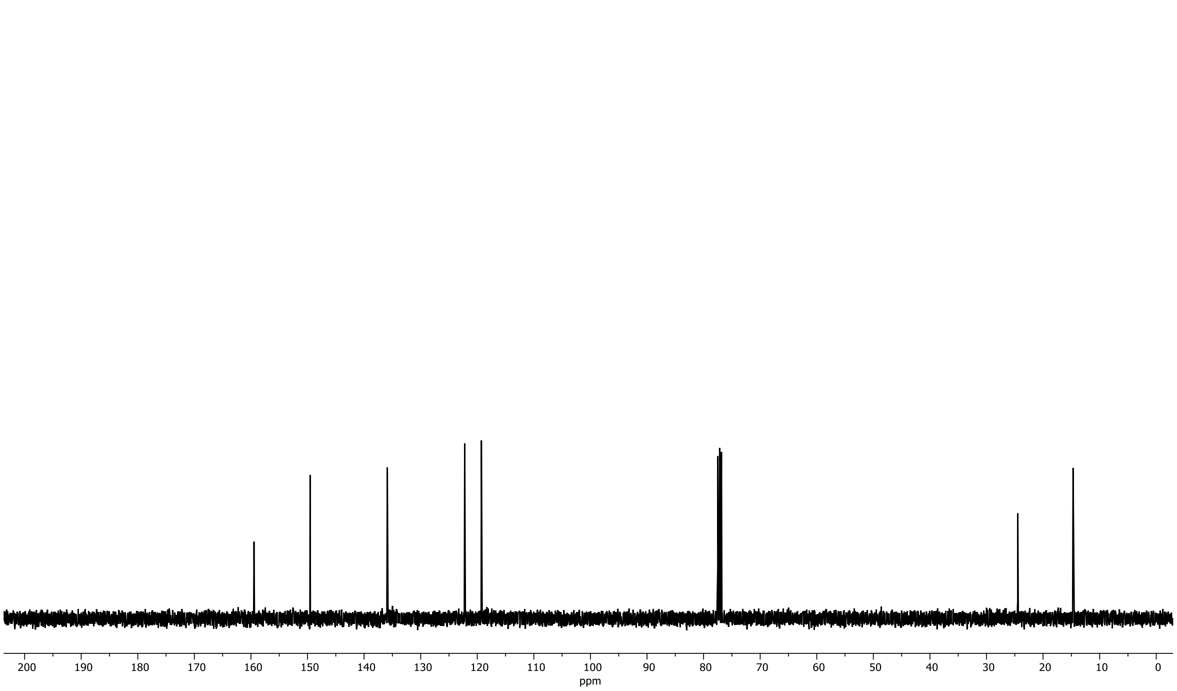

^{1}H NMR (400 MHz, $CDCl_3$) of **6**

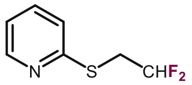

6

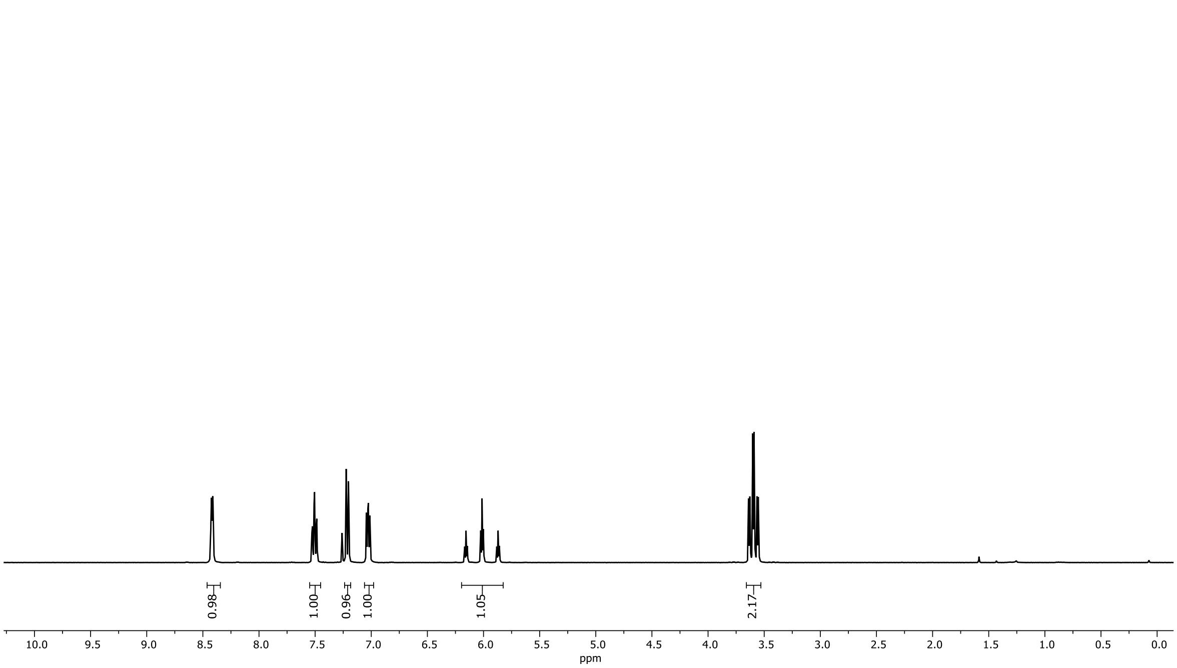

$^{13}C\{^{1}H\}$ NMR (100.6 MHz, $CDCl_3$) of **6**

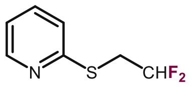

6

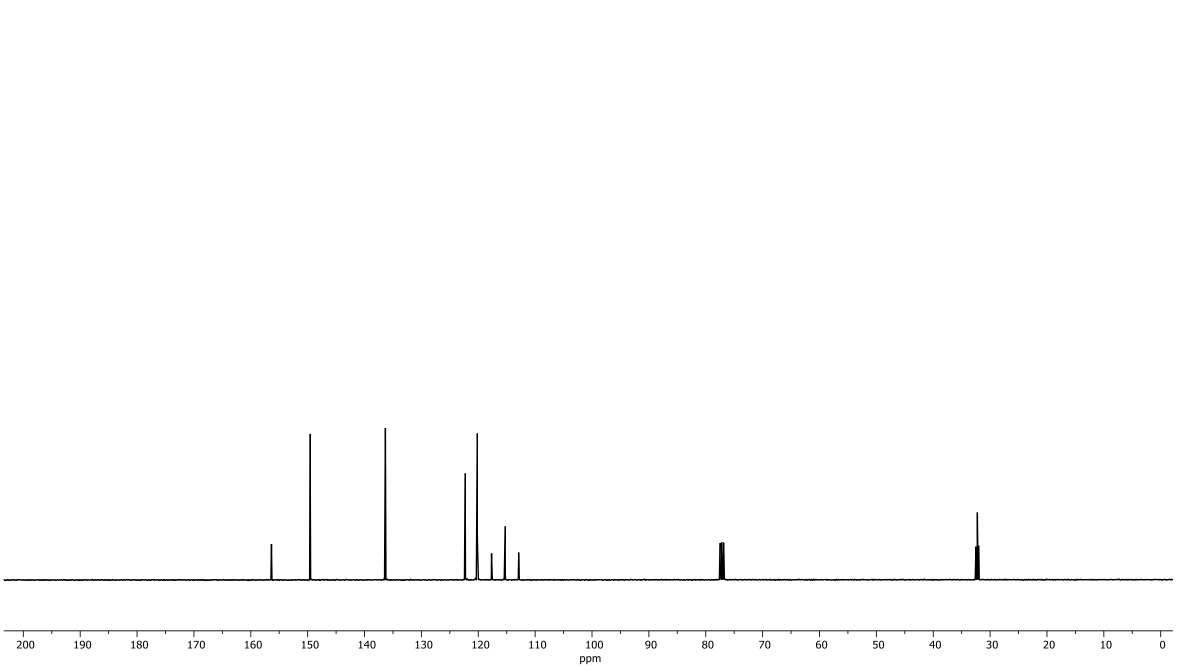

^{19}F NMR (376.5 MHz, $CDCl_3$) of **6**

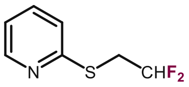

6

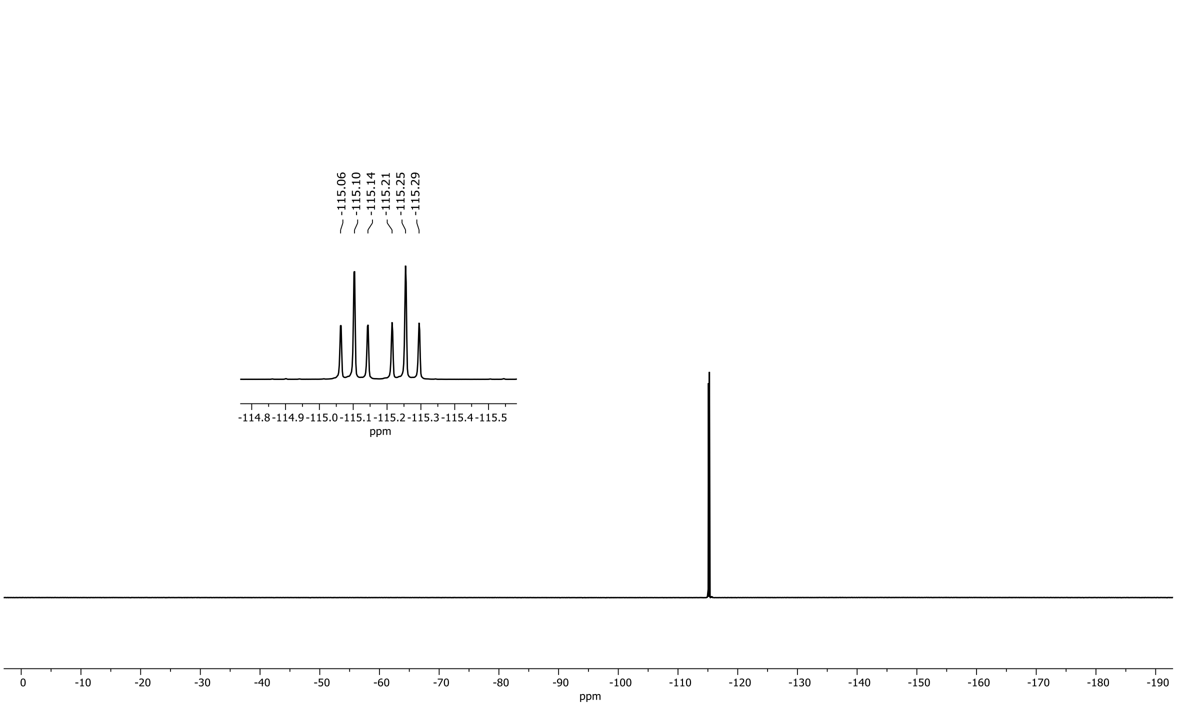

^{1}H NMR (400 MHz, $CDCl_3$) of **7**

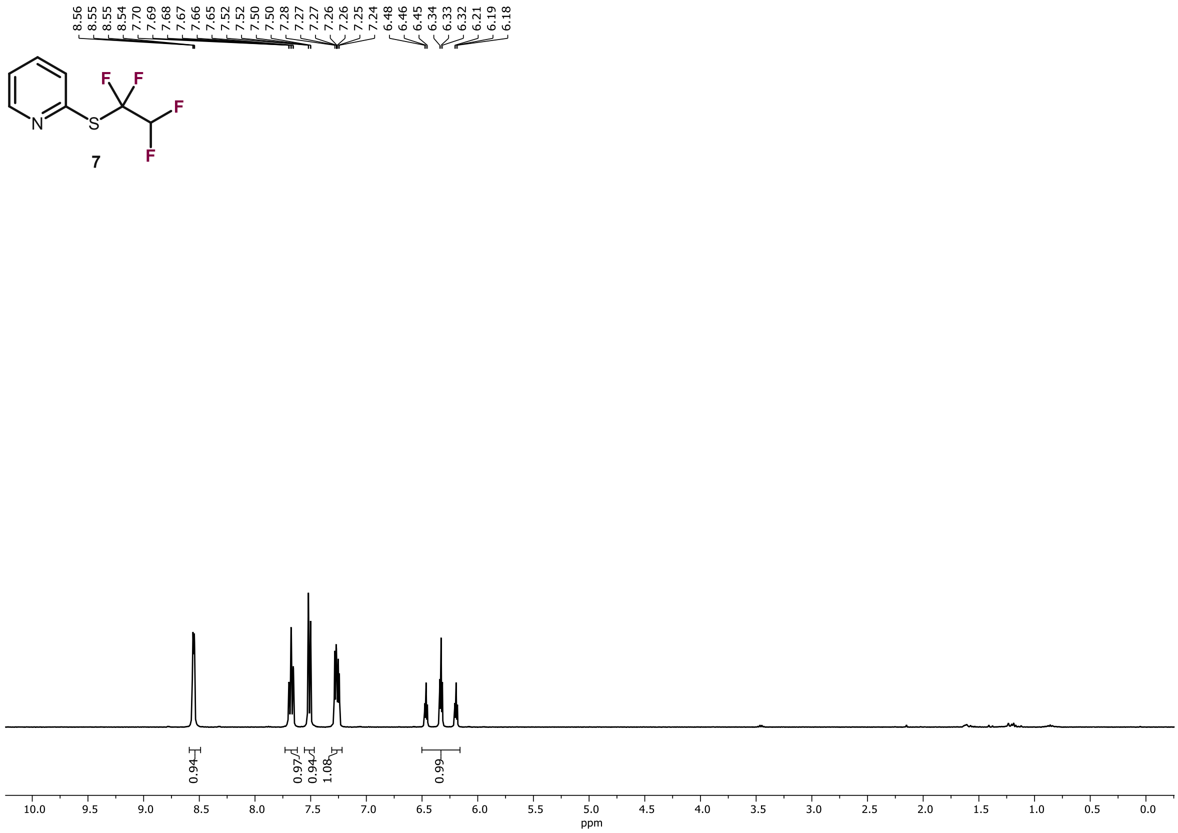

^{13}C{^{1}H} NMR (100.6 MHz, $CDCl_3$) of **7**

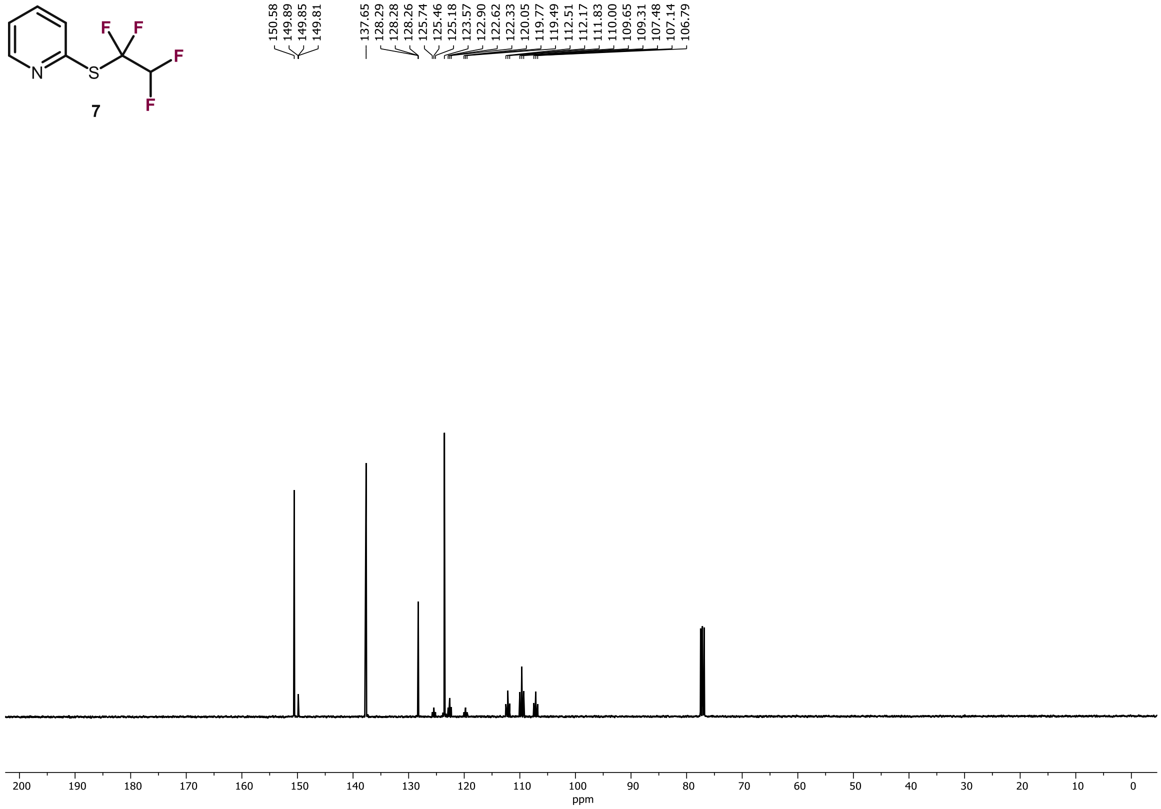

^{19}F NMR (376.5 MHz, $CDCl_3$) of **7**

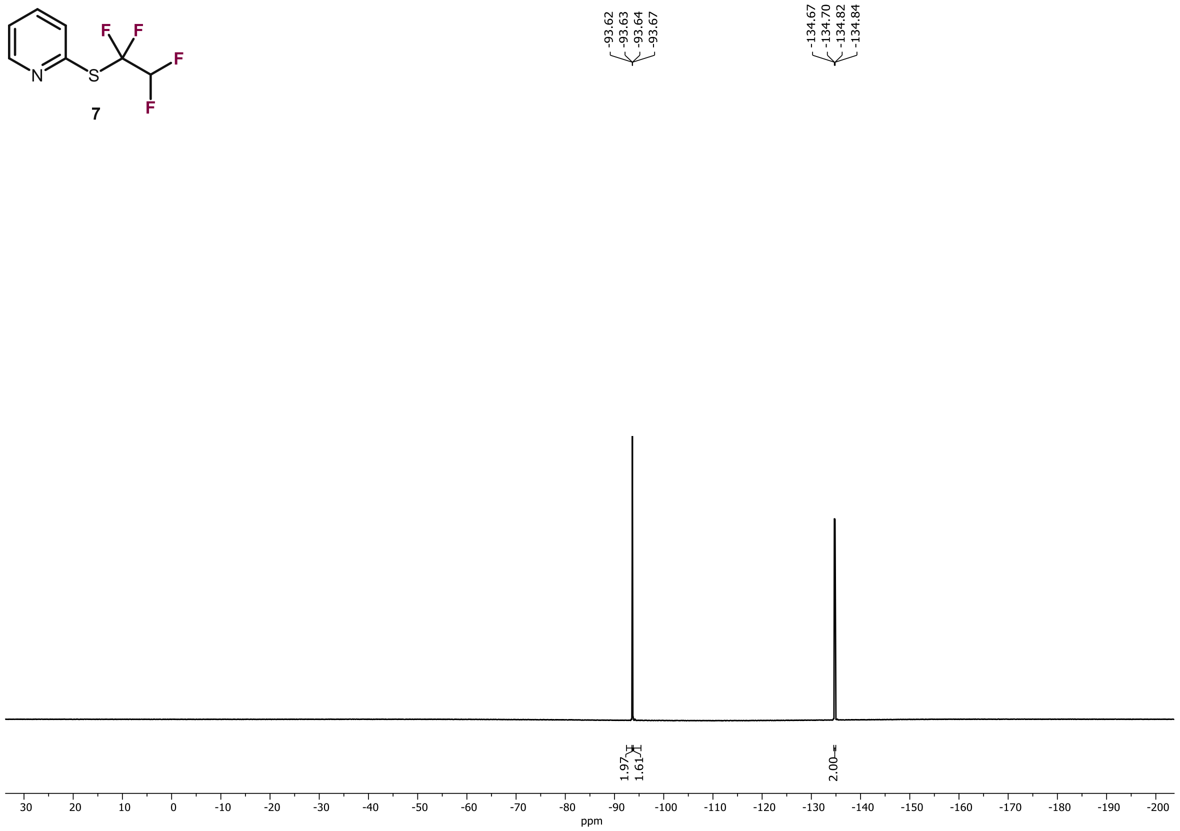

^{1}H NMR (400 MHz, $CDCl_3$) of **8**

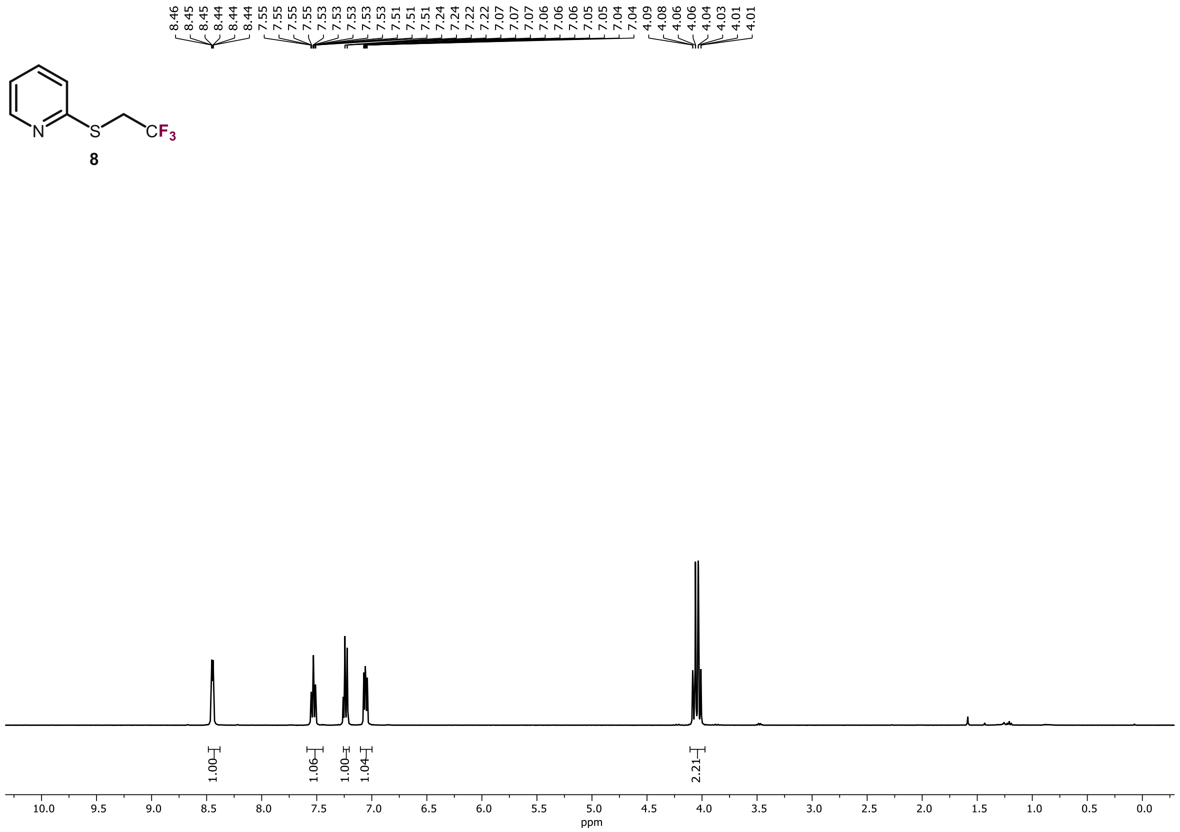

$^{13}C\{^{1}H\}$ NMR (100.6 MHz, $CDCl_3$) of **8**

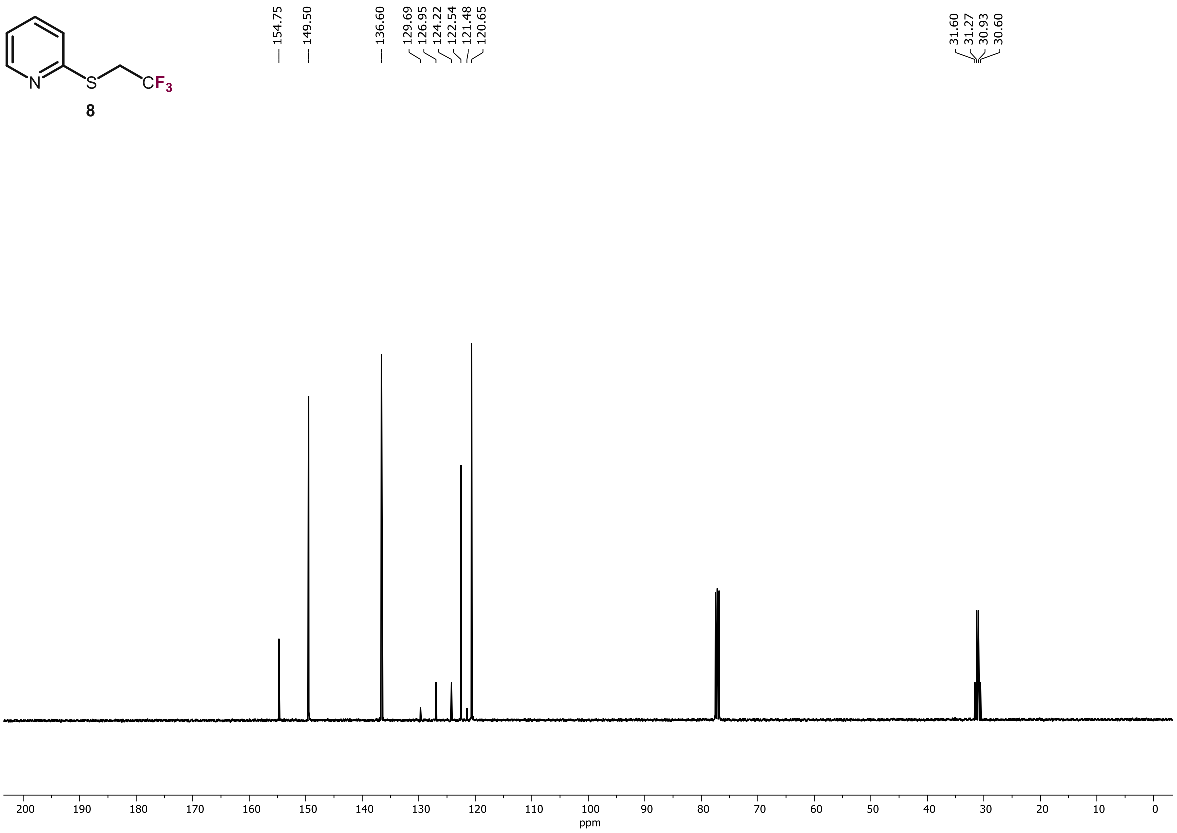

^{19}F NMR (376.5 MHz, $CDCl_3$) of **8**

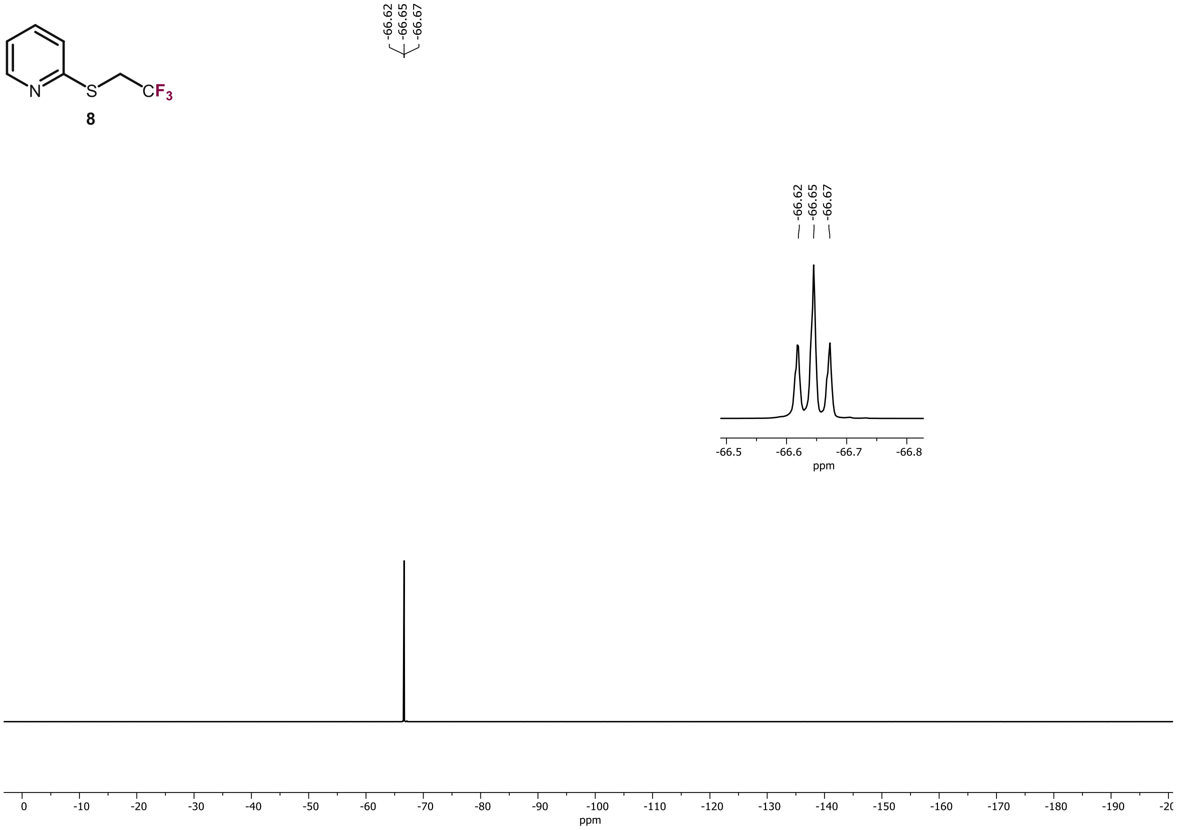

^{1}H NMR (400 MHz, $CDCl_3$) of **9**

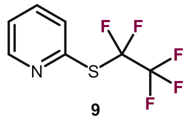

9

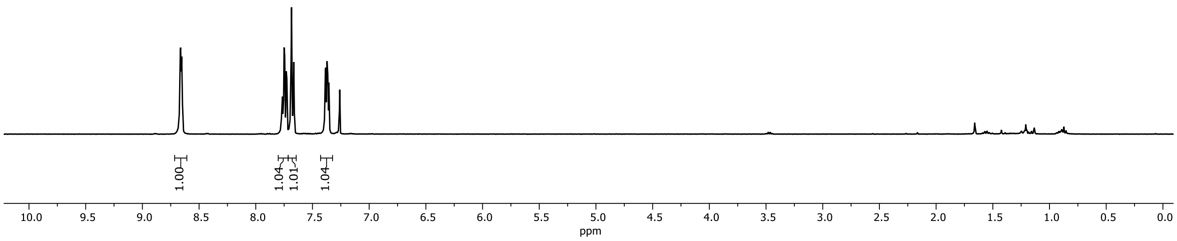

^{13}C{^{1}H} NMR (100.6 MHz, $CDCl_3$) of **9**

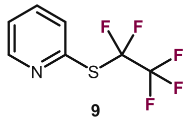

9

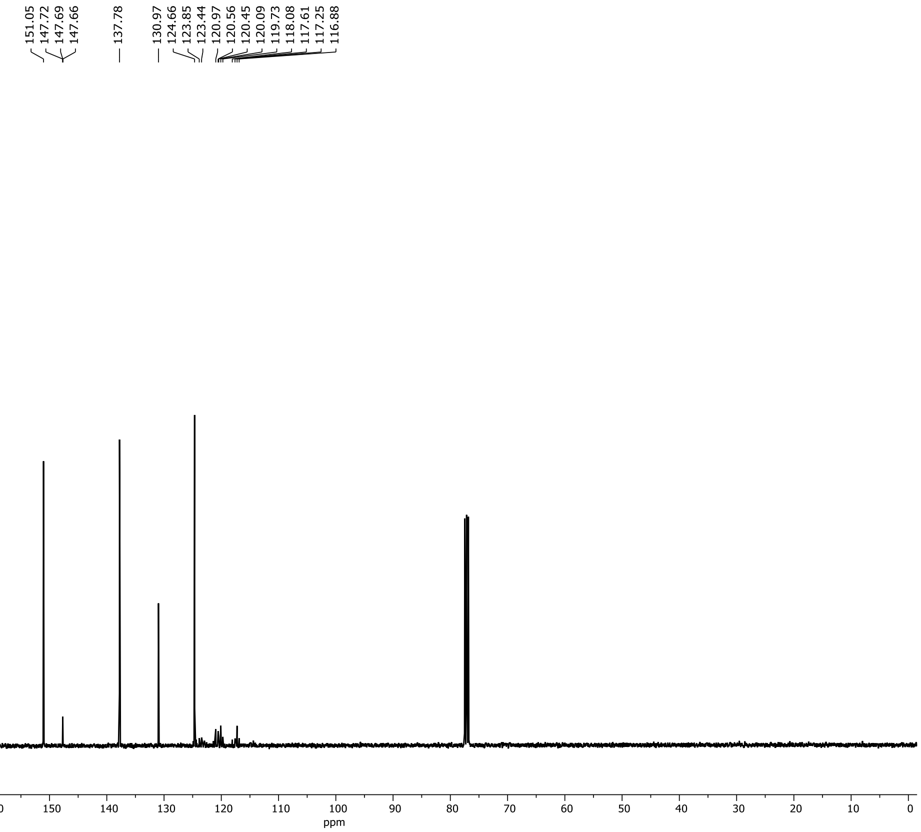

^{19}F NMR (376.5 MHz, $CDCl_3$) of **9**

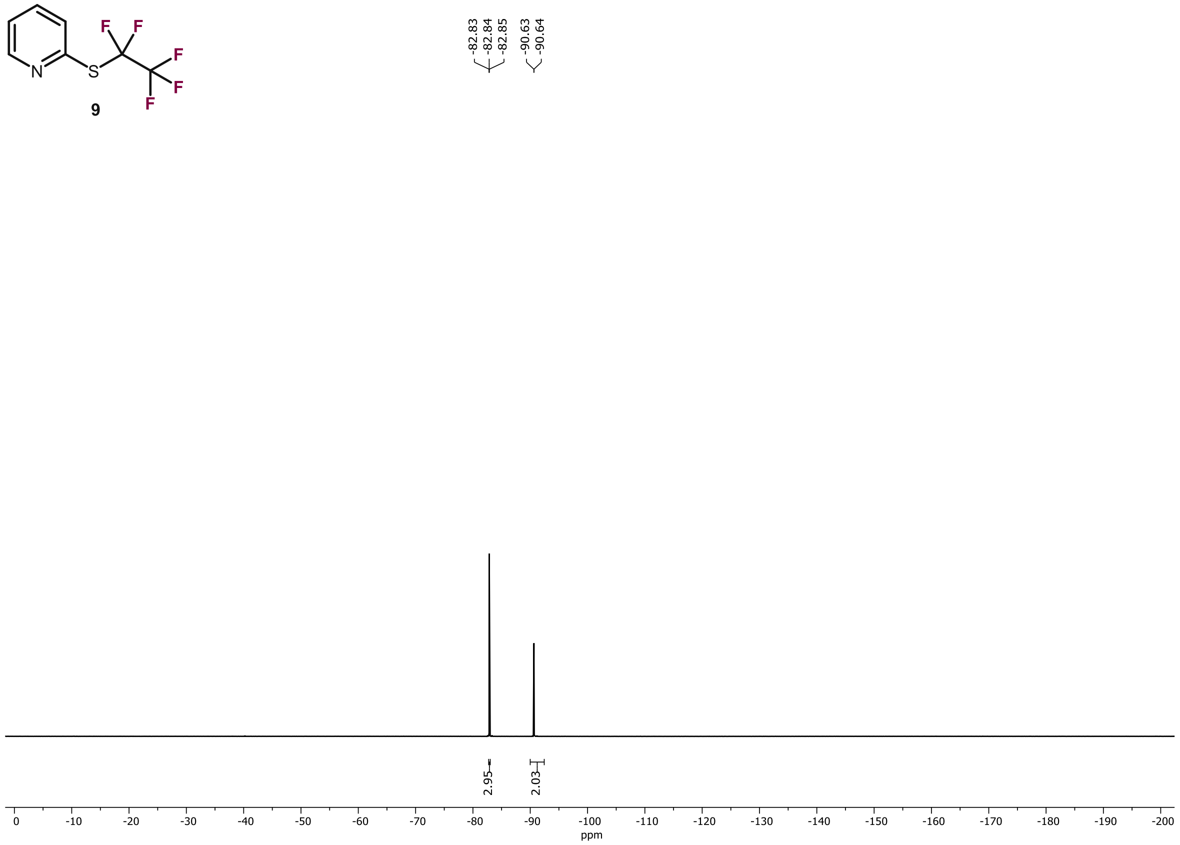

^{1}H NMR (400 MHz, $CDCl_3$) of **10**

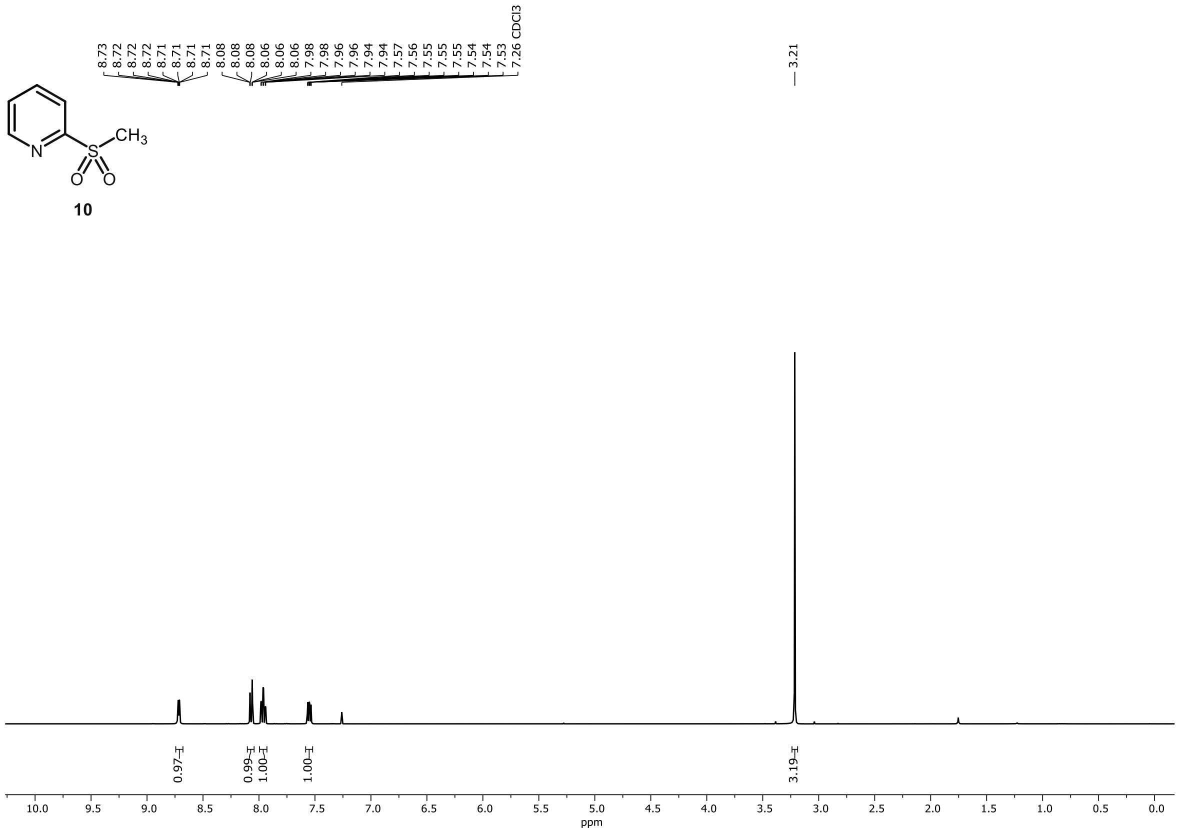

$^{13}C\{^{1}H\}$ NMR (100.6 MHz, $CDCl_3$) of **10**

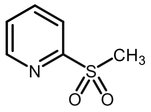

10

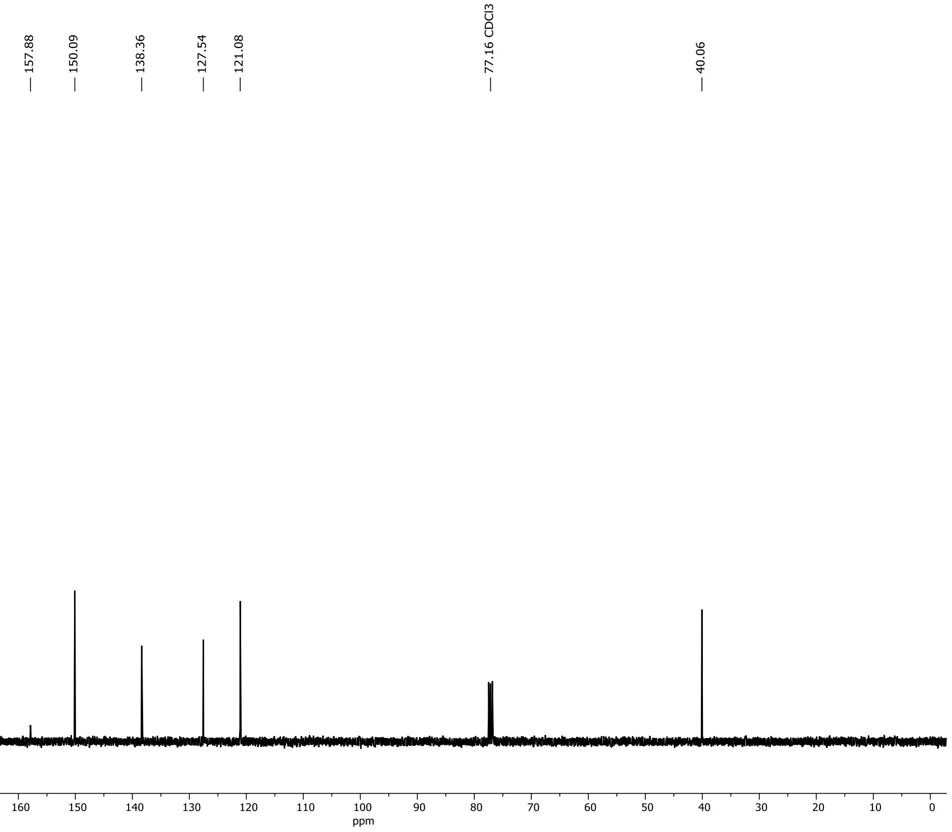

^{1}H NMR (400 MHz, $CDCl_3$) of **11**

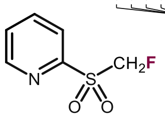

11

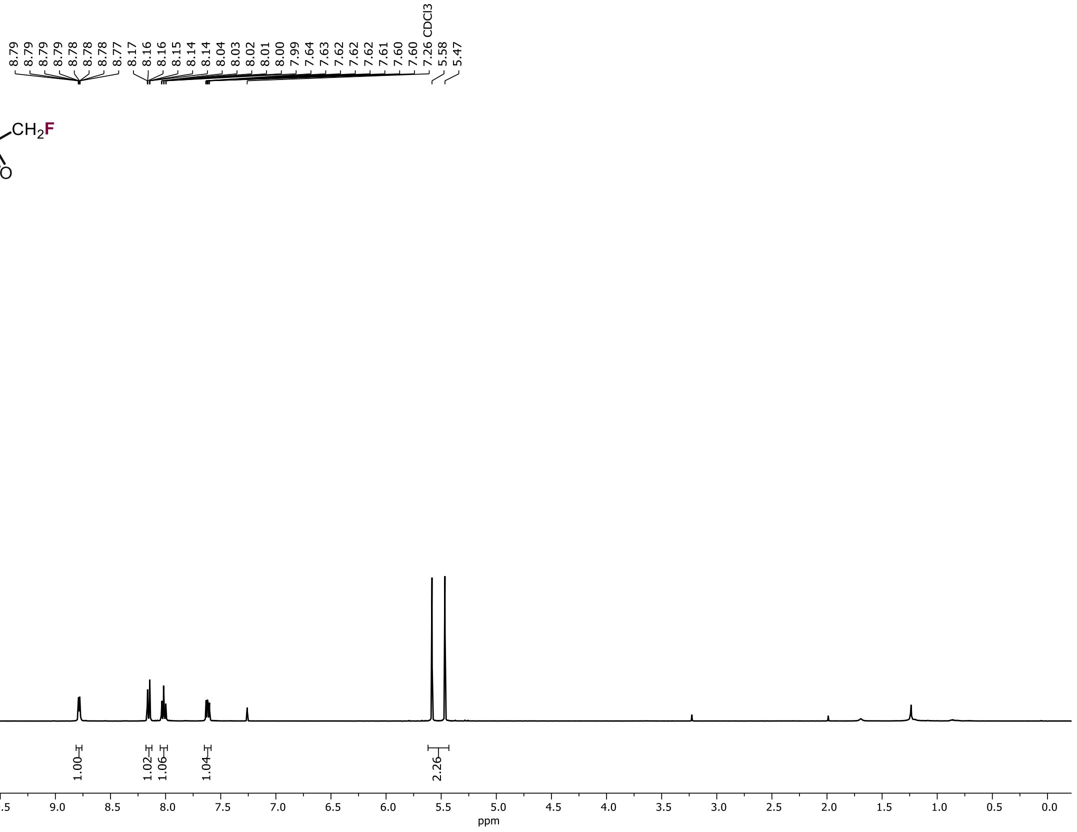

$^{13}C\{^{1}H\}$ NMR (100.6 MHz, $CDCl_3$) of **11**

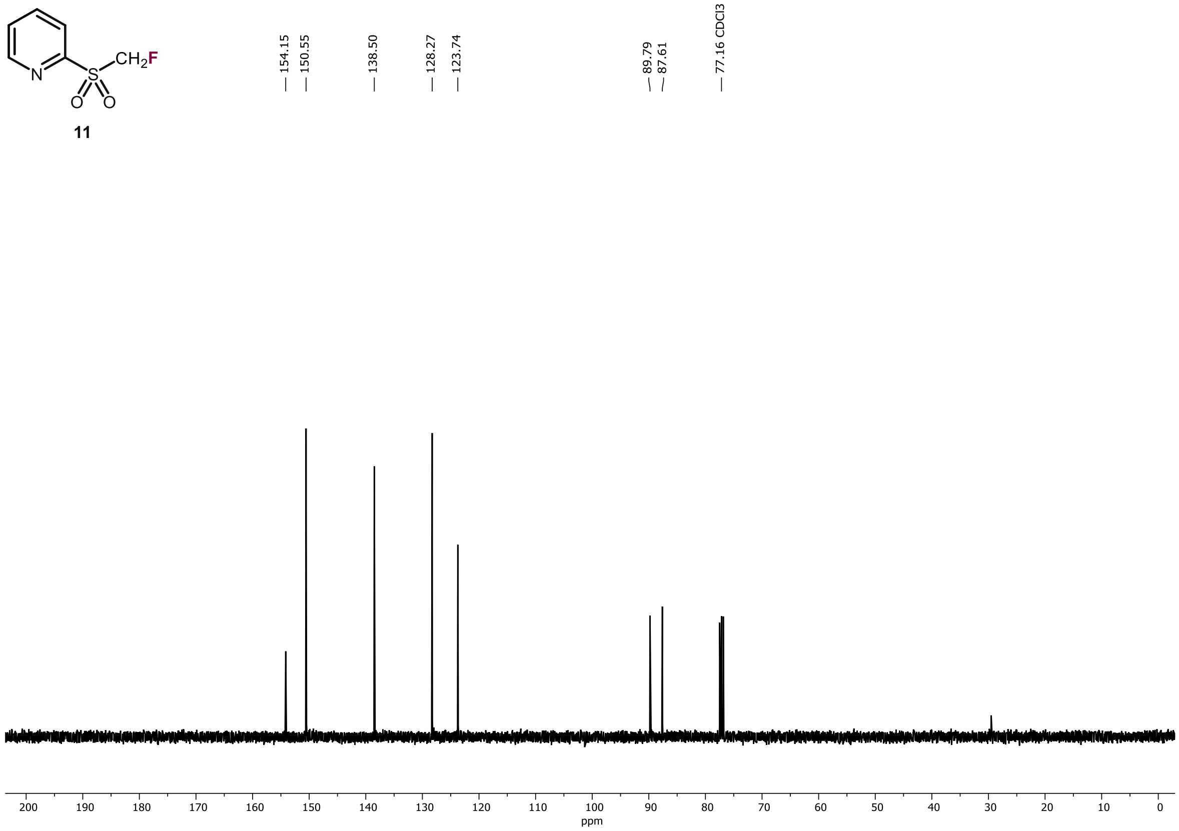

^{19}F NMR (376.5 MHz, $CDCl_3$) of **11**

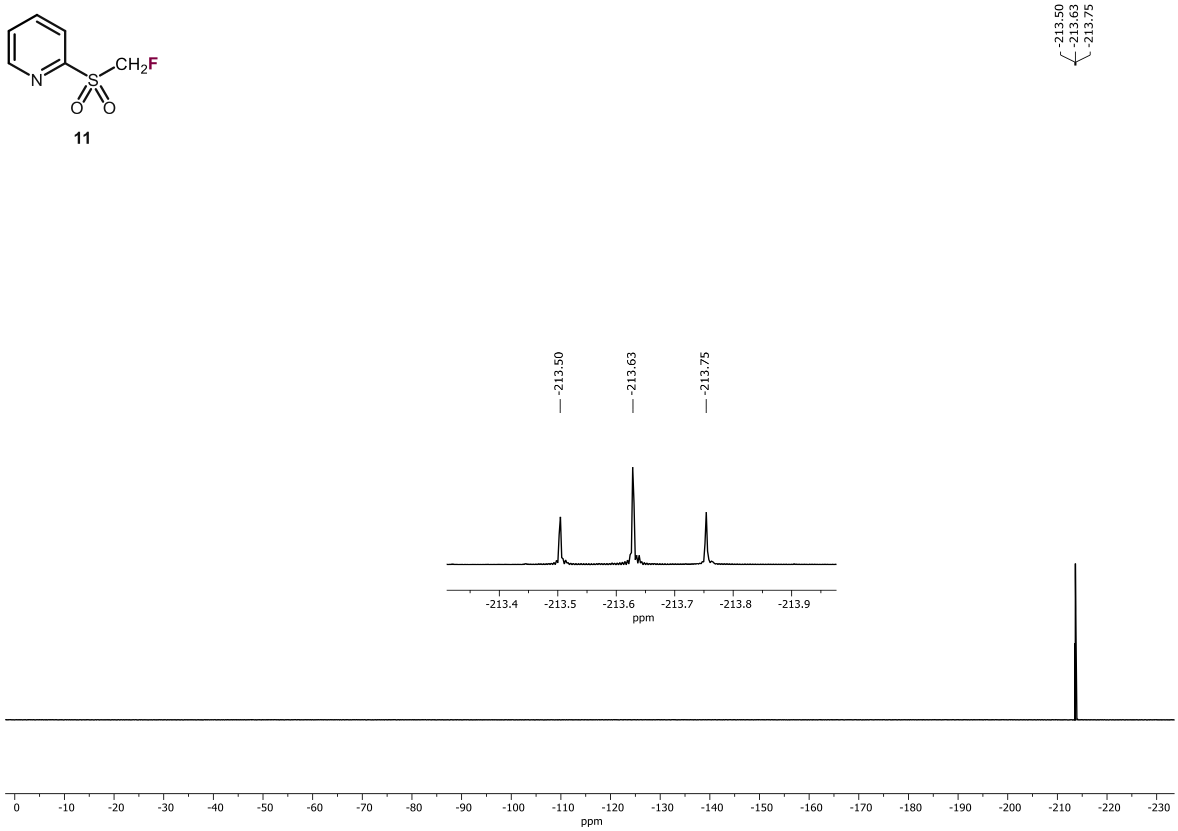

^{1}H NMR (400 MHz, $CDCl_3$) of **12**

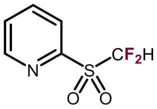

12

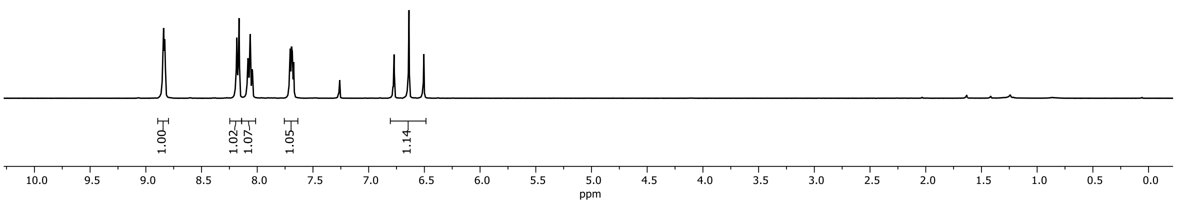

^{13}C{^{1}H} NMR (100.6 MHz, $CDCl_3$) of **12**

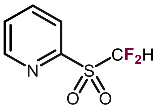

12

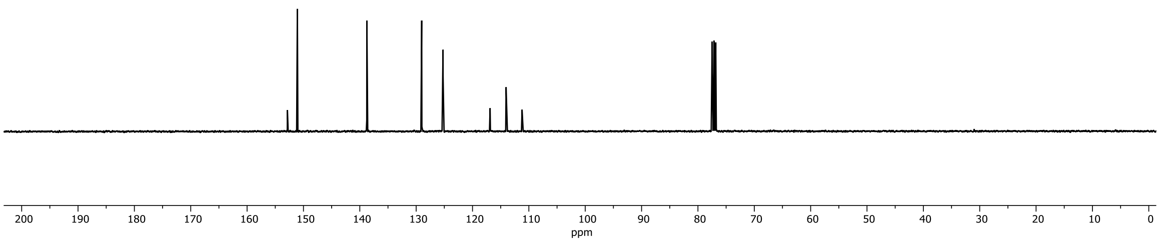

^{19}F NMR (376.5 MHz, $CDCl_3$) of **12**

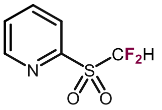

12

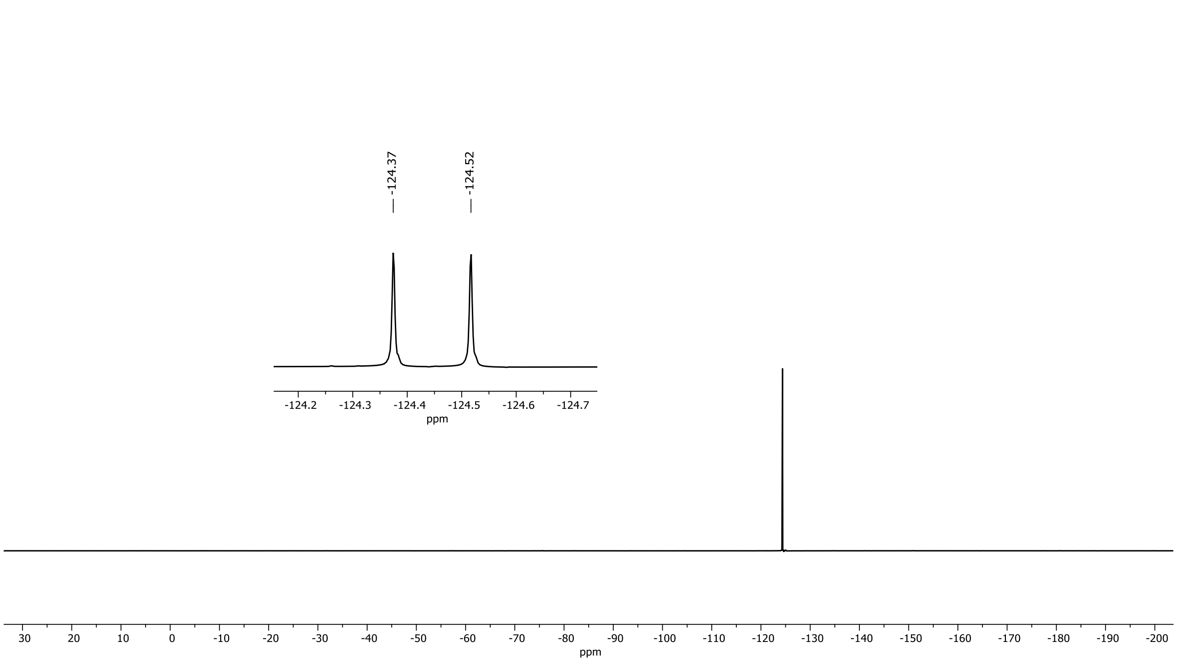

^{1}H NMR (400 MHz, $CDCl_3$) of **13**

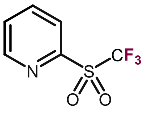

13

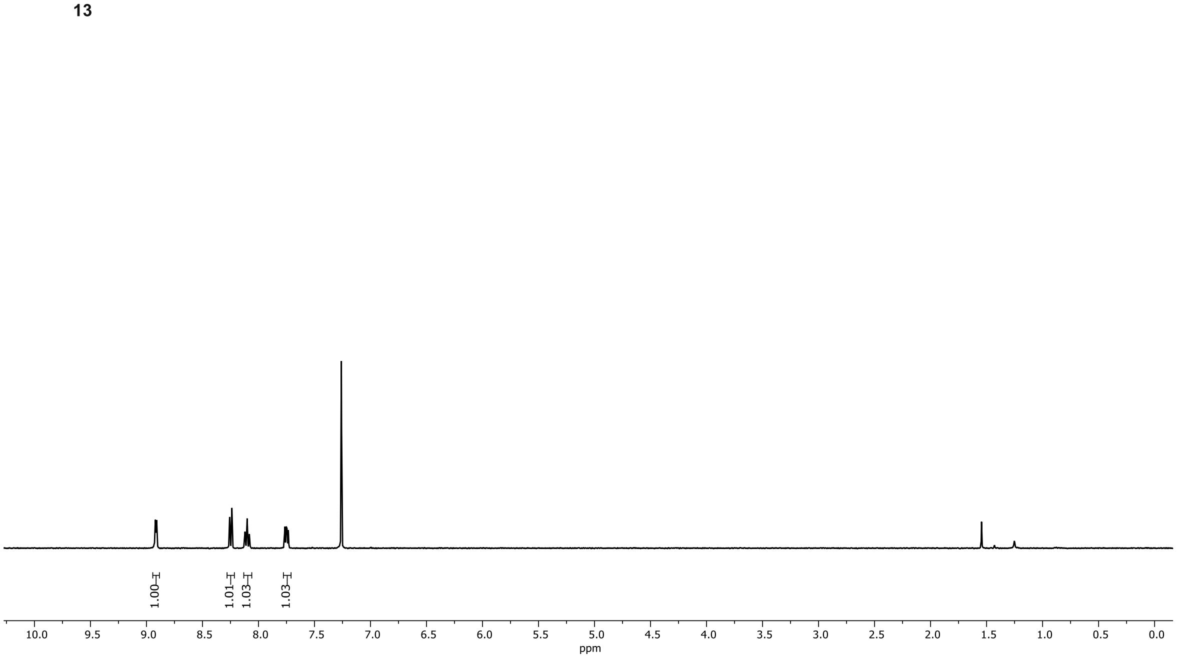

$^{13}C\{^{1}H\}$ NMR (100.6 MHz, $CDCl_3$) of **13**

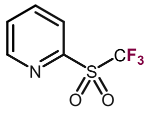

13

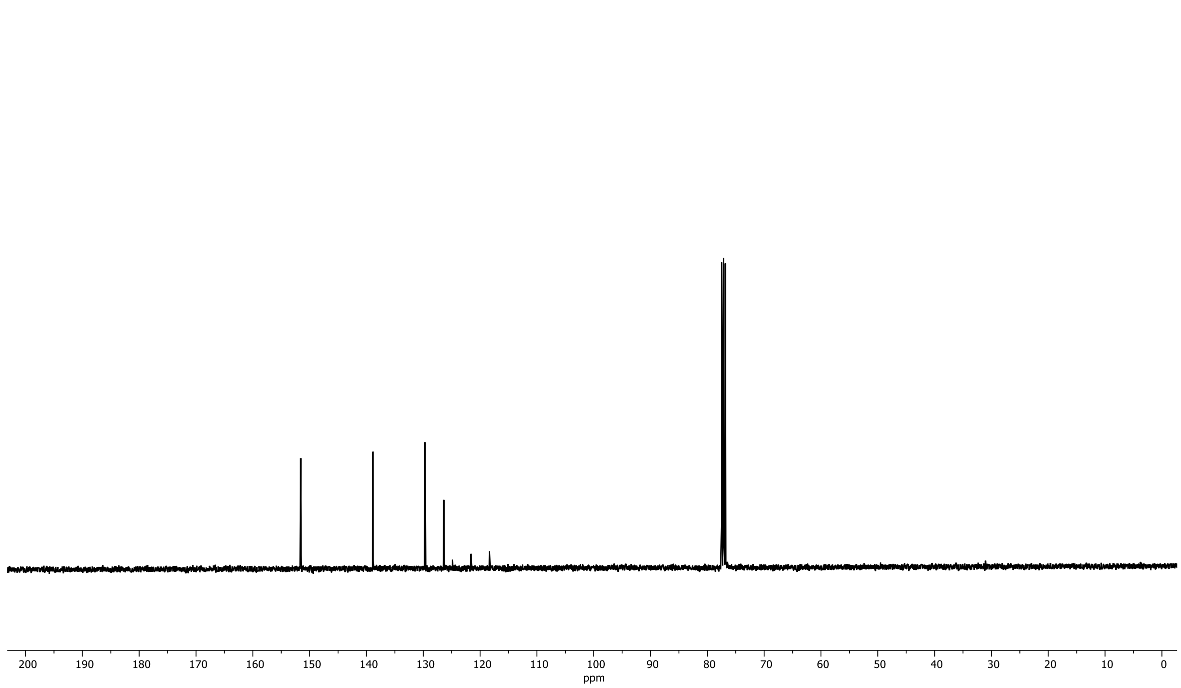

^{19}F NMR (376.5 MHz, $CDCl_3$) of **13**

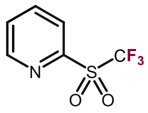

13

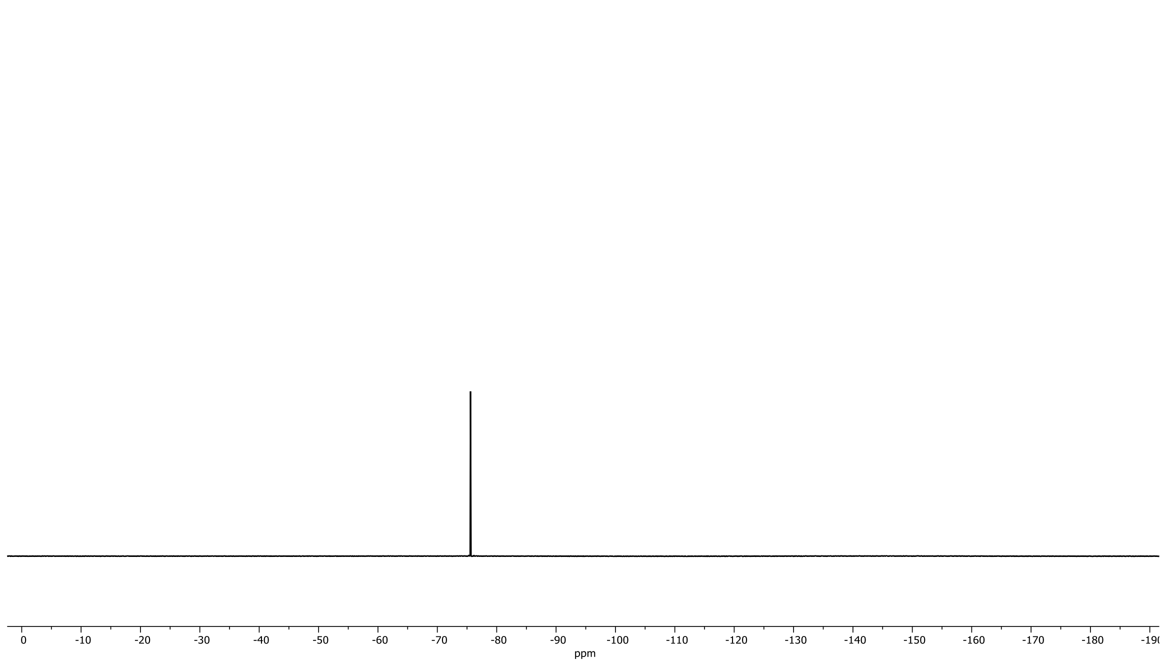

^{1}H NMR (400 MHz, $CDCl_3$) of **14**

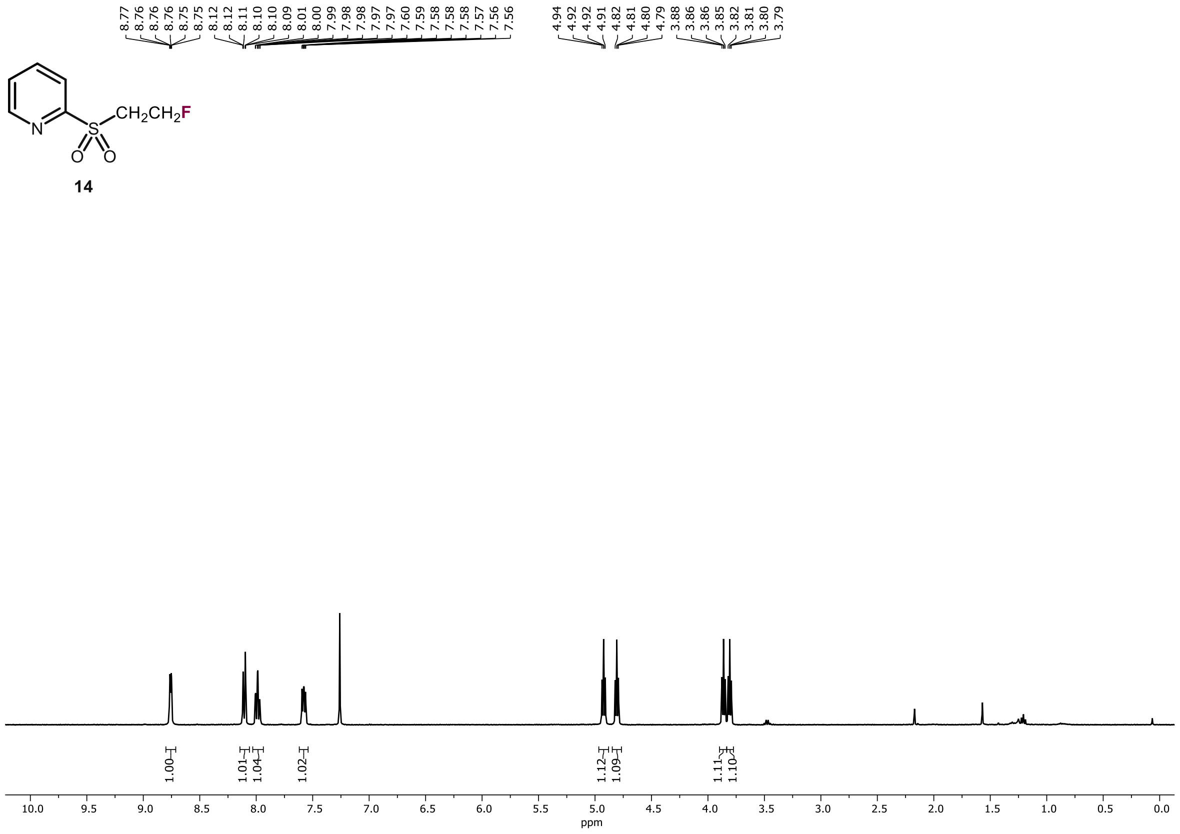

14

^{13}C{^{1}H} NMR (100.6 MHz, $CDCl_3$) of **14**

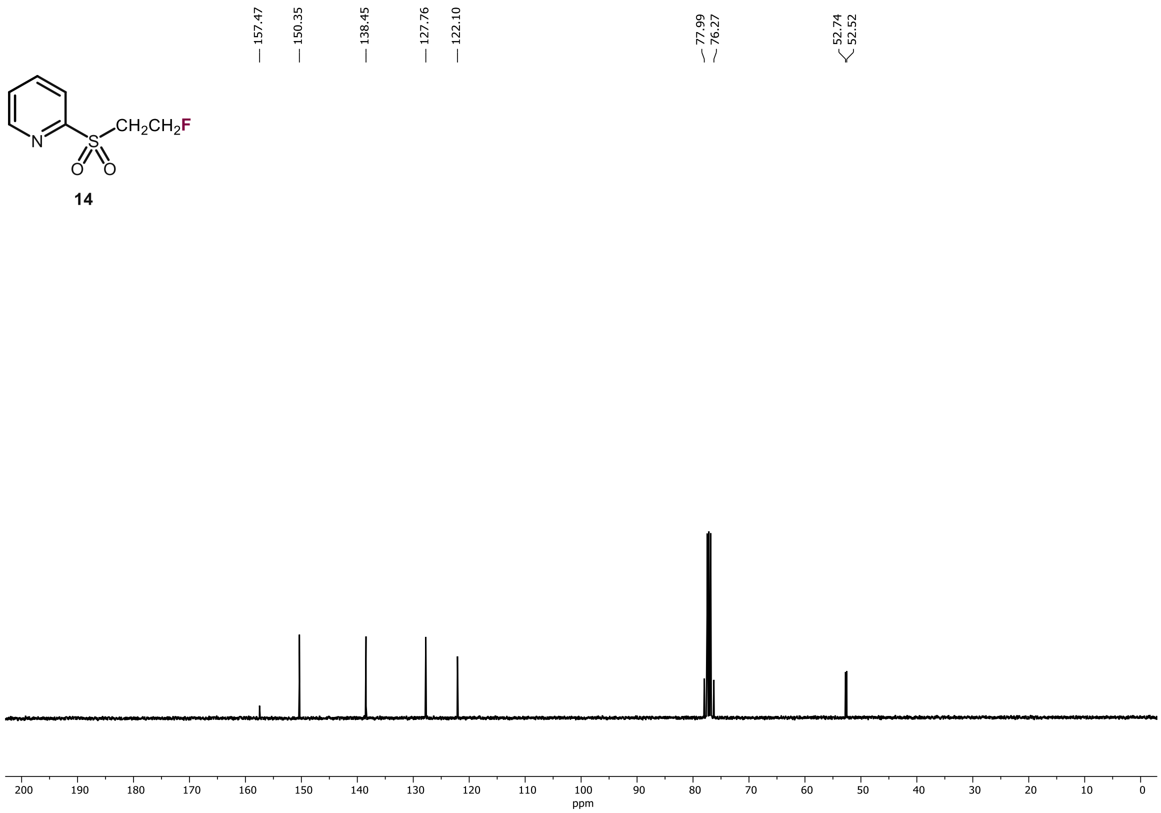

14

^{19}F NMR (376.5 MHz, $CDCl_3$) of **14**

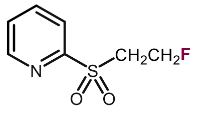

14

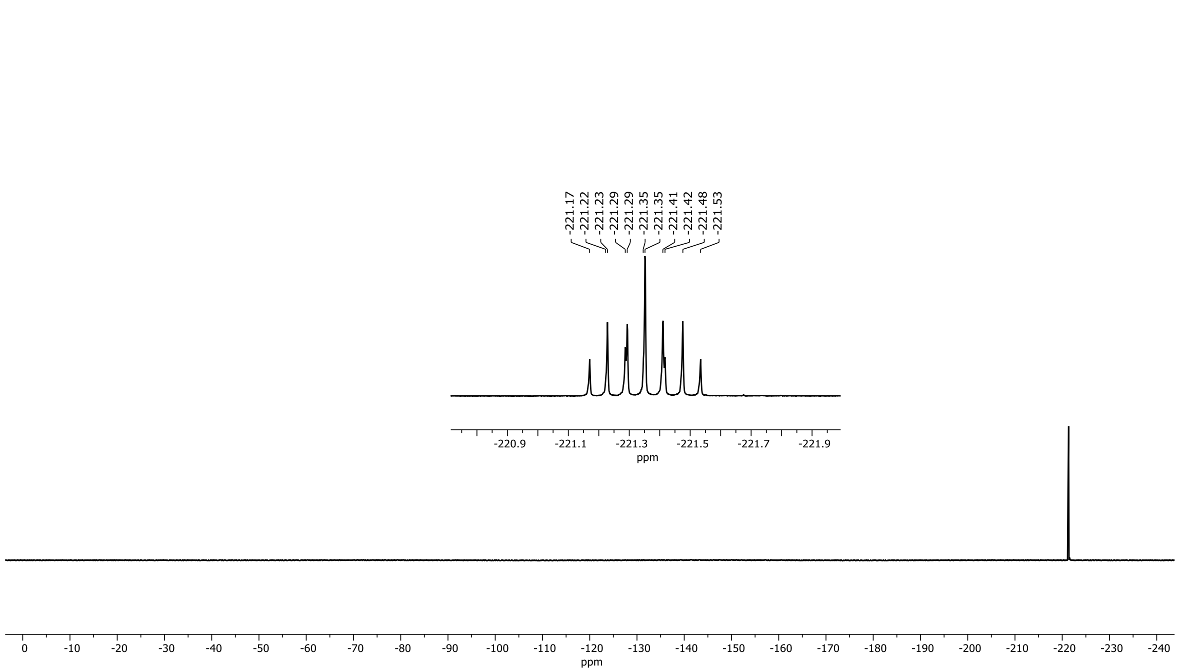

^{1}H NMR (400 MHz, $CDCl_3$) of **15**

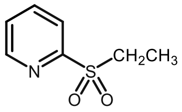

15

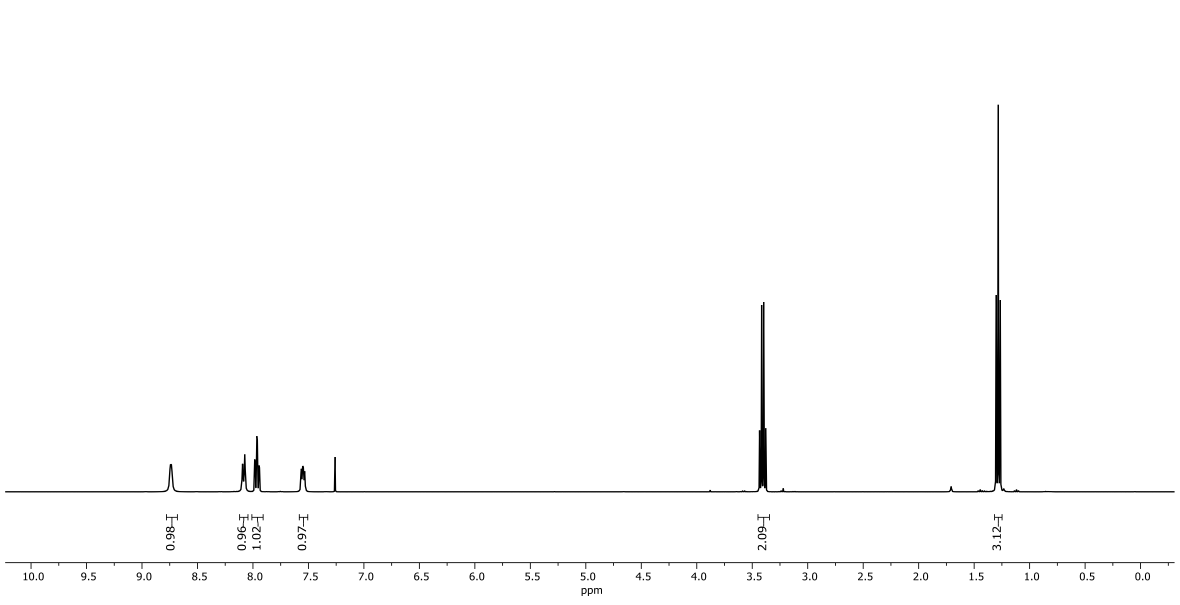

$^{13}C\{^{1}H\}$ NMR (100.6 MHz, $CDCl_3$) of **15**

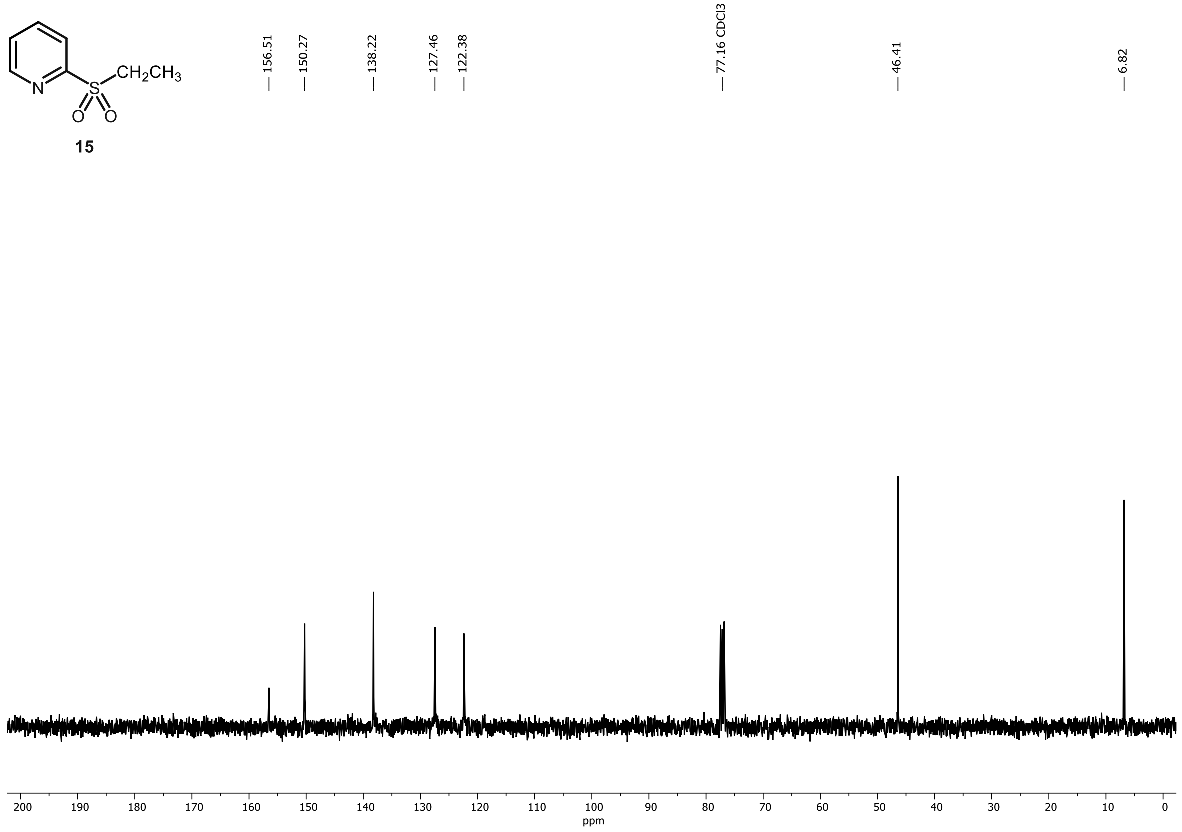

^{1}H NMR (400 MHz, $CDCl_3$) of **16**

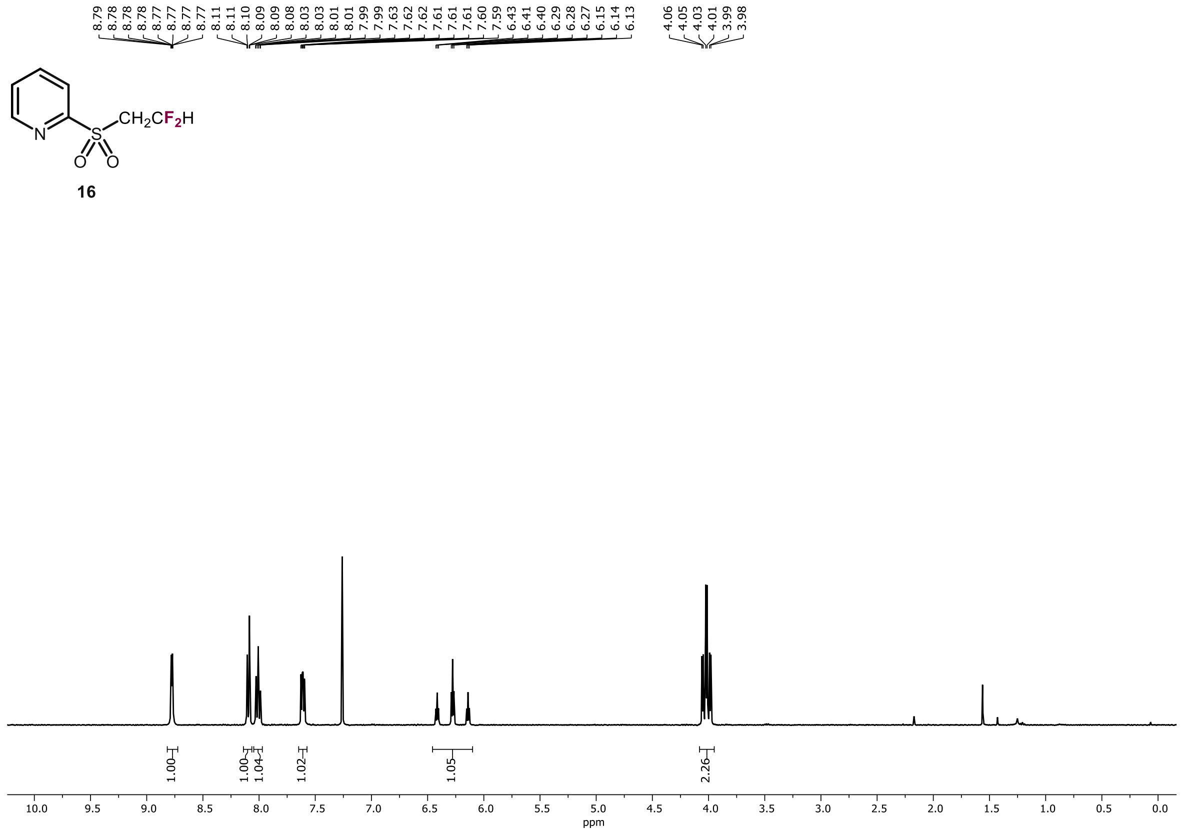

$^{13}C\{^{1}H\}$ NMR (100.6 MHz, $CDCl_3$) of **16**

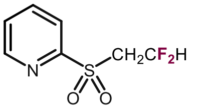

16

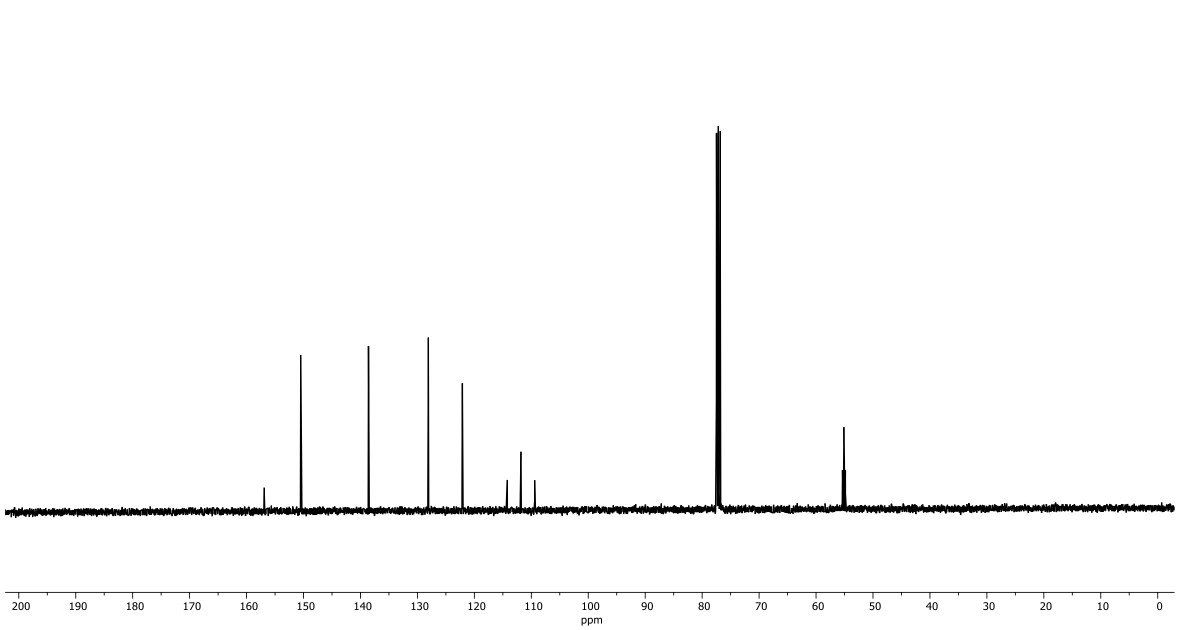

^{19}F NMR (376.5 MHz, $CDCl_3$) of **16**

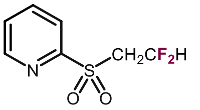

16

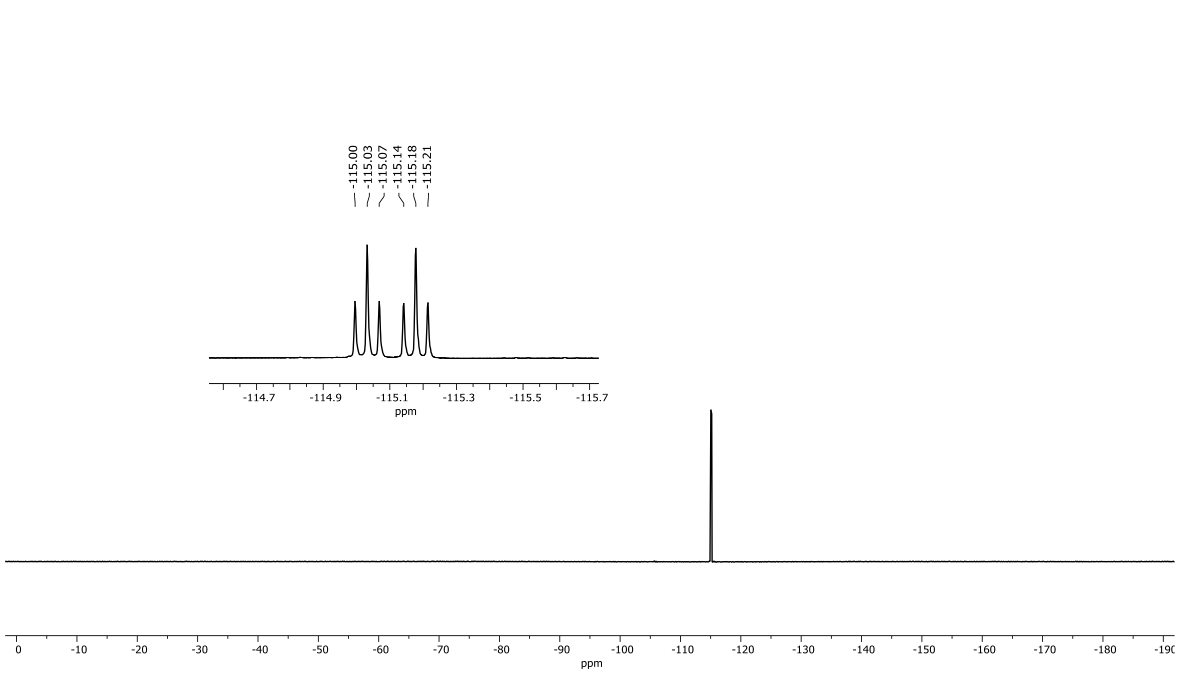

^{1}H NMR (400 MHz, $CDCl_3$) of **17**

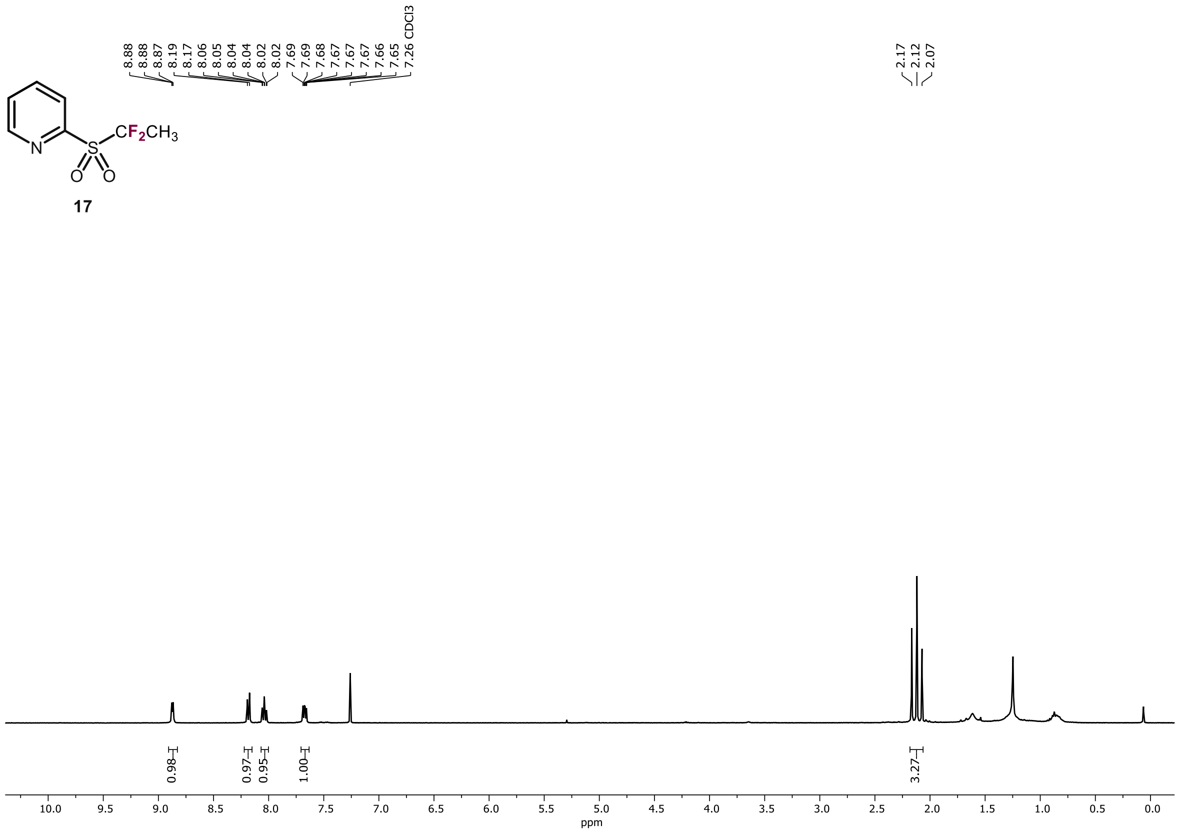

$^{13}C\{^{1}H\}$ NMR (100.6 MHz, $CDCl_3$) of **17**

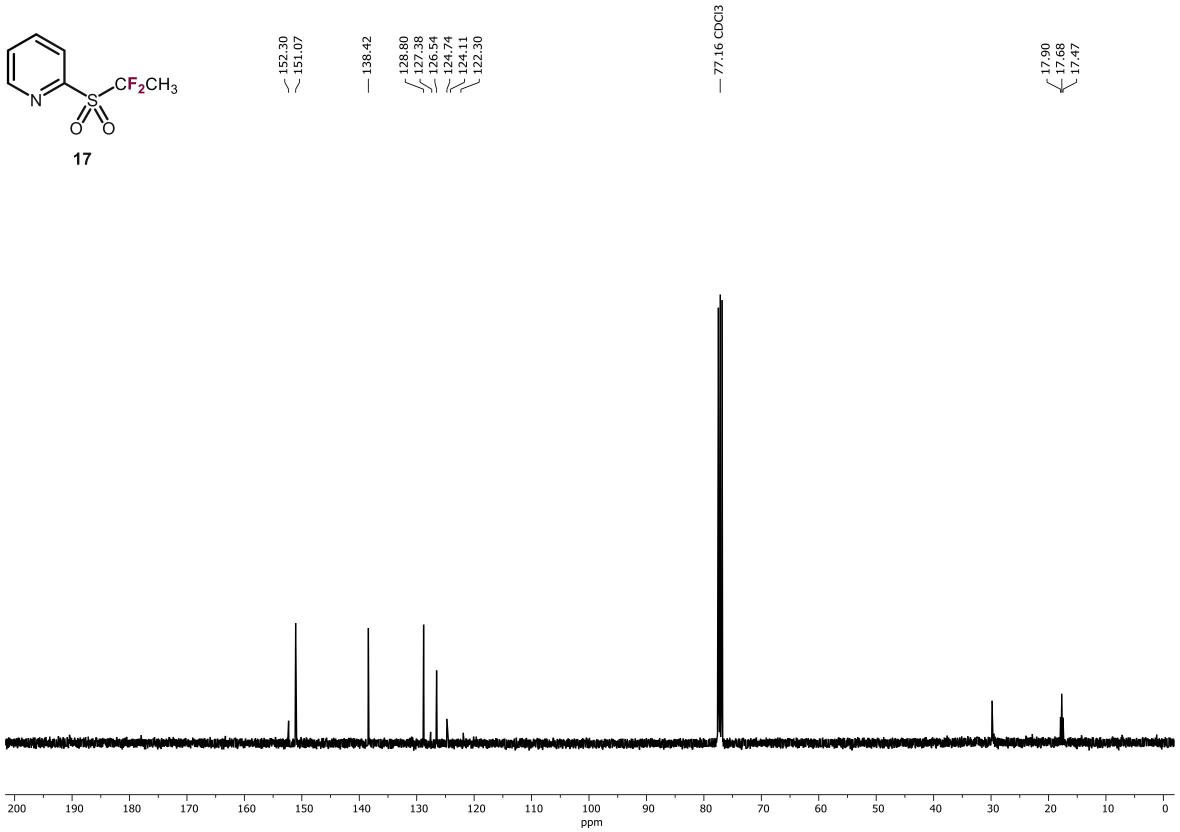

^{19}F NMR (376.5 MHz, CDCl$_3$) of **17**

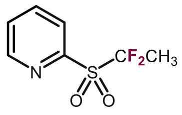

17

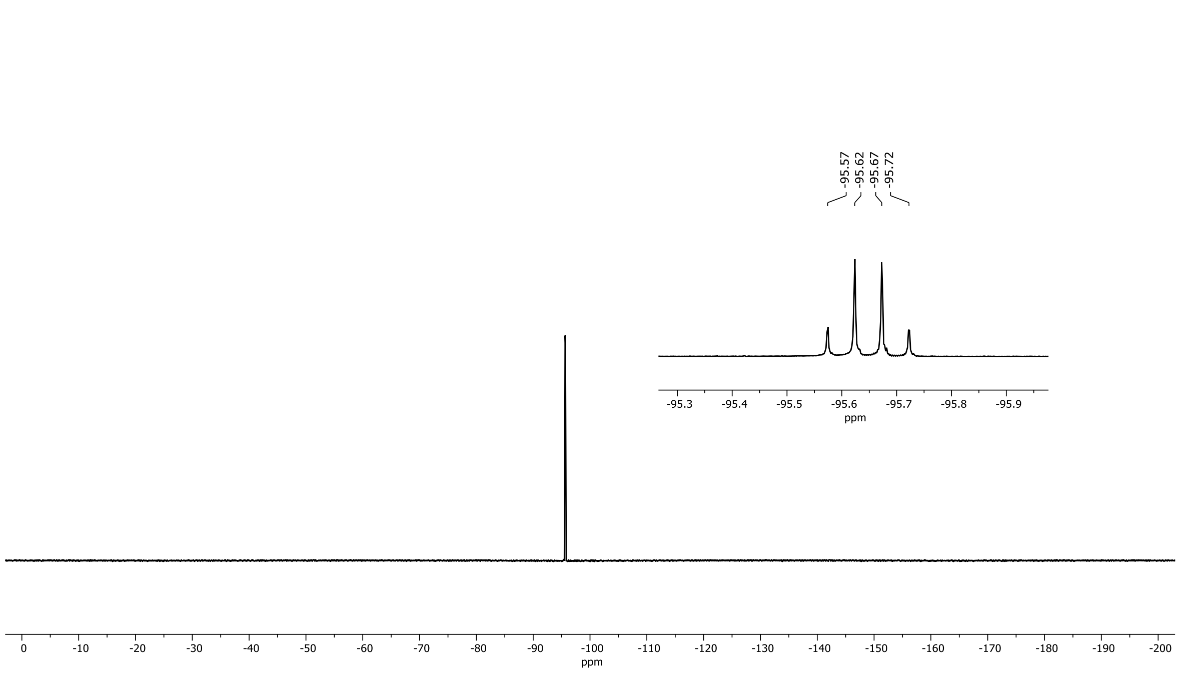

^{1}H NMR (400 MHz, CDCl$_3$) of **18**

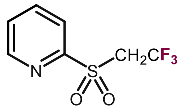

18

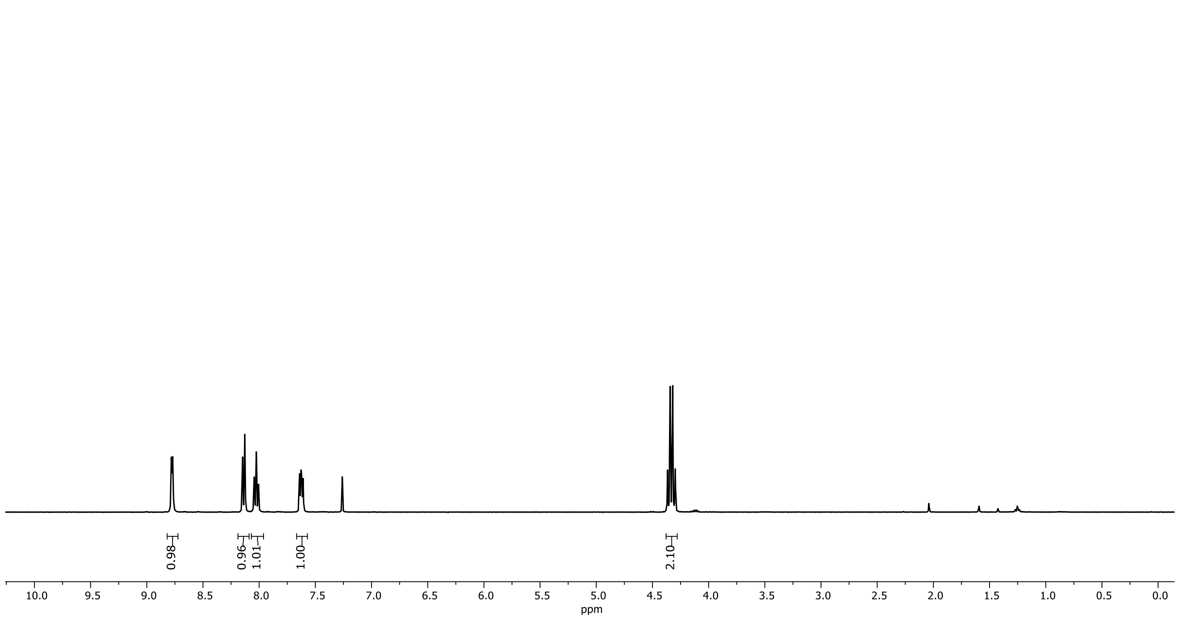

$^{13}C\{^{1}H\}$ NMR (100.6 MHz, $CDCl_3$) of **18**

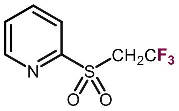

18

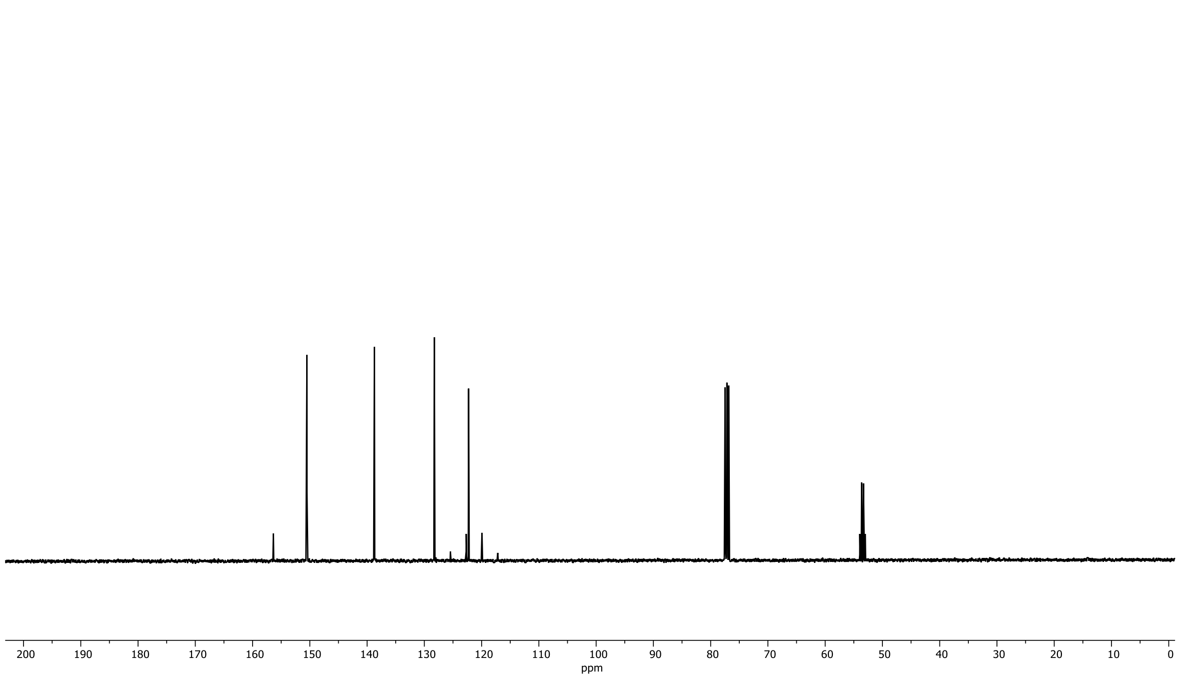

^{19}F NMR (376.5 MHz, $CDCl_3$) of **18**

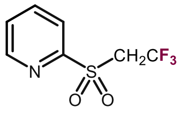

18

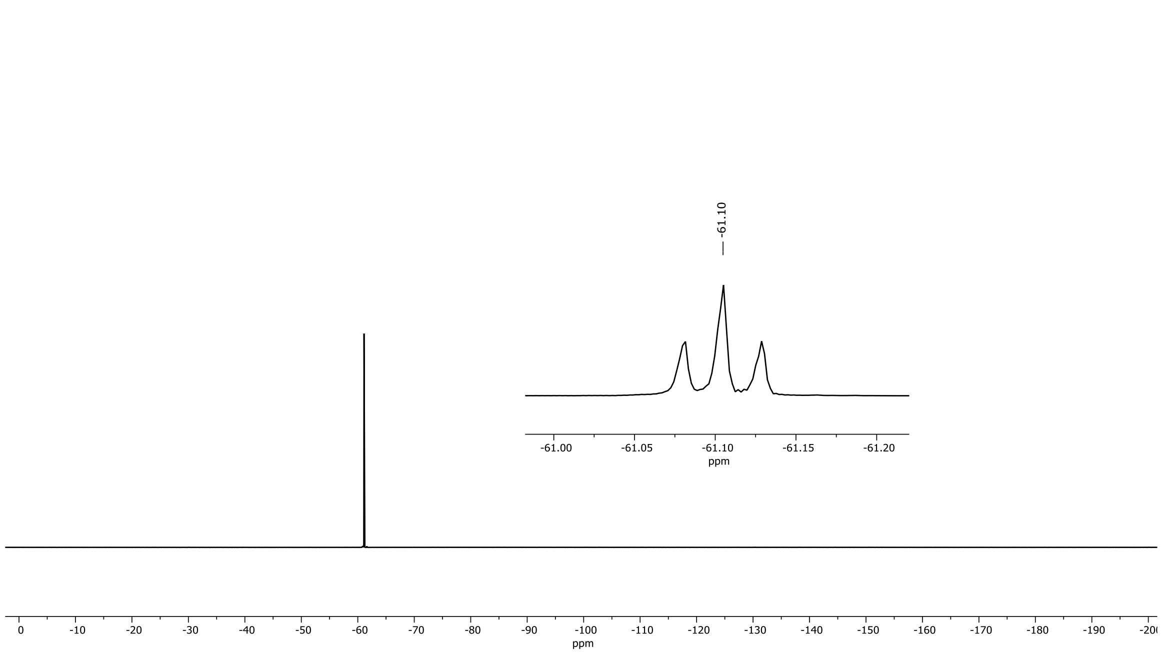

^{1}H NMR (400 MHz, $CDCl_3$) of **19**

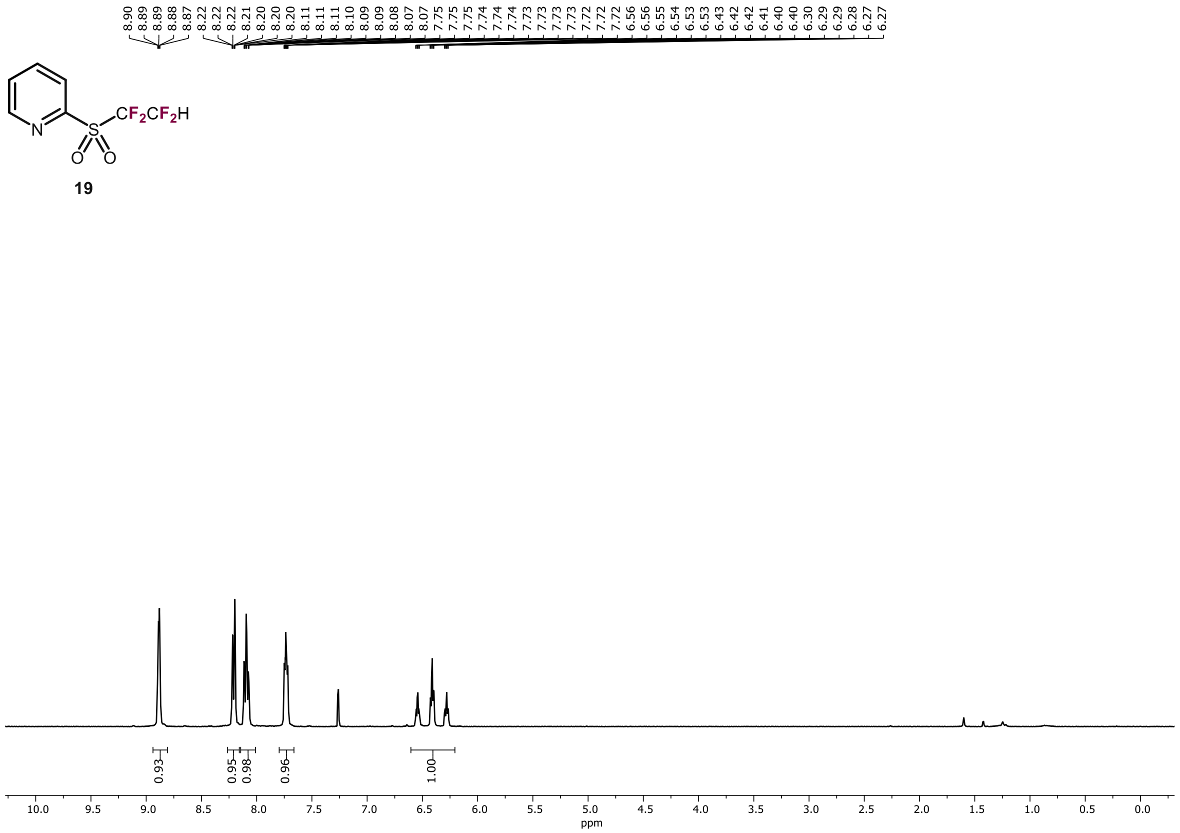

^{13}C{^{1}H} NMR (100.6 MHz, $CDCl_3$) of **19**

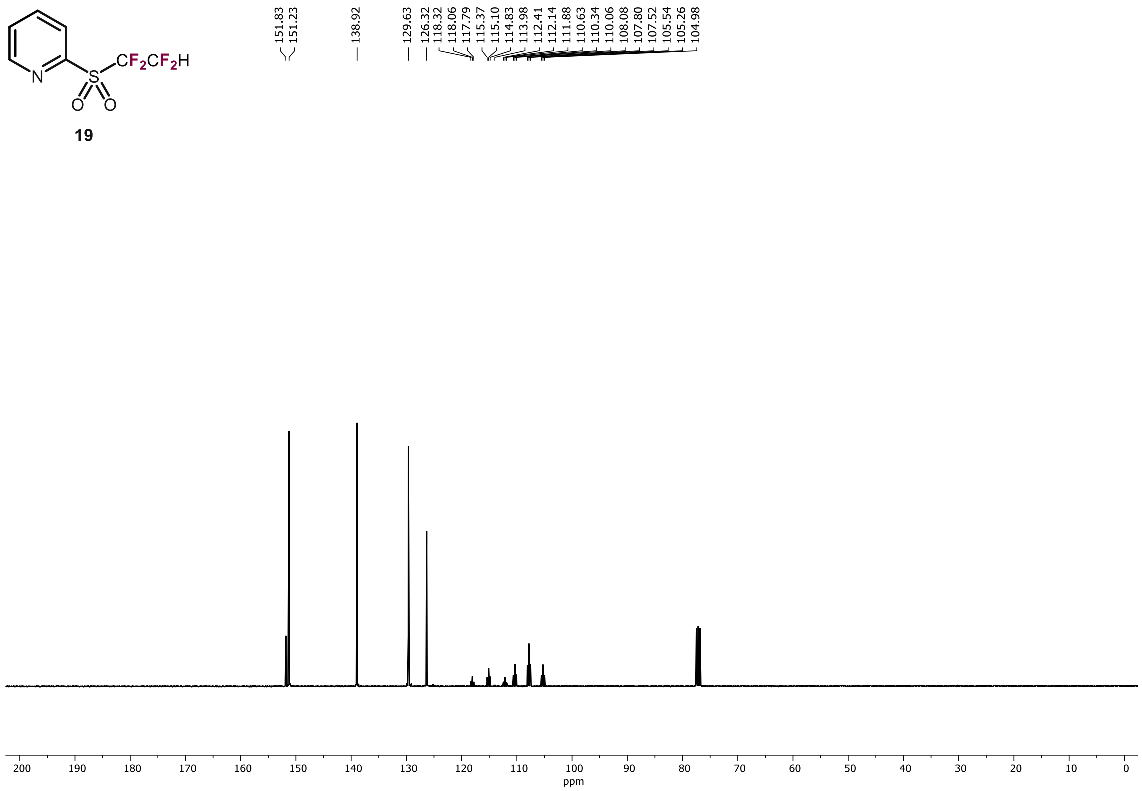

^{19}F NMR (376.5 MHz, $CDCl_3$) of **19**

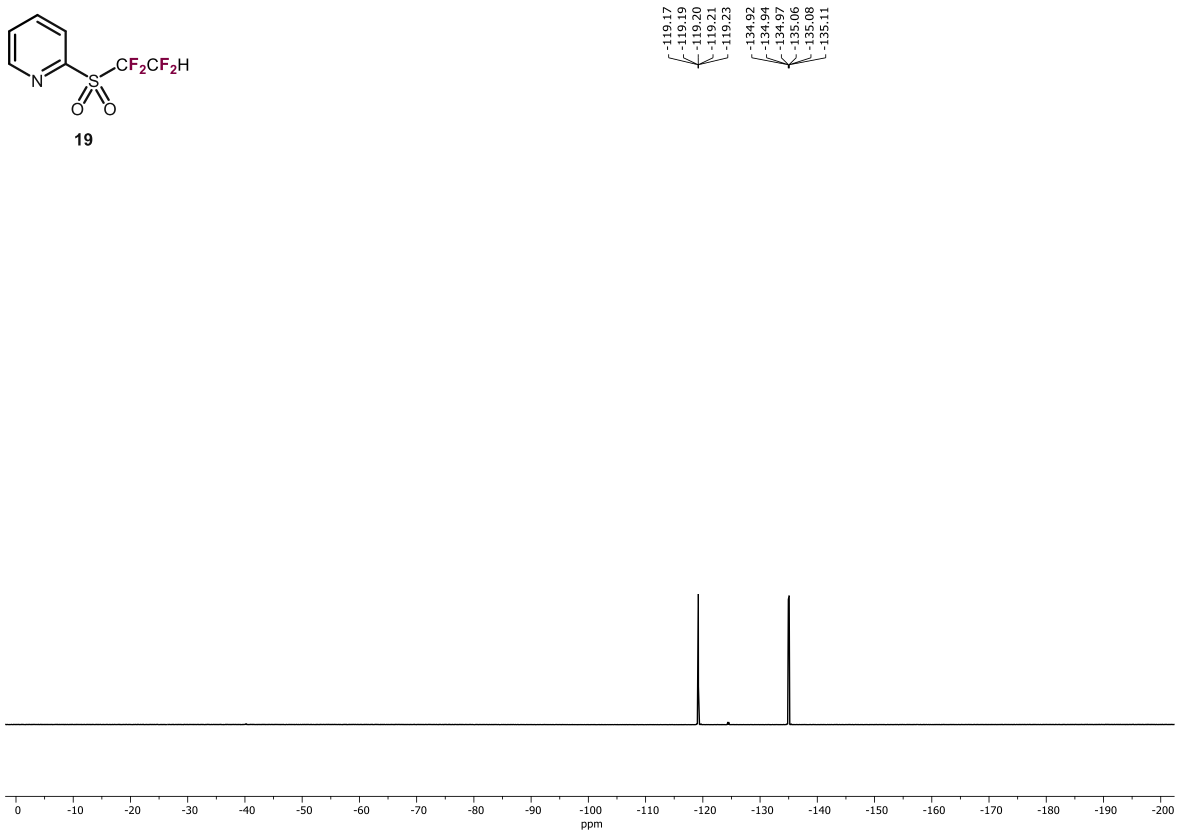

19

^{1}H NMR (400 MHz, $CDCl_3$) of **20**

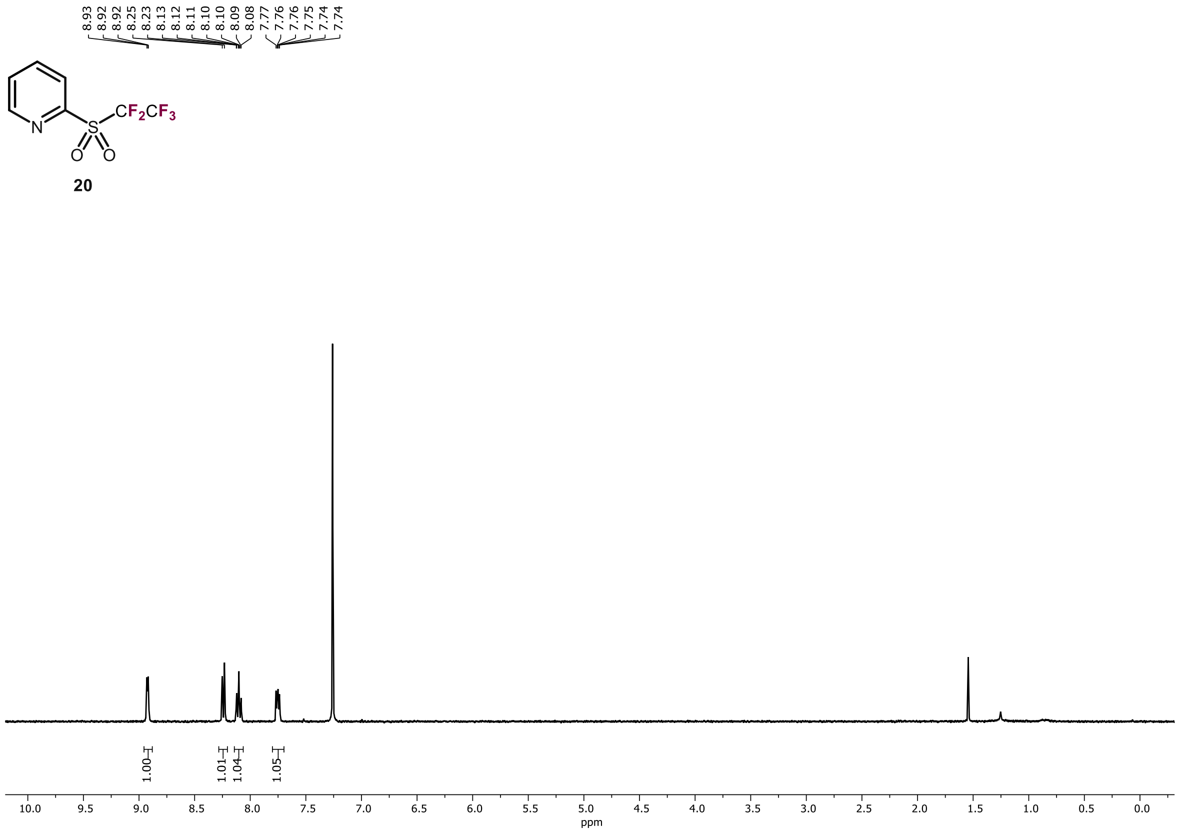

20

$^{13}C\{^{1}H\}$ NMR (100.6 MHz, $CDCl_3$) of **20**

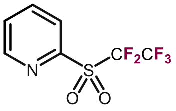

20

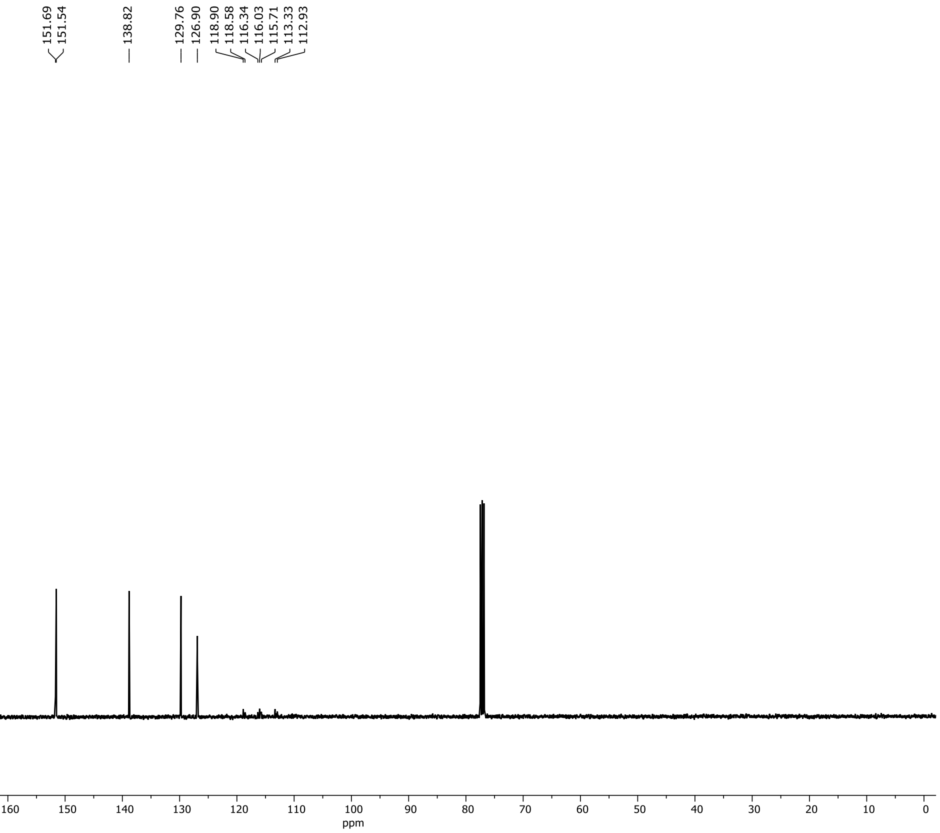

^{19}F NMR (376.5 MHz, $CDCl_3$) of **20**

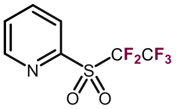

20

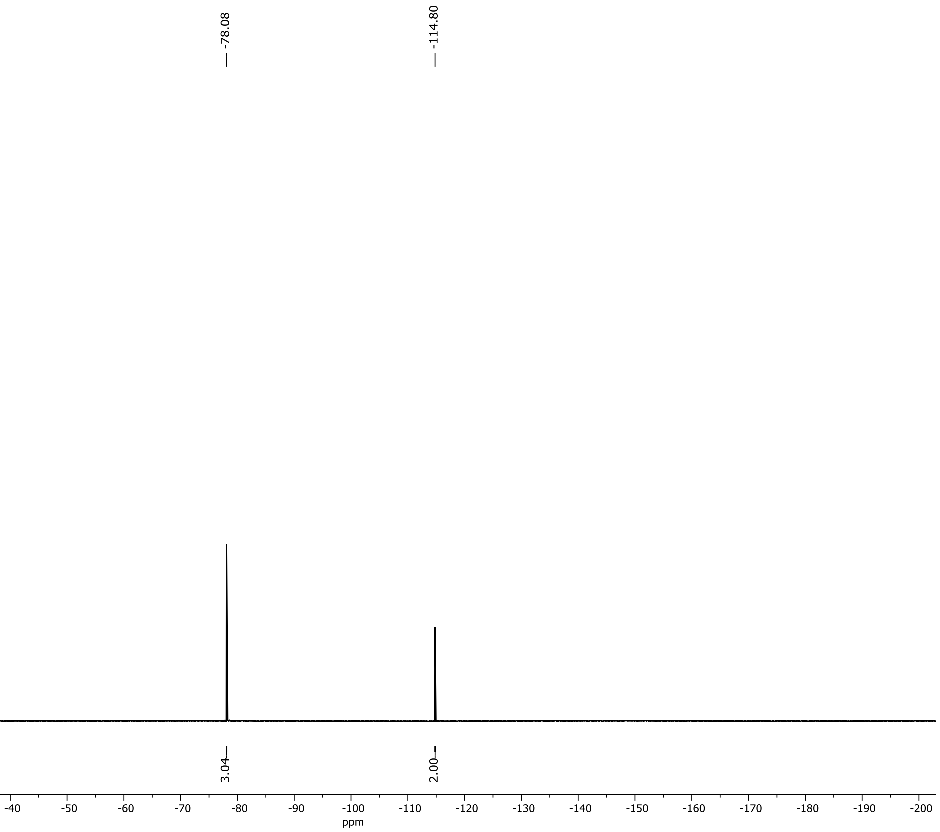

^{1}H NMR (400 MHz, $CDCl_3$) of **21**

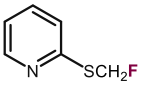

21

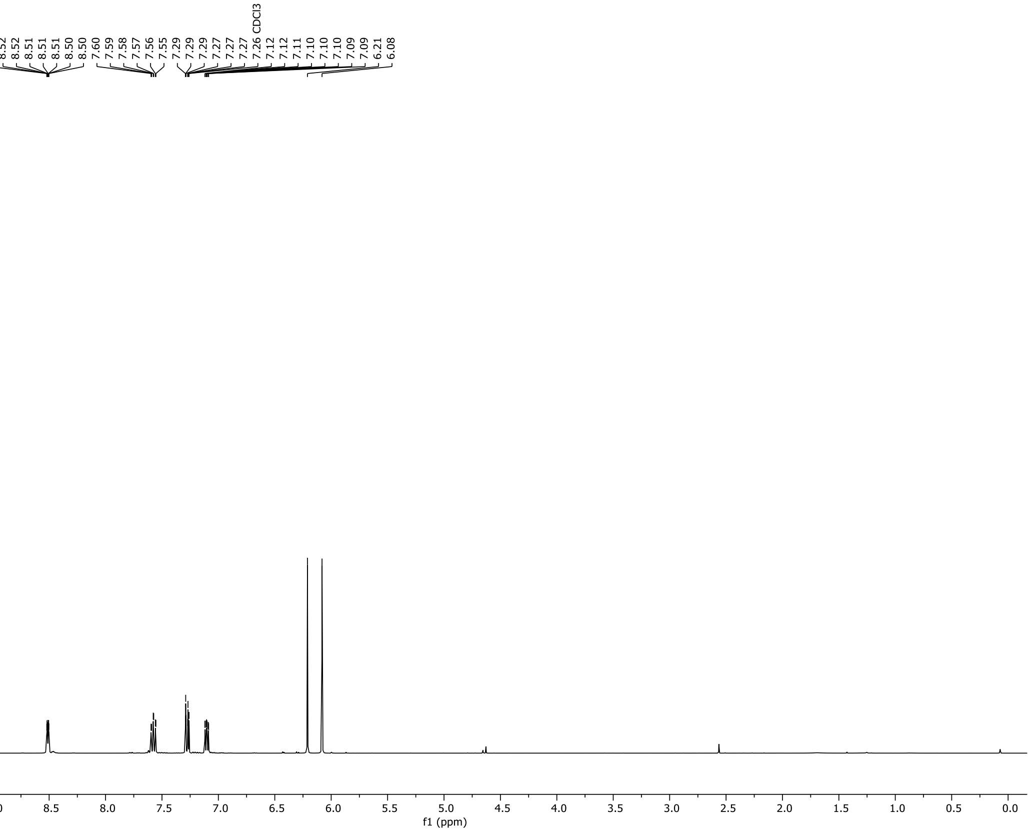

$^{13}C\{^{1}H\}$ NMR (100.6 MHz, $CDCl_3$) of **21**

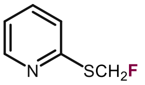

21

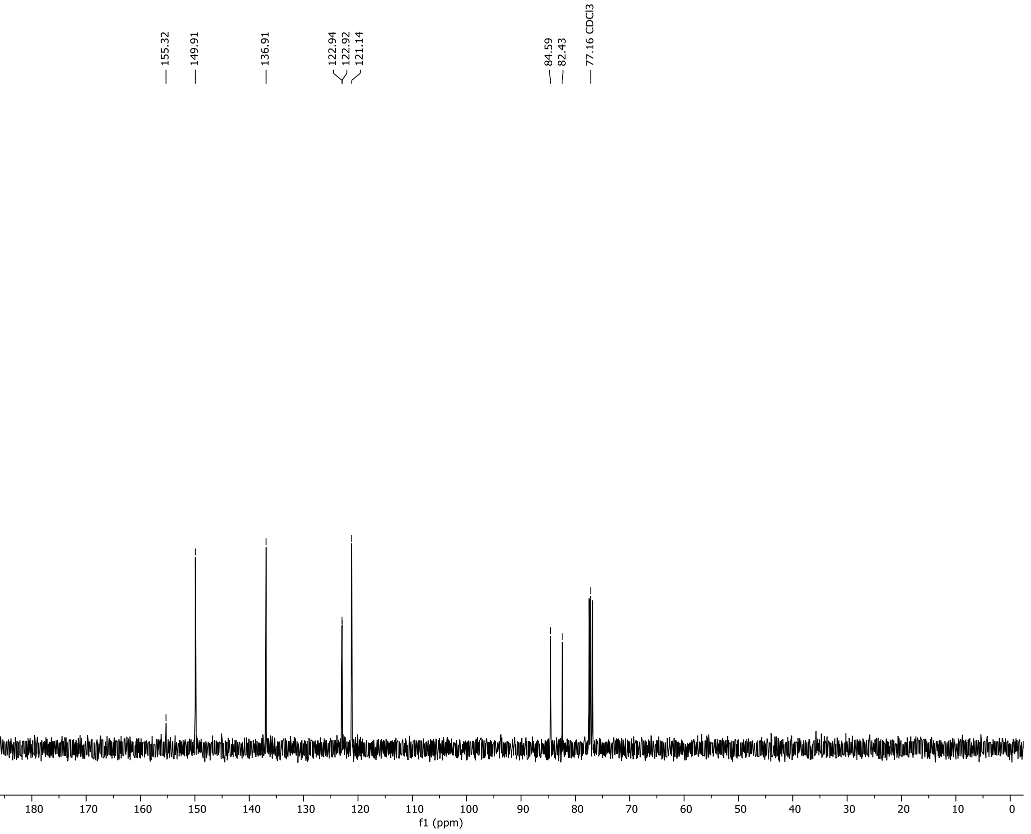

^{19}F NMR (376.5 MHz, CDCl$_3$) of **21**

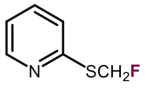

21

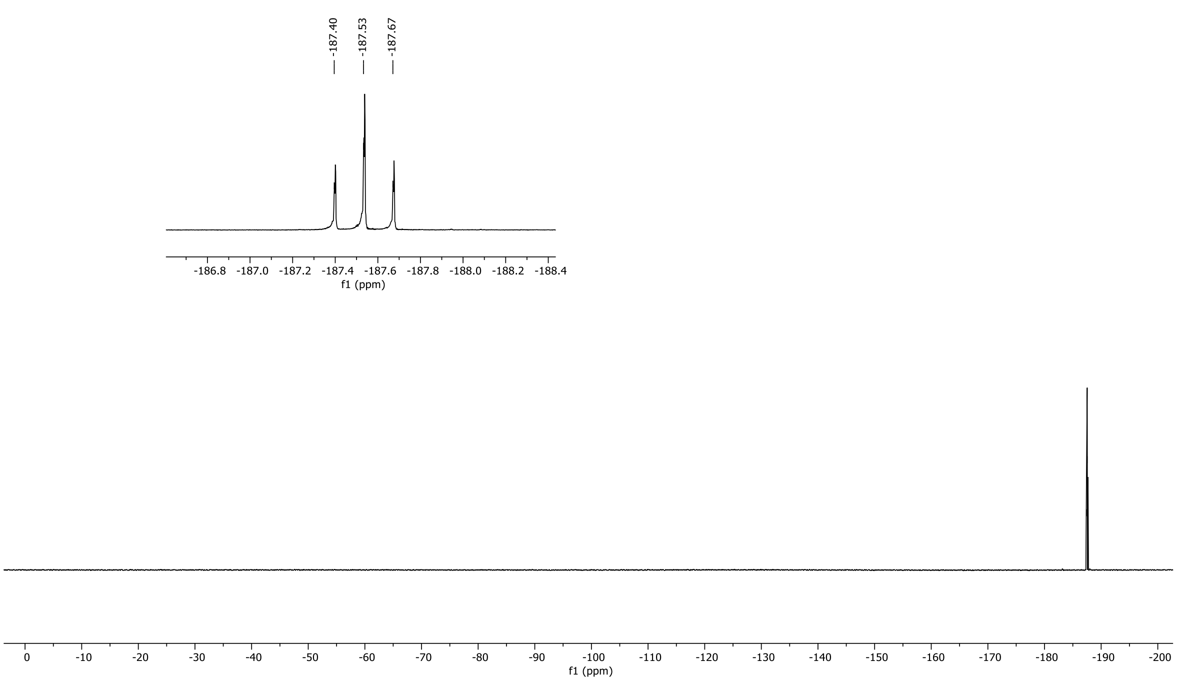

^{1}H NMR (400 MHz, CDCl$_3$) of **22**

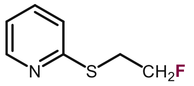

22

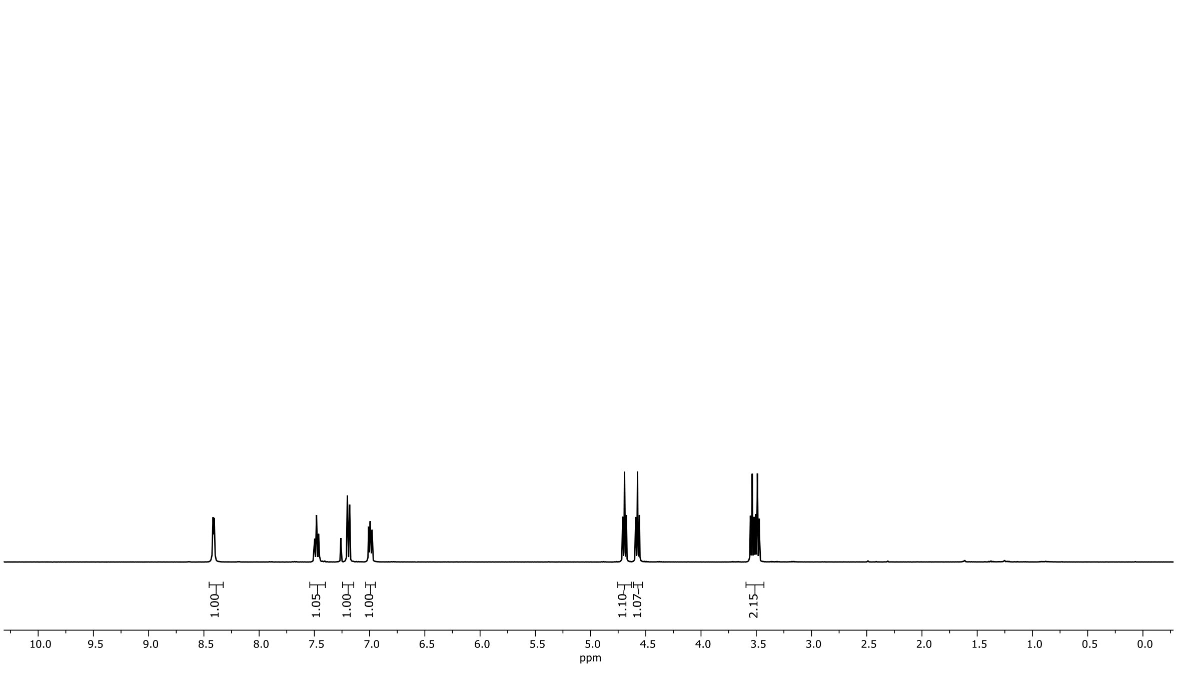

$^{13}C\{^{1}H\}$ NMR (100.6 MHz, $CDCl_3$) of **22**

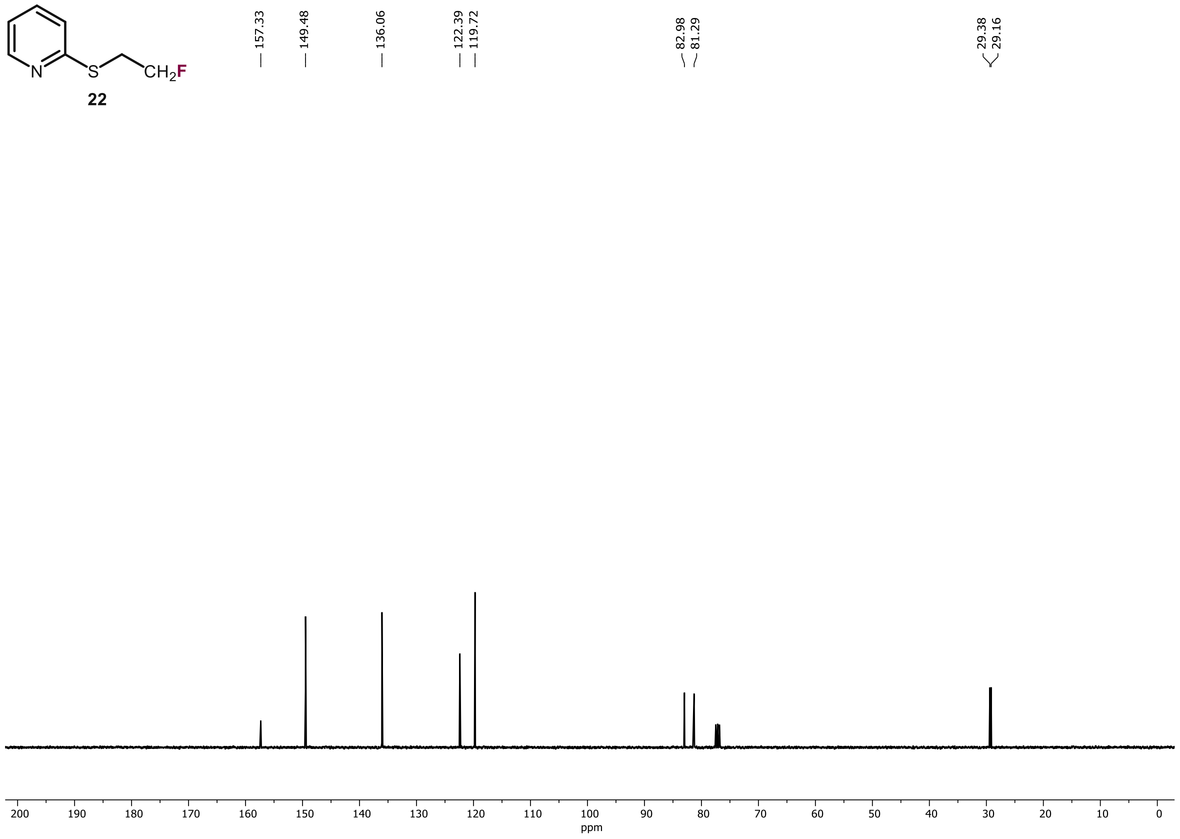

^{19}F NMR (376.5 MHz, $CDCl_3$) of **22**

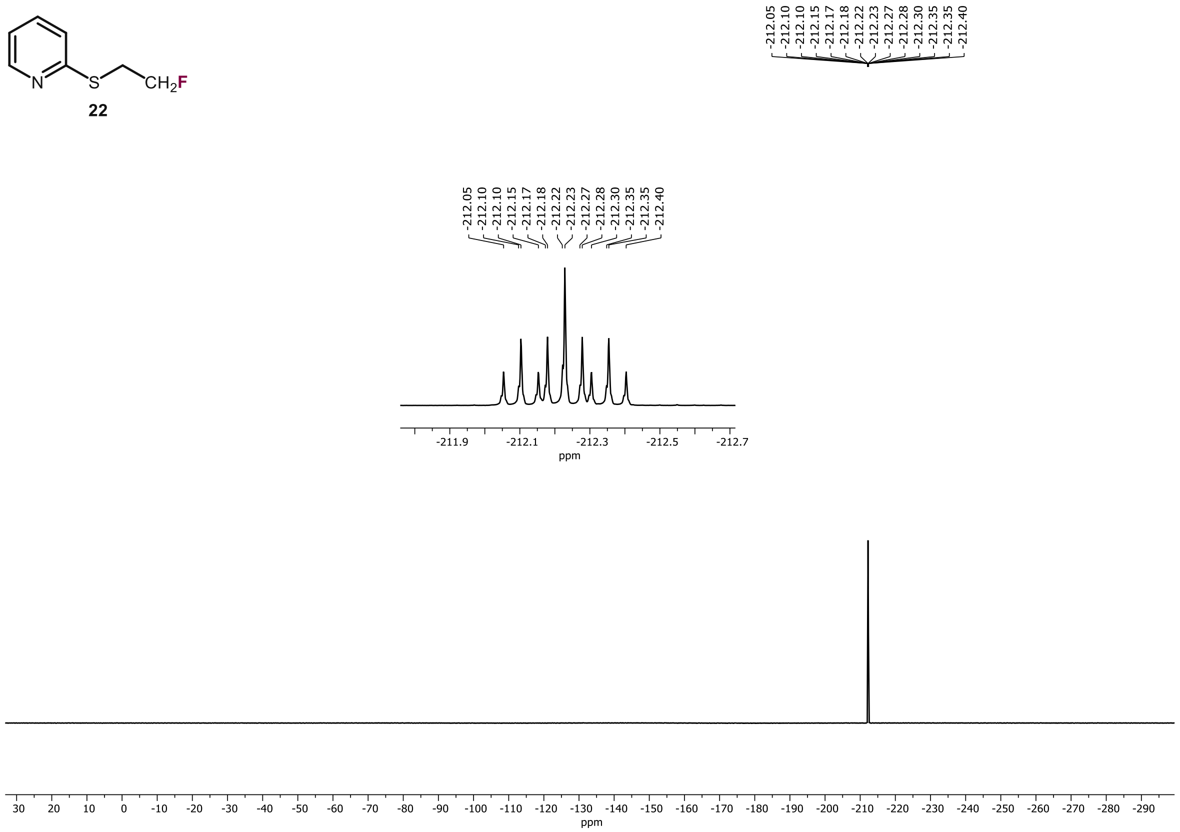

^{1}H NMR (400 MHz, $CDCl_3$) of **S1**

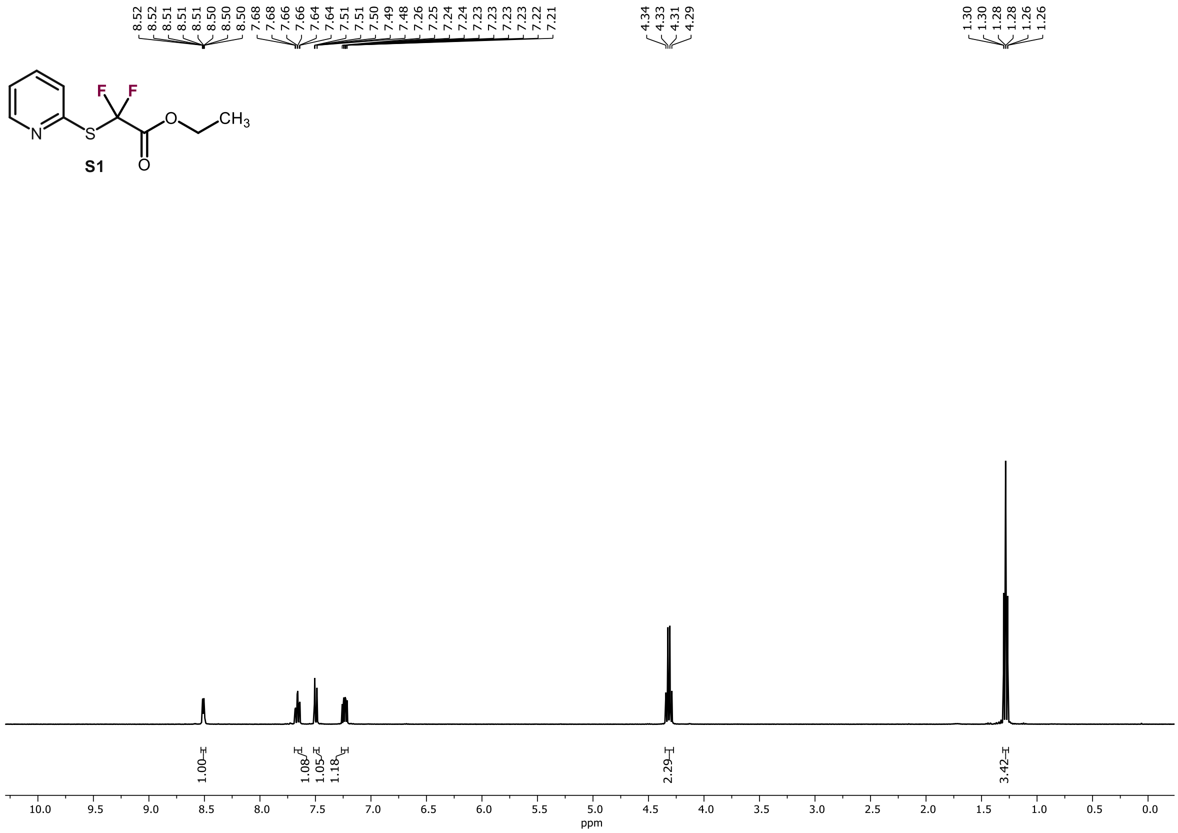

$^{13}C\{^{1}H\}$ NMR (100.6 MHz, $CDCl_3$) of **S1**

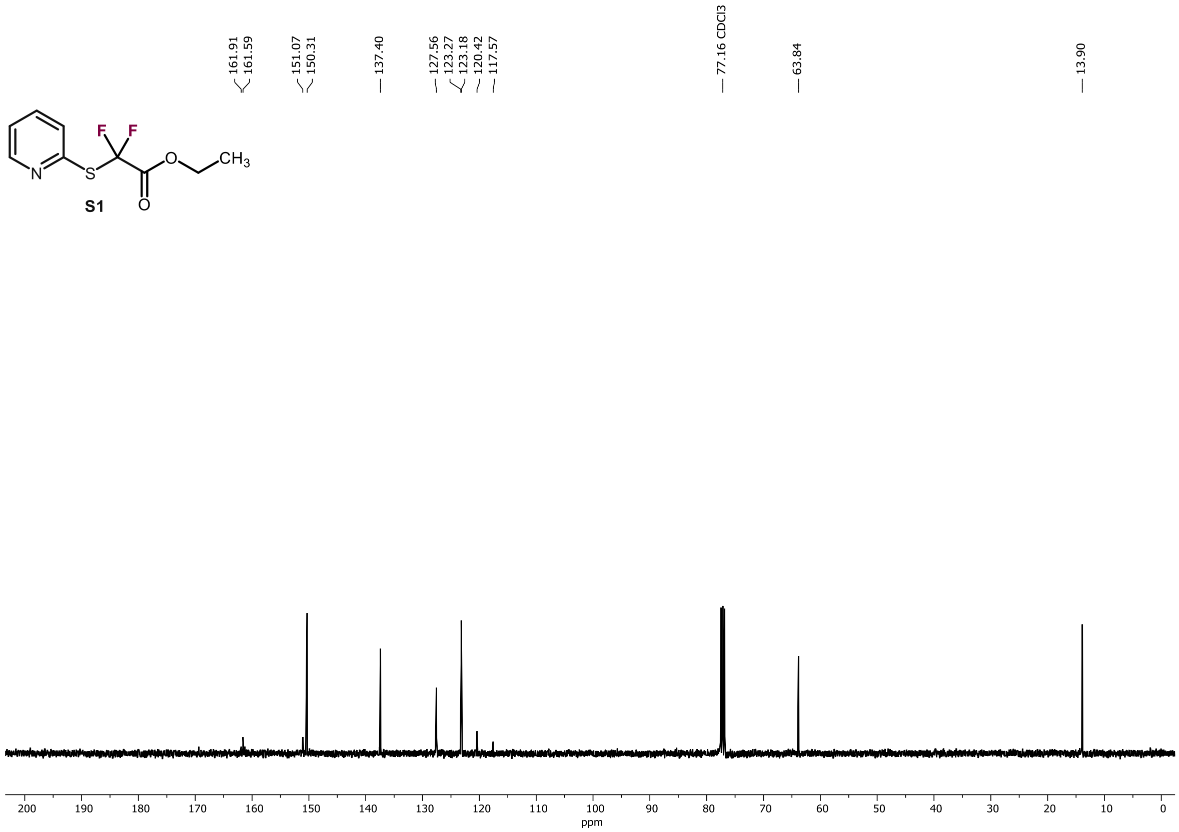

^{19}F NMR (376.5 MHz, $CDCl_3$) of **S1**

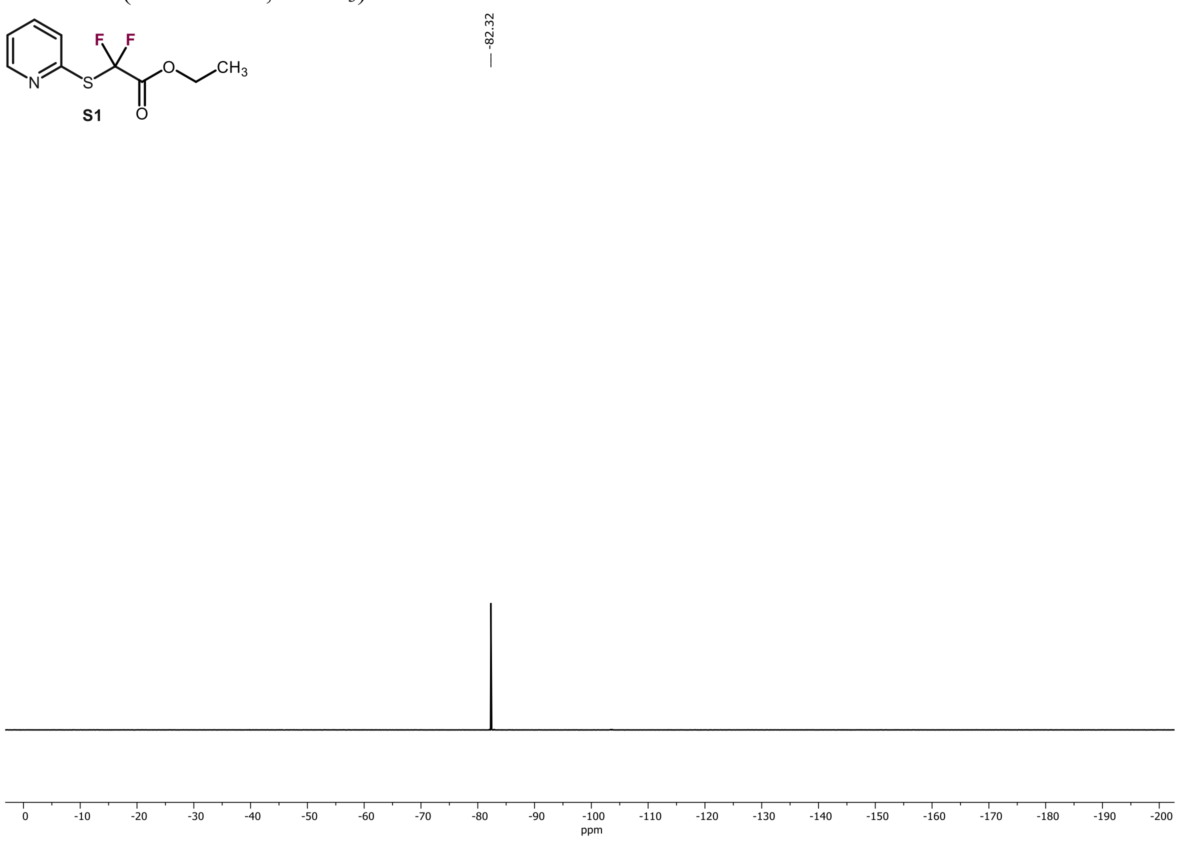

^{1}H NMR (400 MHz, $CDCl_3$) of **S2**

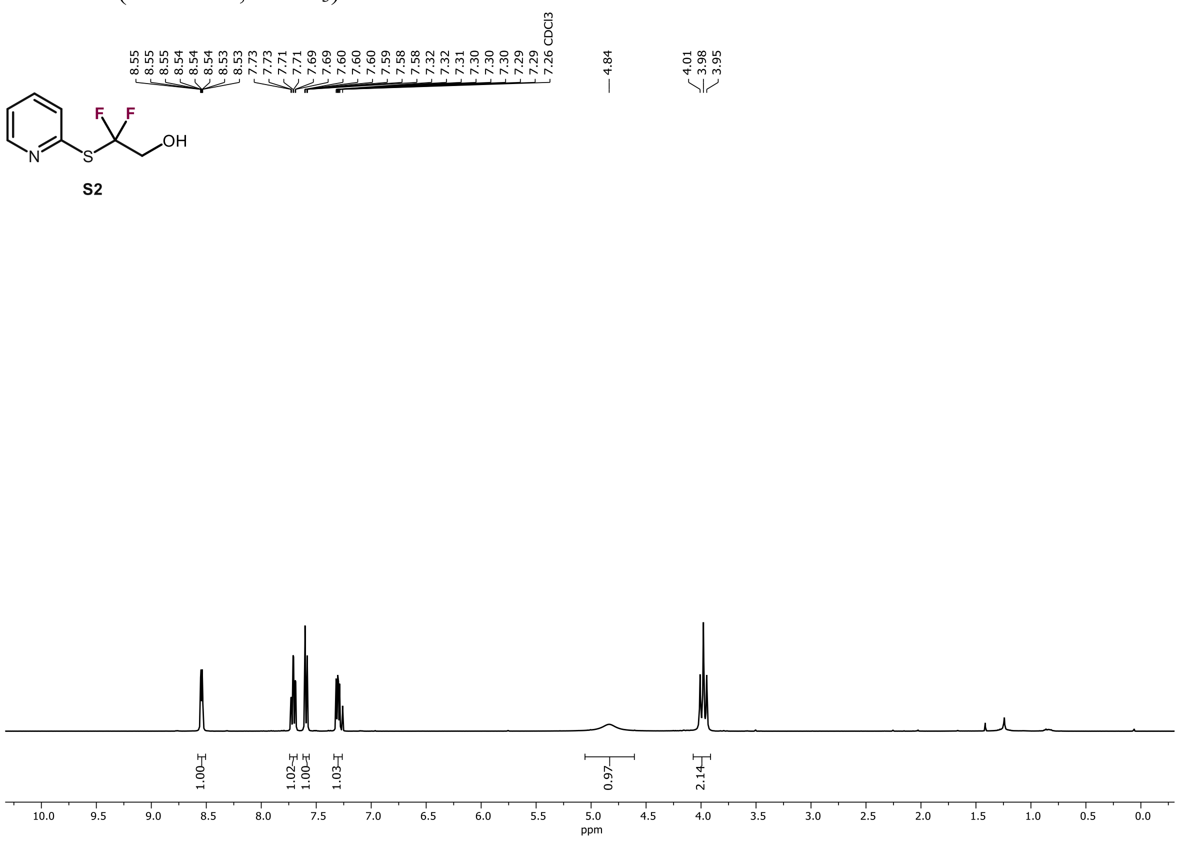

$^{13}C\{^{1}H\}$ NMR (100.6 MHz, $CDCl_3$) of **S2**

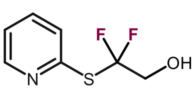

S2

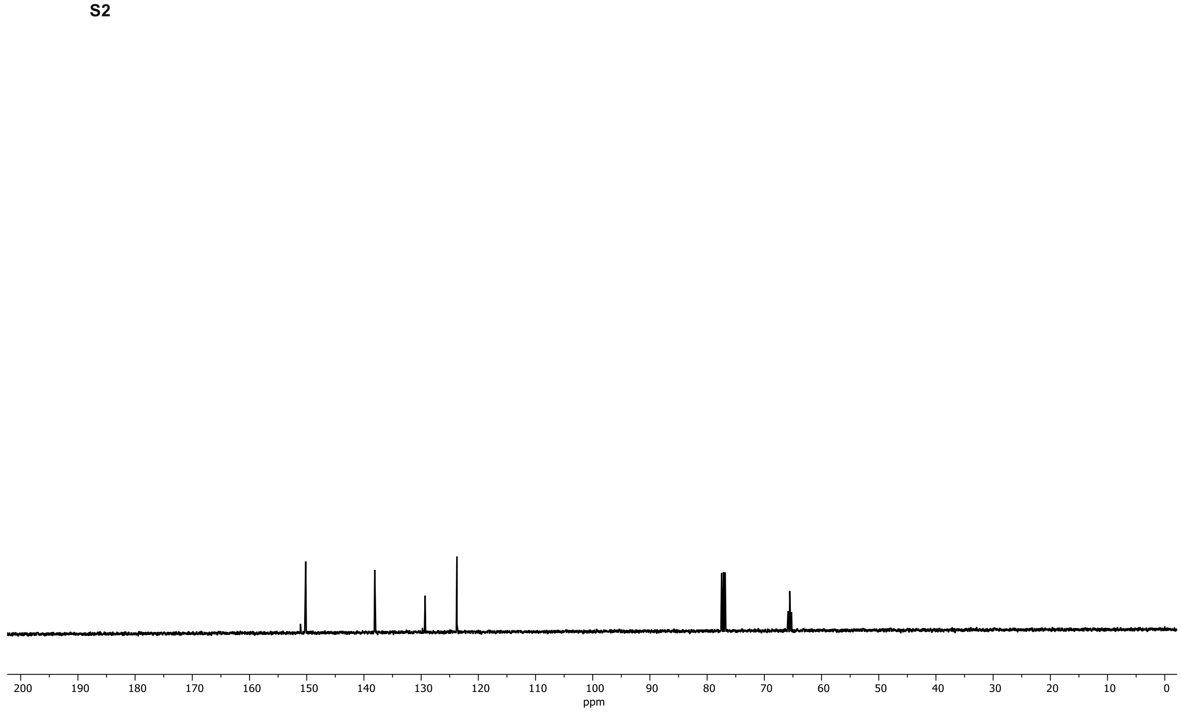

^{19}F NMR (376.5 MHz, $CDCl_3$) of **S2**

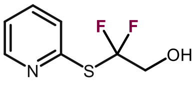

S2

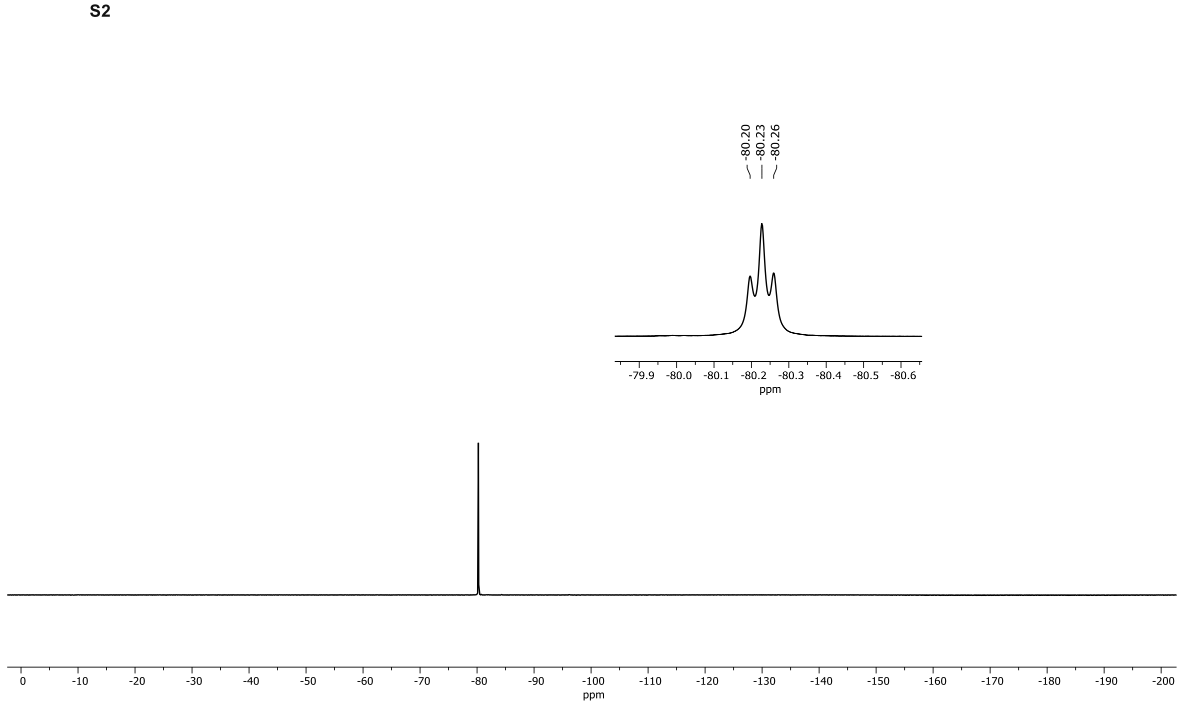

^{1}H NMR (400 MHz, $CDCl_3$) of **S3**

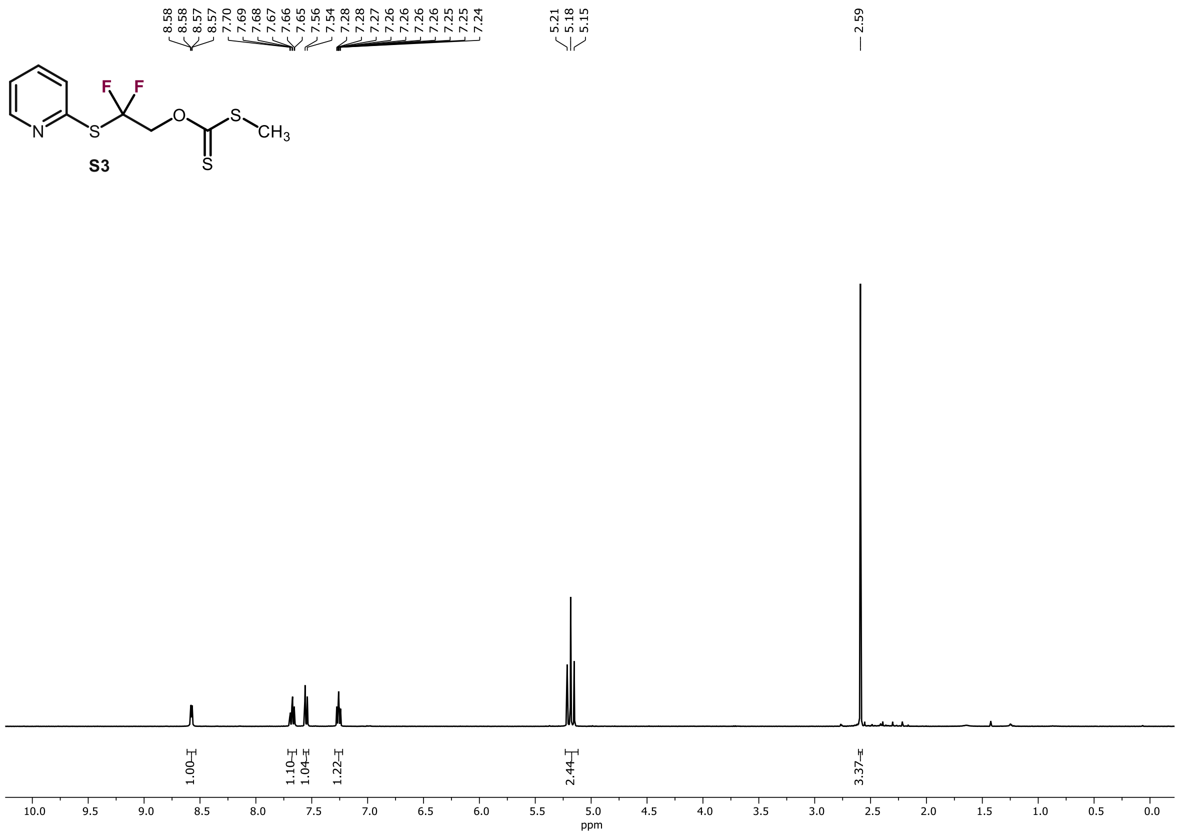

^{13}C{^{1}H} NMR (100.6 MHz, $CDCl_3$) of **S3**

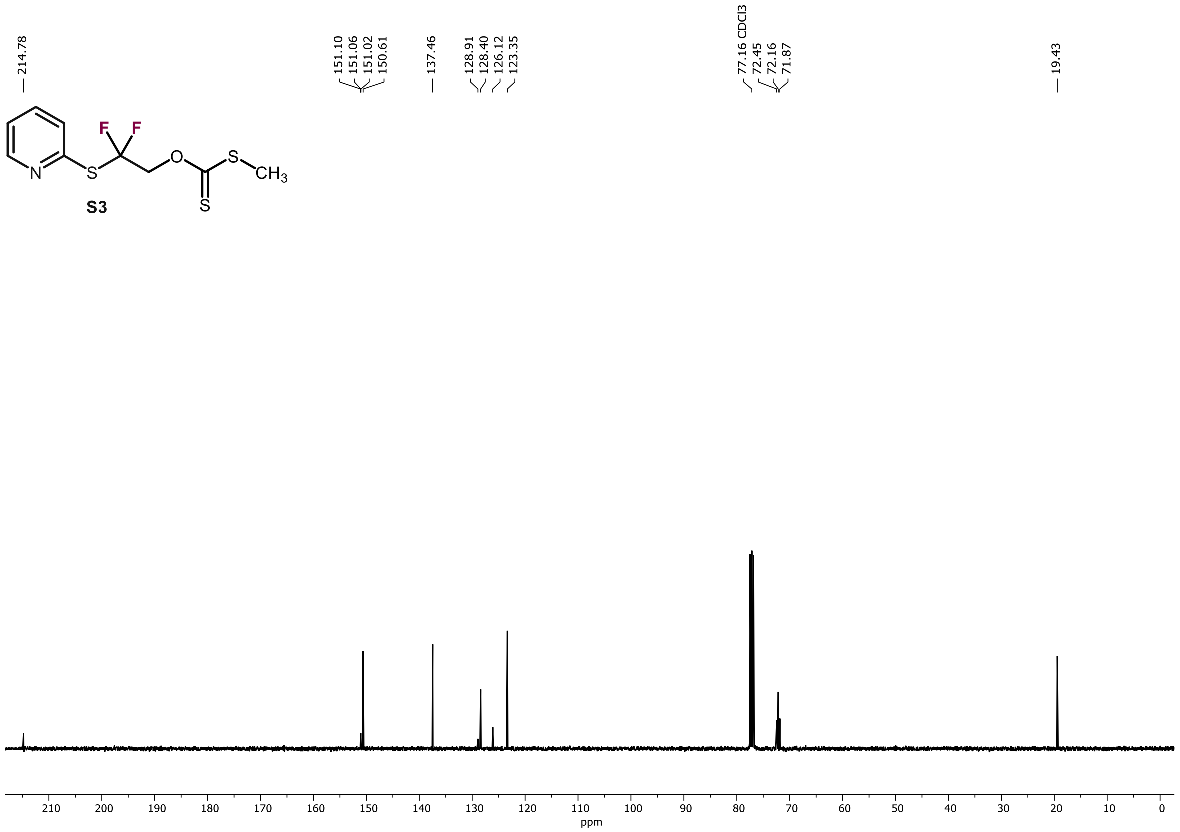

^{19}F NMR (376.5 MHz, $CDCl_3$) of **S3**

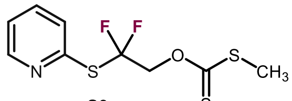

S3

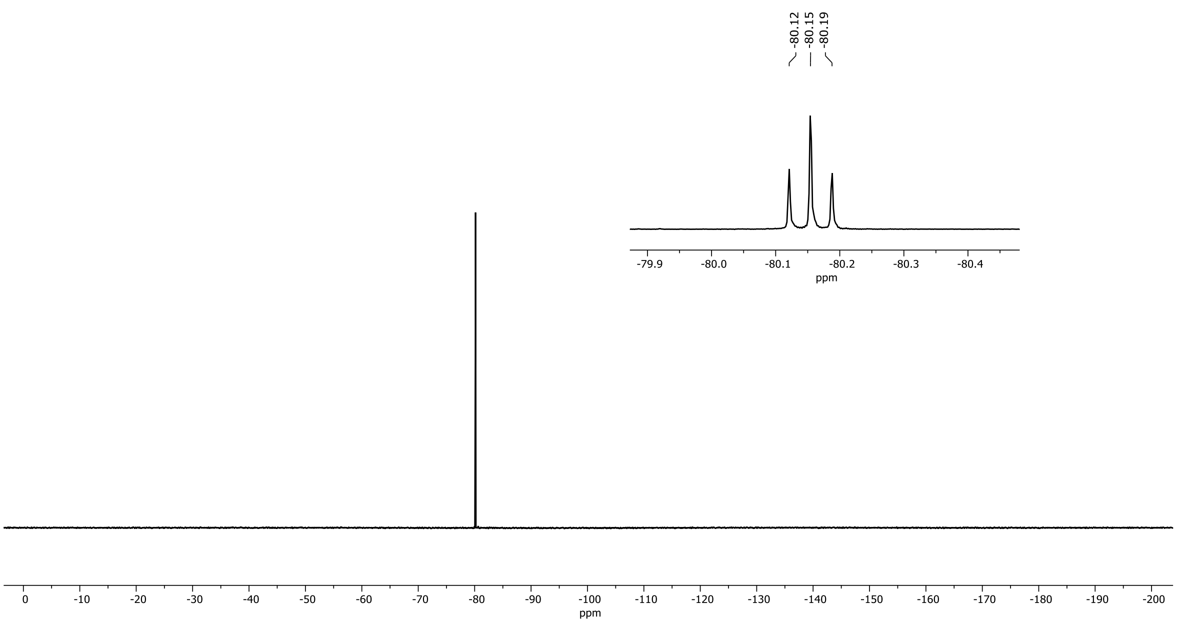

17. Purity of compounds 1, 5, 10, and 15

An HPLC apparatus (LC 1200 Series from Agilent Technologies) equipped with a variable wavelength detector (VWD) operating at 230 nm was used. Chromatograms of analogues **1**, **5**, **10** and **15** are shown below with detailed conditions of HPLC (Figure S12–15).

Conditions and column specifications:

- Column: Zorbax Eclipse XDB C18
- Dimensions: 4.6 mm x 150 mm x 5 µm
- Injection volume: 1 µL
- Flow rate: 1 mL/min
- Temperature: 30 °C
- Detection: G1314B Agilent 1200 Series Variable Wavelength Detector at 230 nm
- Mobile phase:
  A: 1% TFA in H_2O
  B: 1% TFA in Acetonitrile
- Gradient method: A:B 95:5 to 10:90 over 8 min, isocratic 10:90 over 12 min, and 10:90 to 95:5 over 18 min.

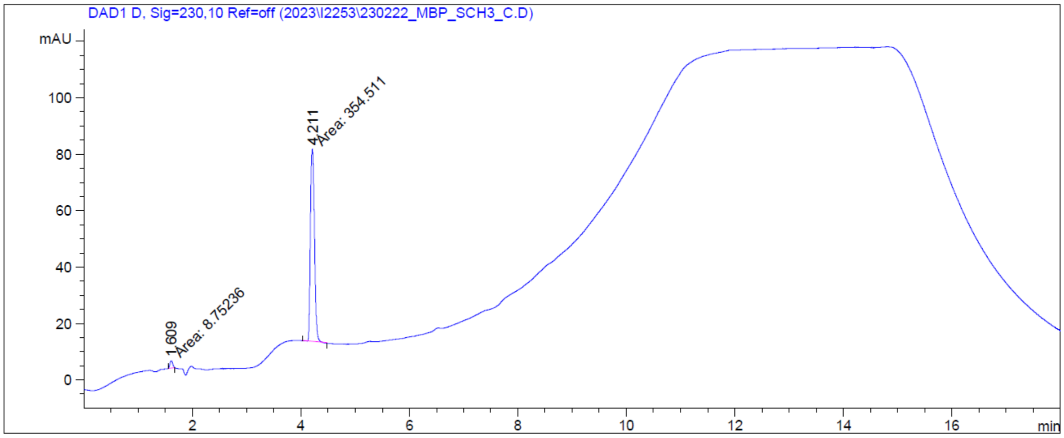

Signal 1: DAD1 D, Sig=230,10 Ref=off

Peak #	RetTime [min]	Type	Width [min]	Area [mAU*s]	Height [mAU]	Area %
1	1.609	MM	0.0576	8.75236	2.53151	2.4094
2	4.211	MM	0.0868	354.51059	68.08441	97.5906
Totals :				363.26295	70.61593	

Figure S12. RP-HPLC-VWD analysis of **1**

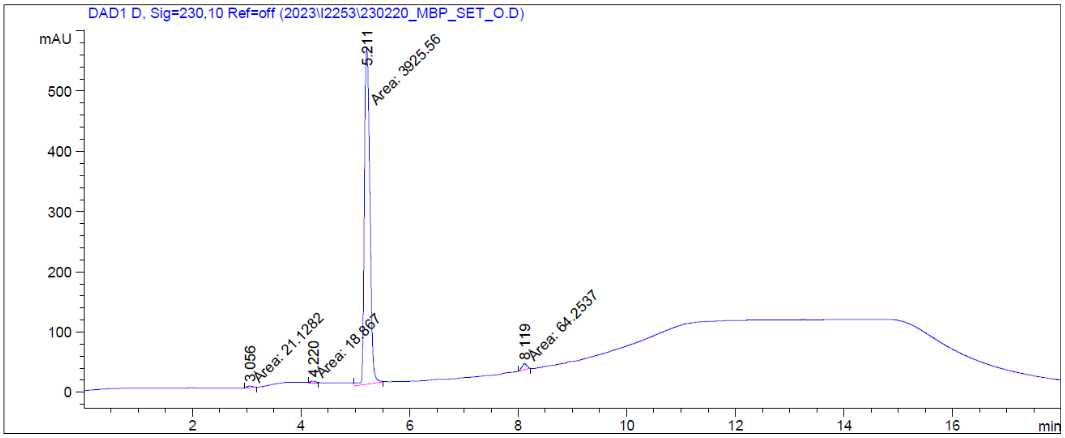


```
Signal 1: DAD1 D, Sig=230,10 Ref=off

Peak RetTime Type  Width      Area       Height      Area
  #    [min]        [min]    [mAU*s]     [mAU]        %
----|-------|----|-------|----------|----------|--------|
   1    3.056 MM    0.1175    21.12817    2.99803    0.5243
   2    4.220 MM    0.0988    18.86695    3.18344    0.4682
   3    5.211 MM    0.1167 3925.55518  560.65717   97.4131
   4    8.119 MM    0.1086    64.25369    9.86288    1.5945

Totals :                    4029.80399  576.70152
```

Figure S13. RP-HPLC-VWD analysis of **5**

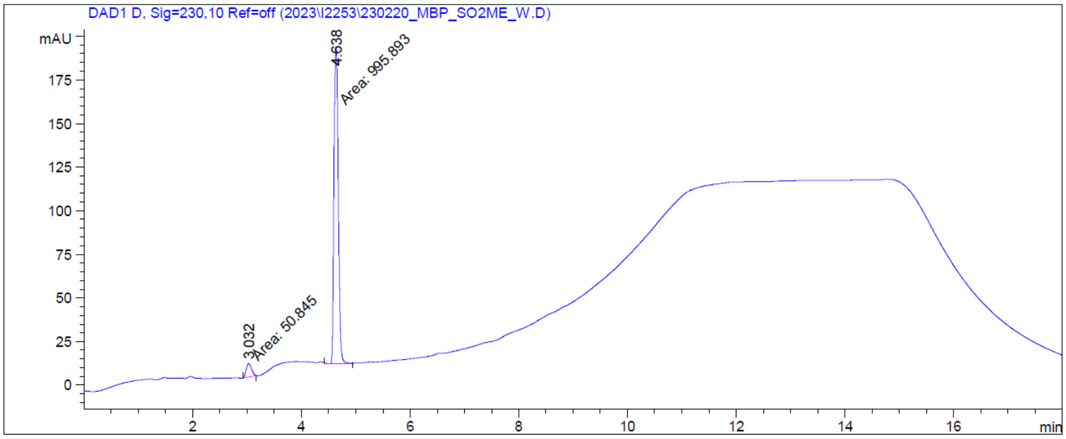


```
Signal 1: DAD1 D, Sig=230,10 Ref=off

Peak RetTime Type  Width      Area       Height      Area
  #    [min]        [min]    [mAU*s]     [mAU]        %
----|-------|----|-------|----------|----------|--------|
   1    3.032 MM    0.1112    50.84498    7.62289    4.8575
   2    4.638 MM    0.0914   995.89252  181.51433   95.1425

Totals :                    1046.73750  189.13722
```

Figure S14. RP-HPLC-VWD analysis of **10**

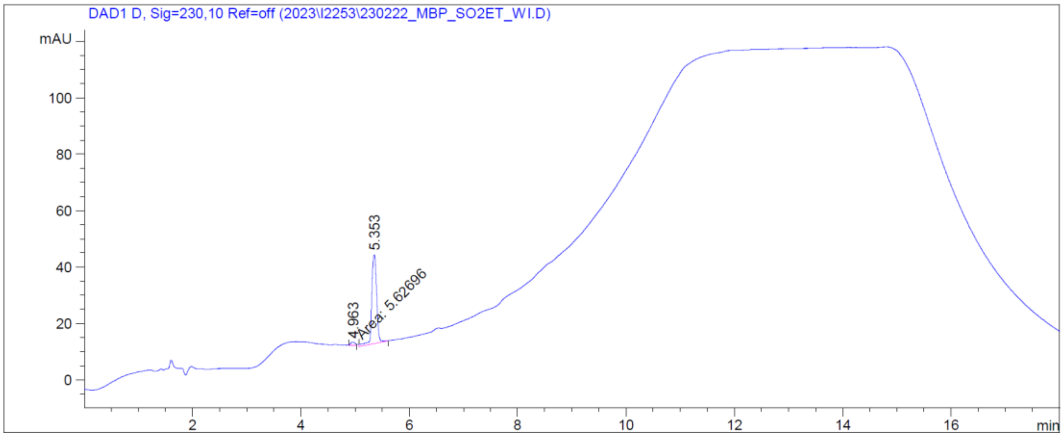

Signal 1: DAD1 D, Sig=230,10 Ref=off

Peak #	RetTime [min]	Type	Width [min]	Area [mAU*s]	Height [mAU]	Area %
1	4.963	MM	0.0895	5.62696	1.04736	2.7473
2	5.353	VB	0.1017	199.19107	31.53759	97.2527
Totals :				204.81803	32.58496	

Figure S15. RP-HPLC-VWD analysis of **15**

18. References

1. Reed, A. E.; Weinhold, F. Natural bond orbital analysis of near-Hartree-Fock water dimer. *J. Chem. Phys.* **1983,** *78*, 4066–4073.
2. Reed, A. E.; Weinstock, R. B.; F. Weinhold. Natural population analysis. *J. Phys. Chem.* **1985**, *83*, 735–746.
3. Ertl, P.; Rohde, B.; Selzer, P. Fast Calculation of Molecular Polar Surface Area as a Sum of Fragment-Based Cheminformatics, *Novartis Pharma AG*, **2000**, *43*, 3714–3717.
4. Schaftenaar, G.; de Vlieg, J. Quantum mechanical polar surface area. *J. Comput. Aided Mol. Des.* **2012**, *26*, 311–318.
5. Schaftenaar, G.; Noordik, J.H. Molden: a pre- and post-processing program for molecular and electronic structures*. *J. Comput. Aided Mol. Des.* **2000**, *14*, 123–134.
6. Mannhold, R.; van de Waterbeemd, H. Substructure and whole molecule approaches for calculating PP. *J. Comput. Aided Mol. Des.* **2001**, *15*, 337–354.
7. Mracec M.; Mracec M.; Bologa C.; Simon Z. Significance of MTD and other descriptors in lipophilicity models for chlorinated aromatic compounds. *SAR QSAR Environ Res.* **2001**, *12*, 143–158.
8. Müller, J.; Martins, A.; Csábi, J.; Fenyvesi, F.; Könczöl, Á.; Hunyadi A.; Balogh, G. T. BBB penetration-targeting physicochemical lead selection: Ecdysteroids as chemo-sensitizers against CNS tumors. *Eur. J. Pharm. Sci.* **2017**, *96*, 571–577.
9. Tomasz, G.; Jan, J.J.; Walerian, P. Correlations between no observed effect level and selected parameters of the chemical structure for veterinary drugs. *Toxicol. Vitro.* **2010**, *24*, 953–959.
10. Remko, M. Acidity, lipophilicity, solubility, absorption, and polar surface area of some ACE inhibitors. *Chem. Pap.* **2007**, *61*, 133–141.
11. Remko, M. Theoretical study of molecular structure, pKa, lipophilicity, solubility, absorption, and polar surface area of some hypoglycemic agents. *J. Mol. Struct. Theochem.* **2009**, *897*, 73–82.
12. Remko, M.; Remková, A.; Broer, R. A Comparative Study of Molecular Structure, pKa, Lipophilicity, Solubility, Absorption and Polar Surface Area of Some Antiplatelet Drugs. *Int. J. Mol. Sci.* **2016**, 17, 388.
13. Remko, M.; Remková, A.; Broer, R. Theoretical Study of Molecular Structure and Physicochemical Properties of Novel Factor Xa Inhibitors and Dual Factor Xa and Factor IIa Inhibitors. *Molecules.* **2016**, *21*, 185.
14. Remko, M.; Swart, M.; Bickelhaupt, F.M. Theoretical study of structure, pKa, lipophilicity, solubility, absorption, and polar surface area of some centrally acting antihypertensives. *Bioorg. Med. Chem.* **2006**, *14,* 1715–1728.
15. Michalík, M.; Lukeš, V. The validation of quantum chemical lipophilicity prediction of alcohols. *Acta Chimica Slovaca* **2016**, *9,* 89–94.
16. Vlahović, F. Ž.; Ivanović, S.; Zlatar, M.; Gruden, M. Density Functional Theory Calculation of Lipophilicity for Organophosphate Type Pesticides. *J. Serb. Chem. Soc.* **2017**, *82,* 1369–1378.
17. Magomedov, K.E.; Zeynalov, R. Z.; Suleymanov, S. I.; Tataeva, S. D.; Magomedova, V. S. Calculation of Lipophilicity of Organophosphate Pesticides Using Density Functional Theory. *Membranes.* **2022**, *12*, 632.
18. Davies, R. H.; Sheard, B.; Taylor, P. J. Conformation, partition, and drug design. *J. Pharm. Sci.* **1979**, *68*, 396–397.
19. Noszál, B.; Kraszni, M. Conformer-Specific Partition Coefficient: Theory and Determination. *J. Phys. Chem. B* **2002**, *106*, 1066–1068.
20. Kraszni, M.; Bányai, I.; Noszál, B. Determination of Conformer-Specific Partition Coefficients in Octanol/Water Systems. *J. Med. Chem.* **2003**, *46*, 2241–2245.

21. Linclau, B.; Wang, Z.; Jeffries, B.; Graton, J.; Carbajo, R. J.; Sinnaeve, D.; Le Questel, J.-Y.; Scott, J. S.; Chiarparin, E. Relating Conformational Equilibria to Conformer-Specific Lipophilicities: New Opportunities in Drug Discovery. *Angew. Chem. Int. Ed.* **2022**, *61*, e202114862.
22. Muehlbacher, M.; Kerdawy, A.; Kramer, C.; Hudson, B.; Clark. T. Conformation-Dependent QSPR Models: LogPow. *J. Chem. Inf. Model.* **2011**, *51*, 2408–2416.
23. Jia, Q.; Ni, Y.; Liu, Z.; Gu, X.; Cui, Z.; Fan, M.; Zhu, Q.; Wang, Y.; Ma, J. Fast Prediction of Lipophilicity of Organofluorine Molecules: Deep Learning-Derived Polarity Characters and Experimental Tests. *J. Chem. Inf. Model.* **2022**, *62*, 4928–4936.
24. Etter, M. C. Encoding and Decoding Hydrogen-Bond Patterns of Organic Compounds. *Acc. Chem. Res.* **1990**, *23*, 120–126.
25. U. Koch and P. L. A. Popelier. Characterization of C-H-0 Hydrogen Bonds on the Basis of the Charge Density. *J. Phys. Chem.* **1995**, *99*, 9747–9754.
26. Columbus, I.; Ghindes-Azaria, L.; Herzog, I. M.; Blum, E.; Parvari, G.; Eichen, Y.; Cohen, Y.; Gershonov, E.; Drug, E.; Saphier, S.; Elias, S.; Smolkin, B.; Zafrani, Y. Species-Specific Lipophilicities of Fluorinated Diketones in Complex Equilibria Systems and Their Potential as Multifaceted Reversible Covalent Warheads. *Commun. Chem.* **2023**, *6*, 197.
27. Jeffries, B.; Wang, Z.; Felstead, H. R.; Le Questel, J.-Y.; Scott, J. S.; Chiarparin, E.; Graton, J.; Linclau, B. Systematic Investigation of Lipophilicity Modulation by Aliphatic Fluorination Motifs. *J. Med. Chem.* **2020**, *63*, 100–118.
28. Liu, Z.; Wang, P.; Wold, E. A.; Song, Q.; Zhao, C.; Wang, C.; Zhou, J. Small-Molecule Inhibitors Targeting the Canonical WNT Signaling Pathway for the Treatment of Cancer. *J. Med. Chem.* **2021**, *64*, 4257–4288.
29. Saphier, S.; Katalan, S.; Yacov, G.; Berliner, A.; Redy-Keisar, O.; Fridkin, G.; Ghindes-Azaria, L.; Columbus, I.; Pevzner, A.; Drug, E.; Prihed, H.; Gershonov, E.; Eichen, Y.; Elias, S.; Parvari, G.; Zafrani, Y. Placing CF_2 in the Center: Major Physicochemical Changes Upon a Minor Structural Alteration in Gem-Difunctional Compounds. *Chem. Eur. J.* **2023**, *29*, e202202939.
30. Finkbeiner, P.; Hehn, J. P.; Gnamm, C. .Phosphine Oxides from a Medicinal Chemist's Perspective: Physicochemical and in Vitro Parameters Relevant for Drug Discovery. **2020**, *63*, 7081–7107.
31. Saphier, S.; Zafrani, Y. CF_2H: A Fascinating Group for Application in Drug Development Enabling Modulation of Many Molecular Properties. *Future Med. Chem.* **2024**, *16*, 1181–1184
32. Bader, R. F. Atoms in Molecules; *Wiley Online Library*: **1990**.
33. Wiberg, K.B. Atoms in Molecular Environments. *Springer Link.* **1990**.
34. Lu, W.; Chan, M. C. W.; Zhu, N.; Che, C.-M.; He, Z.; Wong, K.-Y. Structural Basis for Vapoluminescent Organoplatinum Materials Derived from Noncovalent Interactions as Recognition Components. *Chem. Eur. J.* **2003**, *9*, 6155–6166.
35. Becke, A. D.; Edgecombe, K. E. A simple measure of electron localization in atomic and molecular systems. *J. Chem. Phys.* **1990**, *92*, 5397–5403.
36. Tomita, R.; Al-Maharik, N.; Rodil, A.; Bühl, M.; O'Hagan, D. Synthesis of Aryl α,α-Difluoroethyl Thioethers a Novel Structure Motif in Organic Chemistry, and Extending to Aryl α,α-Difluoro Oxyethers. *Org. Biomol. Chem.* **2018**, *16*, 1113–1117.
